# Rhein alleviates MPTP-induced Parkinson’s disease by suppressing neuroinflammation via MAPK/IκB pathway

**DOI:** 10.3389/fnins.2024.1396345

**Published:** 2024-06-12

**Authors:** Xin Qin, Shu Wang, Juan Huang, Binbin Hu, Xingyan Yang, Liying Liang, Rui Zhou, Wei Huang

**Affiliations:** ^1^Department of Neurology, The Second Affiliated Hospital of Nanchang University, Nanchang, China; ^2^Department of Neurology, Yichang Central People’s Hospital, Yichang, China; ^3^Jiangxi Province Key Laboratory of Molecular Medicine, Nanchang, China

**Keywords:** rhein, Parkinson’s disease, neuroinflammation, MAPK/IκB, MPTP

## Abstract

**Background:**

Parkinson’s disease (PD) is a common neurodegenerative disease with a rapid increase in incidence in recent years. Existing treatments cannot slow or stop the progression of PD. It was proposed that neuroinflammation leads to neuronal death, making targeting neuroinflammation a promising therapeutic strategy. Our previous studies have demonstrated that rhein protects neurons *in vitro* by inhibiting neuroinflammation, and it has been found to exhibit neuroprotective effects in Alzheimer’s disease and epilepsy, but its neuroprotective mechanisms and effects on PD are still unclear.

**Methods:**

PD animal model was induced by 1-methyl-4-phenyl-1,2,3, 6-tetrahydropyridine (MPTP). ELISA, RT-qPCR, western blot and Immunofluorescence were used to detect the levels of inflammatory cytokines and M1 polarization markers. The protein expression levels of signaling pathways were measured by western blot. Hematoxylin–eosin (HE) staining showed that rhein did not damage the liver and kidney. Two behavioral tests, pole test and rotarod test, were used to evaluate the improvement effect of rhein on movement disorders. The number of neurons in the substantia nigra was evaluated by Nissl staining. Immunohistochemistry and western blot were used to detect tyrosine hydroxylase (TH) and α-synuclein.

**Results:**

Rhein inhibited the activation of MAPK/IκB signaling pathway and reduced the levels of pro-inflammatory cytokines (IL-1β, IL-6 and TNF-α) and M1 polarization markers of microglia *in vivo*. In a mouse model of PD, rhein ameliorated movement disorders, reduced dopaminergic neuron damage and α-synuclein deposition.

**Conclusion:**

Rhein inhibits neuroinflammation through MAPK/IκB signaling pathway, thereby reducing neurodegeneration, α-synuclein deposition, and improving movement disorders in Parkinson’s disease.

## 1 Introduction

Parkinson’s disease (PD) is a common neurodegenerative disorder that affects approximately 1% of the global population over the age of 60 years, and its incidence has increased rapidly in recent years (GBD 2016 Neurology Collaborators, 2019; Bloem et al., 2021). The pathogenesis of PD is mainly due to the degeneration of dopaminergic neurons and striatal projection system, resulting in the reduction of dopamine content in the brain (Bloem et al., 2021). Neurodegeneration in PD may be related to neuroinflammation, abnormal aggregation of α-synuclein, oxidative stress, mitochondrial dysfunction, abnormal iron metabolism, abnormal brain structure and metabolism, changes in intestinal microbial composition, excitotoxicity, protein degradation disorders and other factors (Si et al., 2020; Shrestha et al., 2021). These factors combine to accelerate the death of dopaminergic neurons. Among them, neuroinflammation has received a lot of attention as it can cause neuronal death and play an important role in the pathogenesis of PD (Kam et al., 2020).

Early studies have found that there were HLA-DR+ reactive microglia in *post mortem* tissue from PD patients (McGeer et al., 1988), some PD related genes are involved in immune regulation (Li et al., 2019), and PD patients share some genetic variants with patients with immune diseases and inflammatory diseases (such as Crohn’s disease) (Hui et al., 2018). From these direct and indirect evidence, inflammation is closely related to PD. According to relevant literature, inflammation in PD originates from the gut and then spreads to the blood circulation. Circulating proinflammatory cytokines and pro-inflammatory cells increase, then cross the blood–brain barrier and enter the brain to form chronic neuroinflammation, in which proinflammatory cytokines mainly IL-1β, IL-6 and TNF-α damage neurons (Tansey et al., 2022). Damaged neurons release pathological α-synuclein (α-Syn), which activates microglia and astrocytes to produce proinflammatory cytokines and unknown toxins, which damage neurons again, forming a vicious cycle of neuroinflammation and neuronal damage (Kam et al., 2020). Therefore, it has been suggested that neuroinflammation interacts with genetic and environmental factors to participate in the pathogenesis of PD (Tansey et al., 2022). Several studies have confirmed that inhibition of MAPK and NFκB/IκB signaling pathways can reduce neuroinflammation (Park et al., 2011; Shabab et al., 2017; Zheng et al., 2020). In addition, inhibition of MAPK and NFκB signaling pathways was found to play a neuroprotective role in central nervous system (CNS) diseases such as PD and epilepsy (Mattson and Camandola, 2001; Singh and Singh, 2020; Yu et al., 2021). Thus, it is hypothesized that reducing neuroinflammation by inhibiting MAPK and NFκB signaling pathways can alleviate neurodegeneration.

Rhein is an anthraquinone compound, which is isolated from traditional Chinese medicine such as aloevera, rhubarb, Cassia seed, and multiflorum. It exerts a great diversity of pharmacological functions, suppression on bacteria, inflammation, tumor, oxidation and fibrosis, protection to liver and kidney, for example (Fernand et al., 2011; Irshad et al., 2011; Sun et al., 2016; Ge et al., 2017). The anti-inflammatory property of rhein has been validated in multiple cell and animal studies (Ge et al., 2017; Antonisamy et al., 2019). In addition, rhein has been found to exhibit favorable anti-inflammatory properties in various disease models such as ulcerative colitis, rheumatoid, osteoarthritis, renal injury, and traumatic brain injury (Wang et al., 2015; Yu et al., 2015; Hu et al., 2020; Ebada et al., 2021; Liu et al., 2021; Dong et al., 2022). As the only anthraquinones that can enter the blood–brain barrier (Wang et al., 2015, 2016), rhein has been shown to play an anti-neuroinflammatory role by regulating a variety of inflammatory signaling pathways (Zheng et al., 2020). It is reported that rhein can reduce Aβ deposition and neuroinflammation, furthermore alleviate cognitive impairment of Alzheimer’s disease in animals (Yin et al., 2022). Rhein attenuates epilepsy and exerts neuroprotective effect by inhibiting TLR4-NFκB signaling pathway (Yu et al., 2021). Given the role of rhein in inhibiting neuroinflammation and protecting neurons in central nervous system diseases, it is necessary to investigate the mechanism by which rhein protects neurons and its effect on PD, which also has neuroinflammation.

Due to the complex pathogenesis of PD, the existing treatment methods cannot slow down or prevent the progress of it, and further research is needed to provide a basis for the treatment of PD. The aim of this study is to explore the neuroprotective mechanism of rhein *in vitro*, and to investigate the effect of rhein on MPTP-induced PD mouse model, so as to provide help for the treatment of PD.

## 2 Materials and methods

### 2.1 Animals and treatments

**Figure 1:**
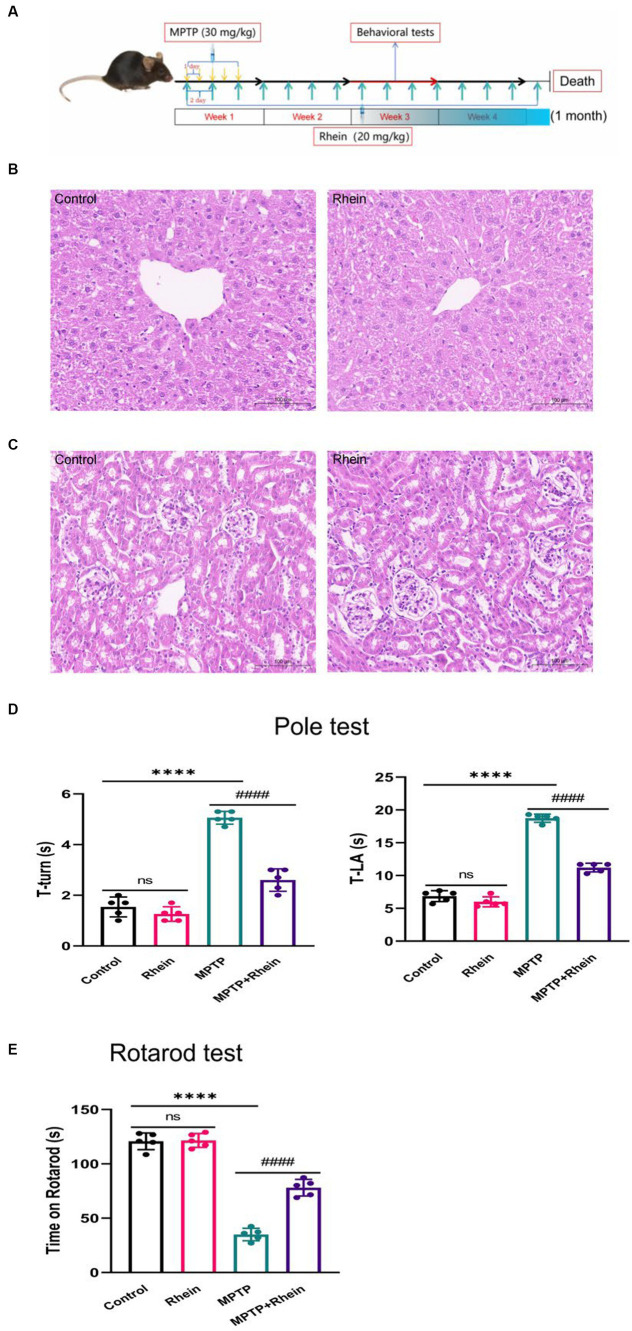
Rhein treatments improved motor function in MPTP-treated mice. **(A)** Flow chart of animal experiment design. The liver **(B)** and kidney **(C)** of mice were not damaged by rhein treatments in HE staining (*n* = 5). **(D)** For the pole test, the time for mouse to turn-back and reach the bottom of the pole was recorded and analyzed (*n* = 5). **(E)** Latency to fall in the rotarod test was recorded and analyzed (*n* = 5). Data are presented as the mean ± SD. ****p < 0.0001 compared with the control group; ####*p* < 0.0001 compared with the MPTP group.

Male C57BL/6 mice, aged 8–10 weeks, weight 22-30 g, purchased from Changsha Tianqin Biotechnology Co., LTD. The mice were kept at room temperature (22–25°C), with light and dark cycle at 12:12 h, and had free access to food and water. The mice were randomly divided into 4 groups, there were 10 mice in each group: control group, the same volume of PBS as MPTP group was injected intraperitoneally for 5 days, the same as MPTP regimen; rhein group, treated with rhein alone; MPTP group, PD models were induced by MPTP; MPTP+Rhein group, PD models were induced by MPTP, combined with rhein therapy. The animal protocol is shown in flow chart of animal experiment design (Figure 1A): mice were intraperitoneally injected with 30 mg/kg body weight MPTP (Beyotime, Shanghai, China) once a day for 5 days to establish a subacute Parkinson’s disease model (Jackson-Lewis and Przedborski, 2007); mice were injected 20 mg/kg body weight rhein via the tail vein (CAS: 478–43-3, HPLC ≥98%, Yuanye Bio-Technology, Shanghai, China) every 2 days for 1 month, the vehicle used for rhein was PBS, the therapeutic dose of rhein was referred to the previous study (Yin et al., 2022); behavioral tests were performed during the third week of treatment; after 1 month of treatment, different groups of mice were sacrificed by cervical dislocation for treatment evaluation. Fresh tissues such as substantia nigra, striatum, and serum collected from mice were stored at −80°C. All experimental procedures were approved by the Experimental Animal Welfare Ethics Committee of Nanchang University. Animal suffering and the number of animals used were minimized.

### 2.2 Behavioral tests

#### 2.2.1 Pole test

The motor balance, coordination and retardation of the mice were evaluated by pole test at the third week of treatment. Five mice were tested in each group. Referring to previous studies and making minor adjustments (Kim et al., 2019), a wooden rod about 75 cm long and 9 mm in diameter was used, its surface was wrapped in gauze, and the board base was connected below to make a test device. Before the actual test, the mice were trained for two consecutive days. During the test, the mice were placed head-first 7.5 cm from the top of the pole, and the time it took the mice to turn to head down (T-turn) and climb down to the base (T-LA) was recorded. Each mouse was tested for 3 consecutive times at 1 min intervals, and the average time was taken for analysis. After each test, washed the sticks with 70% ethanol.

#### 2.2.2 Rotarod test

The rotarod test was used to assess motor coordination and balance in the mice during the third week of treatment. Also five mice were tested in each group. Referring to the previous study (Kim et al., 2019), the mice were trained for 3 days before the experiment, and on the fourth day the mice were placed on an accelerated rotating cylinder, where the speed slowly increased from 4 revolutions per minute to 40 revolutions per minute over 5 minutes, and the latency period for each mouse to fall off the spinner was recorded. The mice were tested three times, each at least 30 min apart. If the animal fell off the step, or grabbed onto the device and spun 2 consecutive turns without attempting to walk on the step, the trial ended. The average latency time of three times was used for analysis. After each test, cleaned the instrument with 70% ethanol.

### 2.3 RT-qPCR

In this experiment, 3 mice per group were assayed, and each sample run in triplicate. Total RNA was extracted from substantia nigra and corpus striatum using Trizol reagent according to the manufacturer’s instructions, and RNA concentrations were quantified by spectrophotometry using a NanoDrop One spectrophotometer (Thermo Fisher Scientific, Waltham, MA, USA). 2ug of total RNA was reverse transcribed into cDNA using a PrimeScript RT reverse transcription kit (TransGen Biotech Co., Ltd., Beijing, China) in a reverse transcription PCR machine (Applied Biosystems, USA). RT-qPCR was performed using the SYBR Green kit (Takara Bio, Inc., Otsu, Japan) under the following conditions: Denaturation at 95°C for 30 s, 95°C for 5 s, 60°C for 34 s, 95°C for 15 s, 60°C for 1 min, 95°C for 15 s. The comparative threshold cycle (Ct) method was used for data analysis. RT-qPCR was performed using a 7,500 high-throughput rapid real-time PCR instrument (Thermo Fisher Scientific, Waltham, MA, USA). RT-qPCR primers are shown in Table 1.

**Table 1:** RT - qPCR primers.

	引物名称	序列(5′-3′)
GADPH	Forward Primer	AACGGATTTGGCCGTATTGG
	Reverse Primer	CATTCTCGGCCTTGACTGTG
IL-6	Forward Primer	CTTCTTGGGACTGATGCTGGTGAC
	Reverse Primer	TCTGTTGGGAGTGGTATCCTCTGTG
IL-1 beta	Forward Primer	CACTACAGGCTCCGAGATGAACAAC
	Reverse Primer	TGTCGTTGCTTGGTTCTCCTTGTAC
TNF alpha	Forward Primer	GGACTAGCCAGGAGGGAGAACAG
	Reverse Primer	GCCAGTGAGTGAAAGGGACAGAAC
CD16	Forward Primer	CAGAATGCACACTCTGGAAGC
	Reverse Primer	GGGTCCCTTCGCACATCAG
CD32	Forward Primer	ATGGGAATCCTGCCGTTCCTA
	Reverse Primer	CCGTGAGAACACATGGACAGT
CD86	Forward Primer	TGTTTCCGTGGAGACGCAAG
	Reverse Primer	TTGAGCCTTTGTAAATGGGCA

### 2.4 Elisa

In this experiment, 3 mice per group were assayed. After the treatment, blood was collected from mouse eyeballs, and the blood was left in EP tube at room temperature for more than 1 hour, centrifuged at 3000 rpm for 10 min, and serum was obtained and stored at −80°C. The levels of TNF-α, IL-1β, and IL-6 in serum of mice were measured by ELISA kits (4A Biotech, Beijing, China) according to the manufacturer’s protocols. Then, read the absorbance at 450 nM with an microplate reader (Thermo Fisher Scientific, Waltham, MA, USA). The concentrations of TNF-α, IL-1β, and IL-6 were calculated from the respective standard curves generated simultaneously.

### 2.5 Western blot

In this experiment, 3 mice per group were assayed, 1 sample from each group run across 3 blots. Proteins were extracted from substantia nigra and corpus striatum by homogenization in RIPA lysis buffer. Protein concentrations were determined using the BCA assay kit. Proteins (10 μL/lane) were separated by 10% SDS-PAGE, transferred to polyvinylidene fluoride (PVDF) membrane (Millipore, Billerica, MA, USA), Nonspecific binding sites were blocked with BSA blocking solution (Solarbio, Beijing, China) for 2 h at room temperature, followed by incubation with the following primary antibodies overnight at 4°C: pP38 (1:750, Wanleibio, Shenyang, China), P38 (1:4000, Proteintech, Wuhan, China), pJNK (1:2000, Proteintech, Wuhan, China), JNK (1:10000, Proteintech, Wuhan, China), pIκB (1:10000, Abcam, Cambridge, UK), IκB (1:10000, Proteintech, Wuhan, China), Iba1 (1:1000, Proteintech, Wuhan, China), CD86 (1:1000, Proteintech, Wuhan, China), TH (1:2000, Proteintech, Wuhan, China), Alpha Synuclein (1:500, Proteintech, Wuhan, China) and GAPDH (1:10000, Proteintech, Wuhan, China). Then using horseradish peroxidase (HRP) coupling resistance (1:10000, Proteintech, Wuhan, China) incubation 2 h at room temperature. Finally use ECL chemiluminescence fluid soaking (Bioscience, Shanghai, China), and use the ChemiDoc ™ MP imaging system (Bio-Rad Laboratories, Inc., Hercules, CA, USA) to make a visualization. Anti-mouse GAPDH antibody was used as a reference protein for standardization. Image Lab software was used to detect the gray level of protein bands.

### 2.6 Immunofluorescence and immunohistochemistry

For immunofluorescence and immunohistochemistry, 5 mice per group were assessed, and 1 section per mouse was assessed. Remove the whole brain and fix with 4% paraformaldehyde. The brain tissue was embedded in paraffin wax and then coronally sliced at a thickness of 20 μm. Brain sections were fixed with 4% paraformaldehyde for 15 min and then rinsed with PBS containing 0.3% Triton X-100. Sections were blocked in 5% BSA for 2 h and incubated at 4°C overnight with primary antibody. Antibody CD86 (1:50, Proteintech, Wuhan, China) and antibody Iba1 (1:50, Proteintech, Wuhan, China) are used as primary antibodies. After washing with PBS for three times, the brain sections were incubated with fluorescence-coupled secondary antibody (1:100) in room temperature darkness for 2 h. The nuclei were then stained with 4, 6-diaminophenylindole (DAPI). Finally, the brain sections were examined by inverted fluorescence microscope. The mean gray value of immunofluorescence images was analyzed using Image J.

The brain sections were rinsed with PBS containing 0.3% Triton X-100, blocked in 5% BSA for 2 h, and incubated with primary antibody TH (1:2000, Proteintech, Wuhan, China) at 4°C overnight. Staining was carried out using the ABC method (Vector Laboratories), with 3,30-diaminobenzidine (DAB) as the peroxidase substrate. TH-positive dopaminergic (DA) neurons from the substantia nigra and corpus striatum region were examined by microscope. Average optical density (AOD) of immunohistochemical images was analyzed using Image J.

### 2.7 Nissl staining

For Nissl Staining, 5 mice per group were assessed, and 1 section per mouse was assessed. The whole brain was collected, fixed with 4% paraformaldehyde and embedded with paraffin. Then the embedded brain tissue was coronally sliced at a thickness of 20 μm and stained with Nissl staining solution to observe the Nissl body of dopaminergic neurons in substantia nigra. Finally, the Nissl staining sections were photographed under microscope. The mean gray value of Nissl staining images was analyzed using Image J.

### 2.8 Hematoxylin–eosin (HE) staining

For HE Staining, 5 mice per group were assessed, and 1 section per mouse was assessed. Mice were killed 1 month after administration. Liver and kidney tissues were taken, fixed with 4% paraformaldehyde, embedded in paraffin, then sliced at a thickness of 20 μm, stained with hematoxylin–eosin, observed under a microscope and photographed to evaluate the liver and kidney damage of mice caused by rhein.

### 2.9 Statistical analysis

All experiments were independently repeated at least three times. All values were expressed as the mean ± standard deviation (SD) and analyzed using Graphpad Prism 9.0.0 statistical Software (GraphPad Software, California, USA). Comparison among several groups was evaluated using one-way ANOVA followed by a Holm-Šídák’s multiple comparisons test. *p* < 0.05 (*) indicates that the difference is significant when compared with the corresponding control value, *p* < 0.01 (**) indicates very significant difference compared with the corresponding control value. *p* < 0.05 (#) means that the difference is significant compared with the corresponding model value, *p* < 0.01 (##) indicates that the difference is extremely significant compared with the corresponding model value.

## 3 Results

### 3.1 Rhein treatment improved the movement disorder of MPTP-induced PD mice

HE staining was used to observe the effect of rhein treatment on the liver and kidney of mice, and the results showed that rhein treatment had no obvious toxic effect on the liver and kidney of mice (Figures 1B,C).

In order to determine whether rhein treatment can improve the motor deficit of MPTP-induced PD mice, pole test and rotarod test were performed on each group of mice after treatment. Pole test results showed that the MPTP group spent significantly more time on turning around and climbing down the pole than the control group, while the MPTP + Rhein group spent significantly less time than the MPTP group (Figure 1D). Similarly, the results of rotarod test showed that compared with the control group, the time left on the rotating rod was significantly shortened in the MPTP group, and significantly prolonged in the MPTP + Rhein group compared with the MPTP group (Figure 1E). According to the results of mice behavioral tests, rhein treatment can protect the motor function of MPTP-induced PD mice.

### 3.2 Rhein treatment reduced TH+ neuron death and α-synuclein deposition in MPTP-induced PD mice

**Figure 2:**
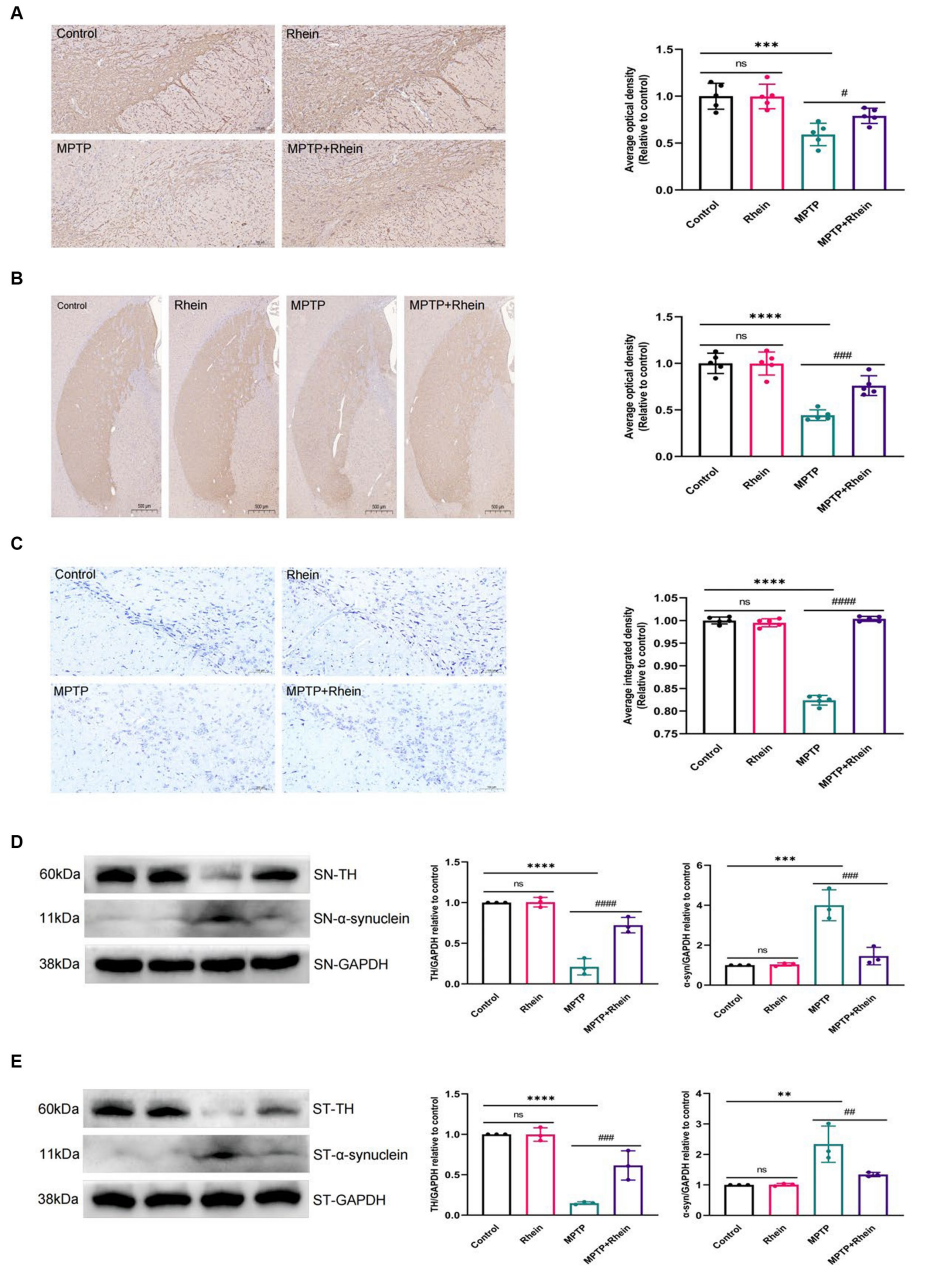
Rhein treatments reduced MPTP-induced loss of TH+ dopaminergic neurons and aberrant α-synuclein aggregation. **(A)** Immunohistochemical staining and quantitative analysis of TH in the SN (*n* = 5). Scale bar: 100 μm. **(B)** Immunohistochemical staining and quantitative analysis of TH in the striatum (*n* = 5). Scale bar: 500 μm. **(C)** Nissl staining and quantitative Nissl positive areas in the SN (*n* = 5). Scale bar: 100 μm. **(D)** Representative western blot bands and quantitative analysis of TH and α-synuclein in the SN by normalization to GAPDH (*n* = 3). **(E)** Representative western blot bands and quantitative analysis of TH and α-synuclein in the striatum by normalization to GAPDH (*n* = 3). Data are presented as the mean ± SD. **p < 0.01, ***p < 0.001, ****p < 0.0001 compared with the control group; #*p* < 0.05, ##*p* < 0.01, ###*p* < 0.001, ####*p* < 0.0001 compared with the MPTP group.

In our previous *in vitro* study, we found that rhein could protect neurons. In order to clarify the role of rhein in MPTP-induced PD mice *in vivo*, further experiments were performed. The results showed that compared with the MPTP group, the MPTP + Rhein group had a significant increase in TH+ neurons and TH expression, a significant reduction in α-synuclein expression of substantia nigra and striatum, and a significant increase in Nissl bodies of substantia nigra neurons (Figure 2). These results indicated that rhein treatment could protect neurons and reduce pathological α-synuclein deposition in MPTP-induced PD mice.

### 3.3 Rhein treatment inhibited inflammation and microglia M1 polarization in MPTP-induced PD mice

**Figure 3:**
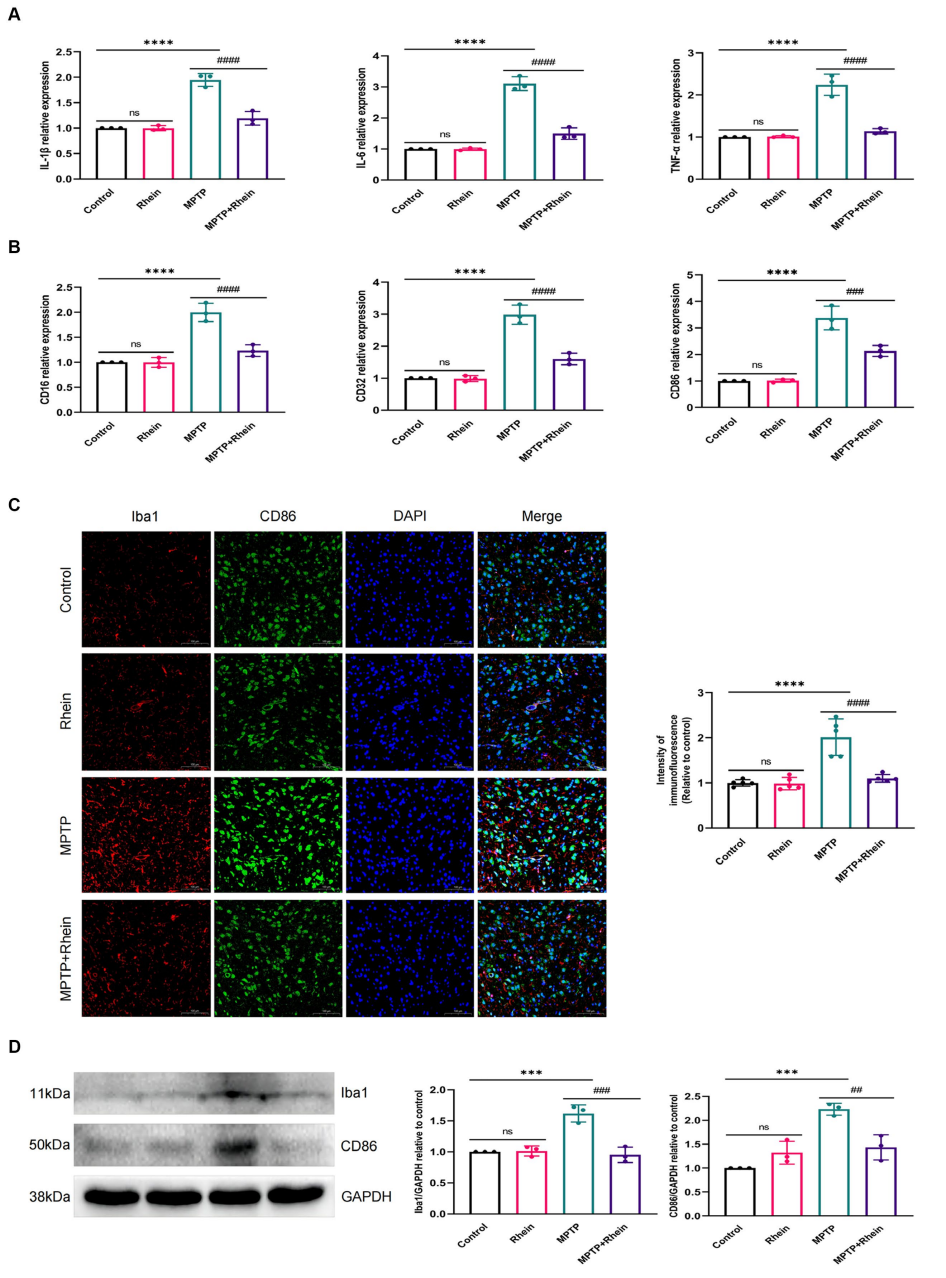
Rhein treatments alleviated MPTP-induced neuroinflammation in the SN of PD mice. **(A)** The expression of proinflammatory factors (IL-1β, IL-6, and TNF-α) in the SN as determined by RT-qPCR (*n* = 3). **(B)** The expression of M1 markers (CD16, CD32, and CD86) in the SN as determined by RT-qPCR (*n* = 3). **(C)** Immunofluorescence double staining for Iba1 (red) with CD86 (green) and quantitative analysis of the relative fluorescence intensity in the SN (*n* = 5). **(D)** Representative western blot bands and quantitative analysis of Iba1 and CD86 in the SN by normalization to GAPDH (*n* = 3). Data are presented as the mean ± SD. ***p < 0.001, ****p < 0.0001 compared with the control group; ##*p* < 0.01, ###*p* < 0.001, ####*p* < 0.0001 compared with the MPTP group.

**Figure 4:**
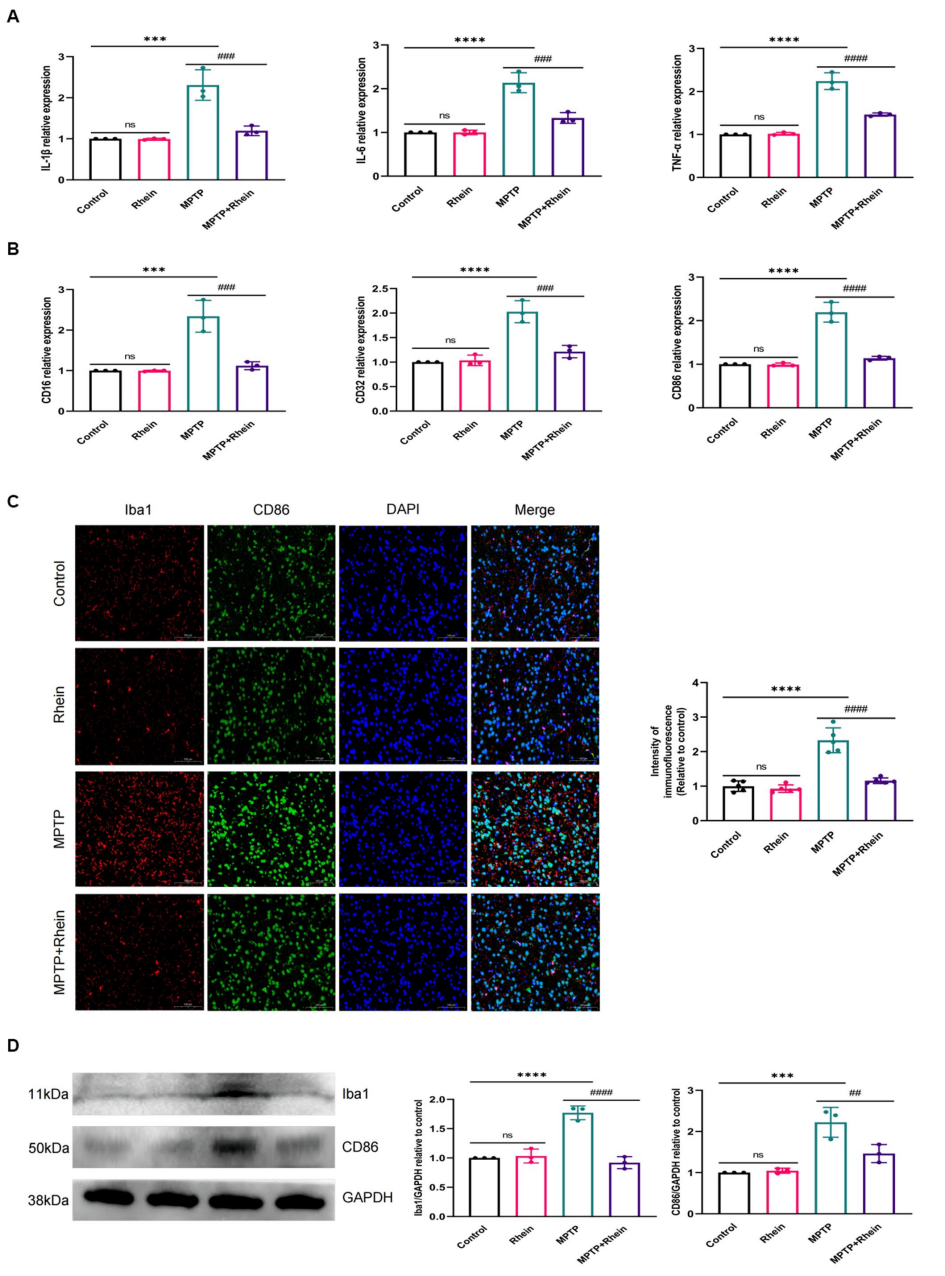
Rhein treatments alleviated MPTP-induced neuroinflammation in the striatum of PD mice. **(A)** The expression of proinflammatory factors (IL-1β, IL-6, and TNF-α) in the striatum as determined by RT-qPCR (*n* = 3). **(B)** The expression of M1 markers (CD16, CD32, and CD86) in the striatum as determined by RT-qPCR (*n* = 3). **(C)** Immunofluorescence double staining for Iba1 (red) with CD86 (green) and quantitative analysis of the relative fluorescence intensity in the striatum (*n* = 5). **(D)** Representative western blot bands and quantitative analysis of Iba1 and CD86 in the striatum by normalization to GAPDH (*n* = 3). Data are presented as the mean ± SD. ***p < 0.001, ****p < 0.0001 compared with the control group; ##*p* < 0.01, ###*p* < 0.001, ####*p* < 0.0001 compared with the MPTP group.

**Figure 5:**
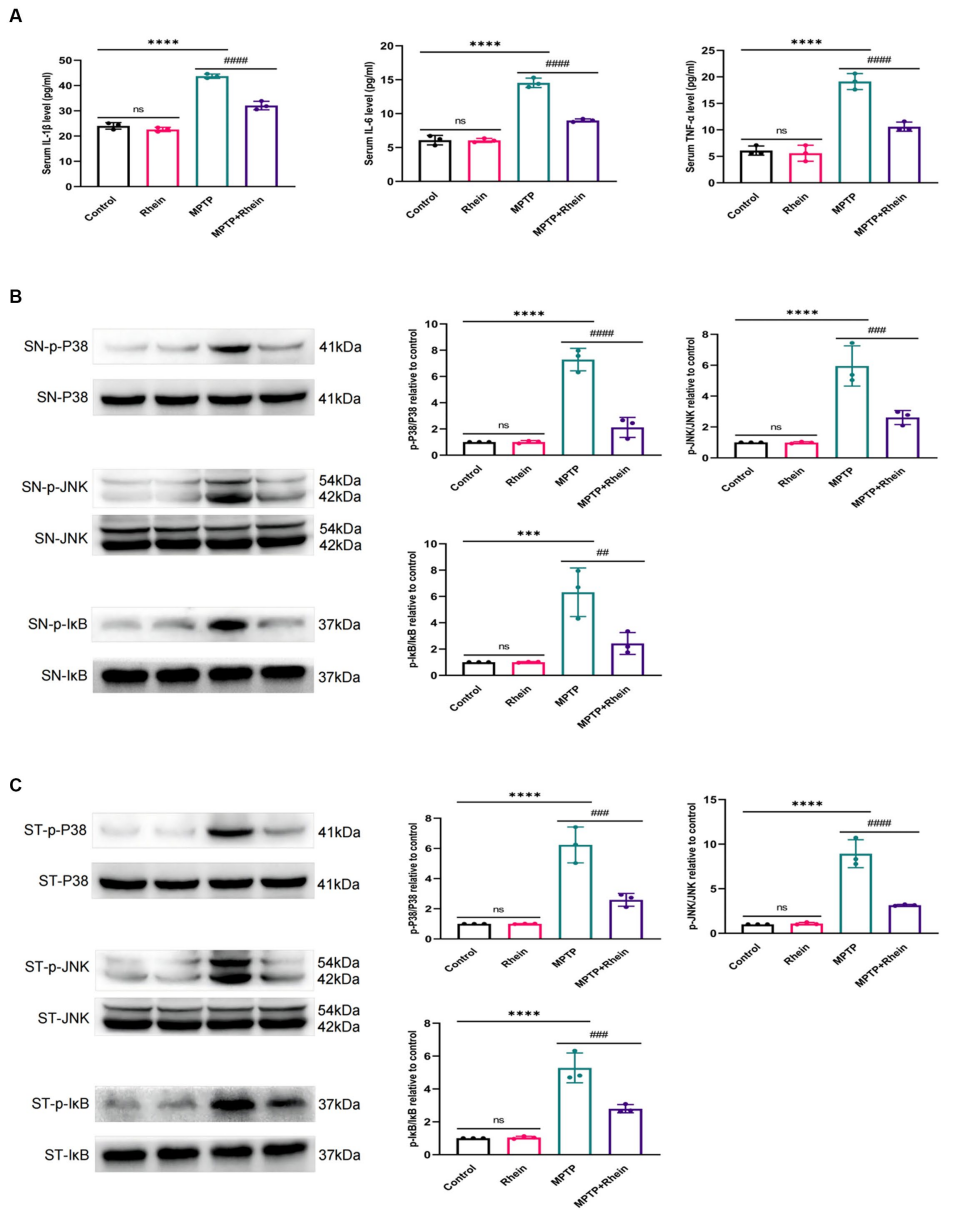
Rhein treatments inhibited the MPTP-induced inflammation and activation of MAPK/IκB signaling pathway in PD mice. **(A)** The levels of proinflammatory factors (IL-1β, IL-6, and TNF-α) in the serum of mice as determined by ELISA (*n* = 3). **(B)** Expression levels of p-P38, P38, p-JNK, JNK, p-IκB and IκB in the SN were analyzed by western blotting (*n* = 3). **(C)** Expression levels of p-P38, P38, p-JNK, JNK, p-IκB and IκB in the striatum were analyzed by western blotting (*n* = 3). Data are presented as the mean ± SD. ***p < 0.001, ****p < 0.0001 compared with the control group; ##*p* < 0.01, ###*p* < 0.001, ####*p* < 0.0001 compared with the MPTP group.

In neuroinflammation, cytokines and neurotoxic factors secreted by microglia cause neuronal damage and even death, among which TNF-α, IL-6 and IL-1β play a crucial part (Depino et al., 2005; Shastri et al., 2013; Olmos and Lladó, 2014; Lee et al., 2017). To determine whether rhein ameliorates MPTP-induced PD pathology in mice is associated with inhibition of inflammation, we measured proinflammatory cytokines (IL-1β, IL-6, and TNF-α) in substantia nigra, striatum, and serum. Levels of microglia activation markers (Iba1) and M1 polarization markers (CD16, CD32, and CD86) in substantia nigra and striatum were analyzed. The results showed that compared with the MPTP group, the levels of IL-1β, IL-6, and TNF-α in the substantia nigra, striatum, and serum were significantly decreased in the MPTP+Rhein group (Figures 3A, 4A, 5A). The expression levels of Iba1, CD16, CD32, and CD86 in the substantia nigra and striatum were also significantly decreased in the MPTP+Rhein group (Figures 3B-D, 4B-D). Apparently rhein treatment can inhibit inflammation and microglia M1 polarization in MPTP-induced PD mice *in vivo*.

### 3.4 Rhein treatment inhibited the activation of MAPK/IκB signaling pathway in MPTP-induced PD mice

To determine whether rhein treatment inhibited MPTP-induced activation of MAPK/IκB signaling pathway in PD mice, phosphorylation levels of MAPK/IκB signaling pathway proteins were detected by western blot. The results showed that compared with the MPTP group, the levels of p-P38,p-JNK and p-IκB in the substantia nigra and striatum were significantly decreased in the MPTP + Rhein group (Figures 5B,C). In conclusion, rhein treatment could inhibit the MAPK/IκB signaling pathway activation induced by MPTP in PD mice.

## 4 Discussion

*In vivo*, we evaluated the effects of rhein on neuroinflammation, pathology and motor deficits in MPTP-induced PD mice, as well as related mechanism. We demonstrated that rhein alleviated neuroinflammation, dyskinesia, neurodegeneration and α-synuclein deposition in MPTP-induced PD mice.

It has been confirmed that MAPK/NFκB signaling pathway is involved in the regulation of neuroinflammation in several studies (Xu et al., 2018; Zhang et al., 2019; Li et al., 2020; Zheng et al., 2020). Moreover, MAPK/NFκB signaling pathway strongly linked to many CNS diseases for example PD, AD and EP, playing an important role in the protection of nerves and improvement of disease symptoms (Kam et al., 2020; Li et al., 2020; Singh and Singh, 2020; Sun et al., 2022). To investigate the effects of rhein on neuroinflammation and neurons, we first explored the effects of rhein on MAPK/IκB signaling pathway. Our results showed that rhein inhibited the activation of MAPK/IκB signaling pathway in MPTP-induced PD mice. That meant rhein may have a good therapeutic effect on neuroinflammation and damaged neurons of MPTP-induced PD mice.

To determine whether rhein can alleviate neuroinflammation by inhibiting MAPK/IκB signaling pathway, MPTP was used to induce neuroinflammation in mice (Li et al., 2020). It has been reported that MPTP induces microglia M1 polarization and proinflammatory cytokines (TNF-α, IL-1β, and IL-6) production (Yan et al., 2018). Our results showed that rhein treatment indeed inhibited M1 polarization and proinflammatory cytokine production in MPTP-induced PD mice, effectively reducing neuroinflammation.

The proinflammatory cytokines produced by overactivated microglia can damage neurons; that is, neuroinflammation may cause neuronal death. Studies have found that MAPK/NFκB signaling pathway is important for protecting neurons (Li et al., 2020; Yu et al., 2021). In addition, our results have shown that rhein inhibited neuroinflammation by regulating the MAPK/IκB signaling pathway. Therefore, we hypothesized that rhein may play a neuroprotective role by reducing neuroinflammation via MAPK/IκB signaling pathway. Our previous study showed that rhein could increase the cell viability and decrease the apoptosis rate of SH-SY5Y cells by reducing the neuroinflammation, demonstrating the neuroprotective ability of rhein *in vitro*. Our further *in vivo* studies found that rhein ameliorated neurodegeneration, motor deficits and α-synuclein deposition in PD mice by inhibiting neuroinflammation through MAPK/IκB signaling pathway.

In this study, we found that rhein alleviated MPTP-induced neurodegeneration in PD mouse model. In the future, we can further verify the role of rhein in other PD models, comprehensively explore the mechanism of rhein protecting neurons and improving PD, and explore the potential of rhein in neurodegenerative diseases.

## 5 Conclusion

Our study found that rhein alleviated motor deficits, neurodegeneration and α-synuclein deposition in MPTP-induced PD mice by inhibiting neuroinflammation through MAPK/IκB signaling pathway. This finding contributed to the understanding of the mechanism by which rhein protected neurons and may provide ideas for the treatment of PD.

## Data availability statement

The raw data supporting the conclusions of this article will be made available by the authors, without undue reservation.

## Ethics statement

The animal study was approved by Experimental Animal Welfare Ethics Committee of Nanchang University. The study was conducted in accordance with the local legislation and institutional requirements.

## Author contributions

XQ: Writing – original draft, Writing – review & editing. SW: Methodology, Writing – review & editing. JH: Methodology, Writing – review & editing. BH: Methodology, Writing – review & editing. XY: Formal analysis, Writing – review & editing. LL: Formal analysis, Writing – review & editing. RZ: Writing – review & editing, Methodology. WH: Writing – review & editing, Funding acquisition, Project administration.
