# Supplementary figures and images for: Rhein alleviates MPTP-induced Parkinson’s disease by suppressing neuroinflammation via MAPK/IκB pathway
Source: Front Neurosci. 2024 Jun 12;18:1396345. doi: 10.3389/fnins.2024.1396345 (PMC11202316; doi:10.3389/fnins.2024.1396345)

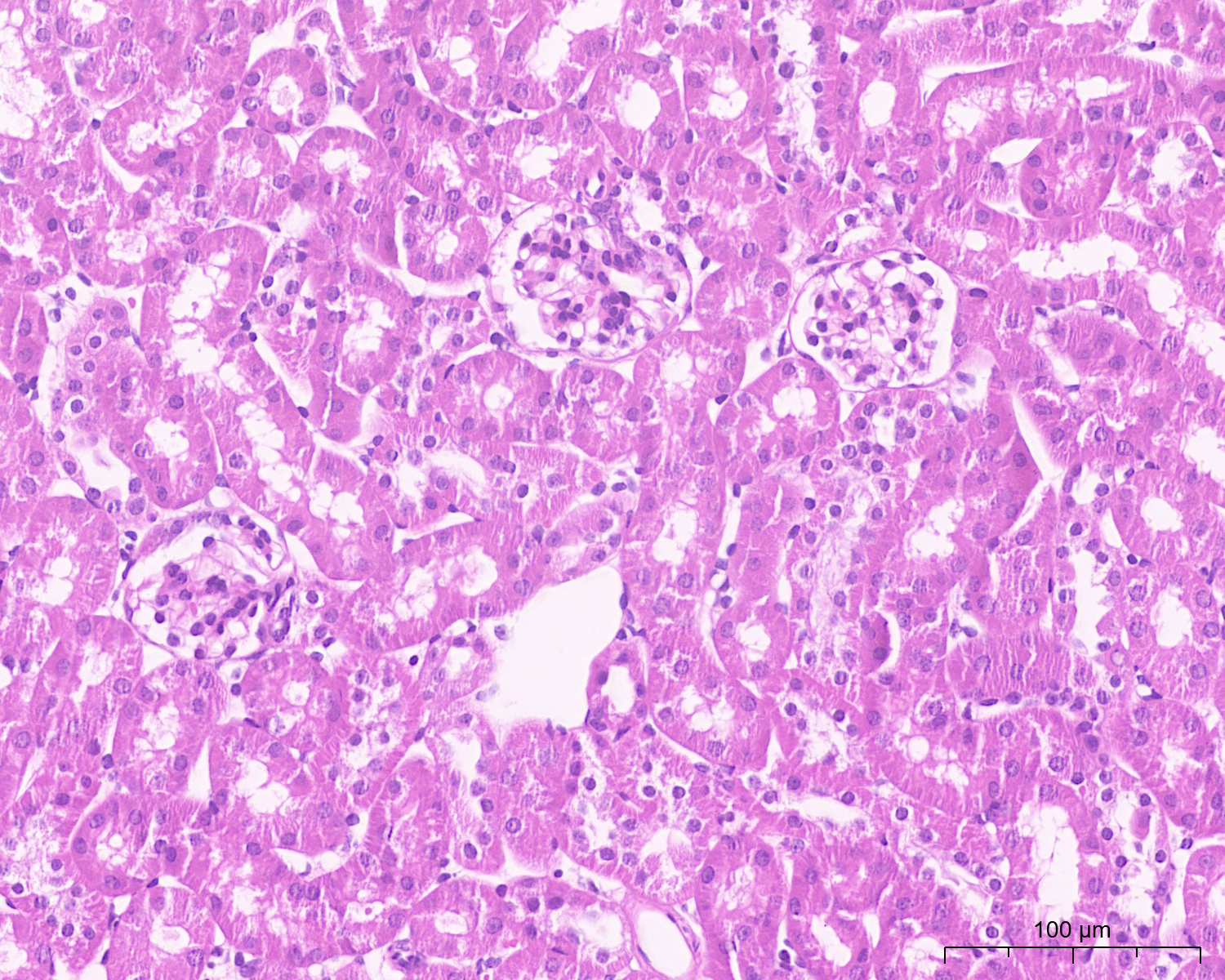

Supplement: Supplementary file 1 [file Data_Sheet_1.ZIP › HE/kidney/59C╔÷_40.0x.tif]

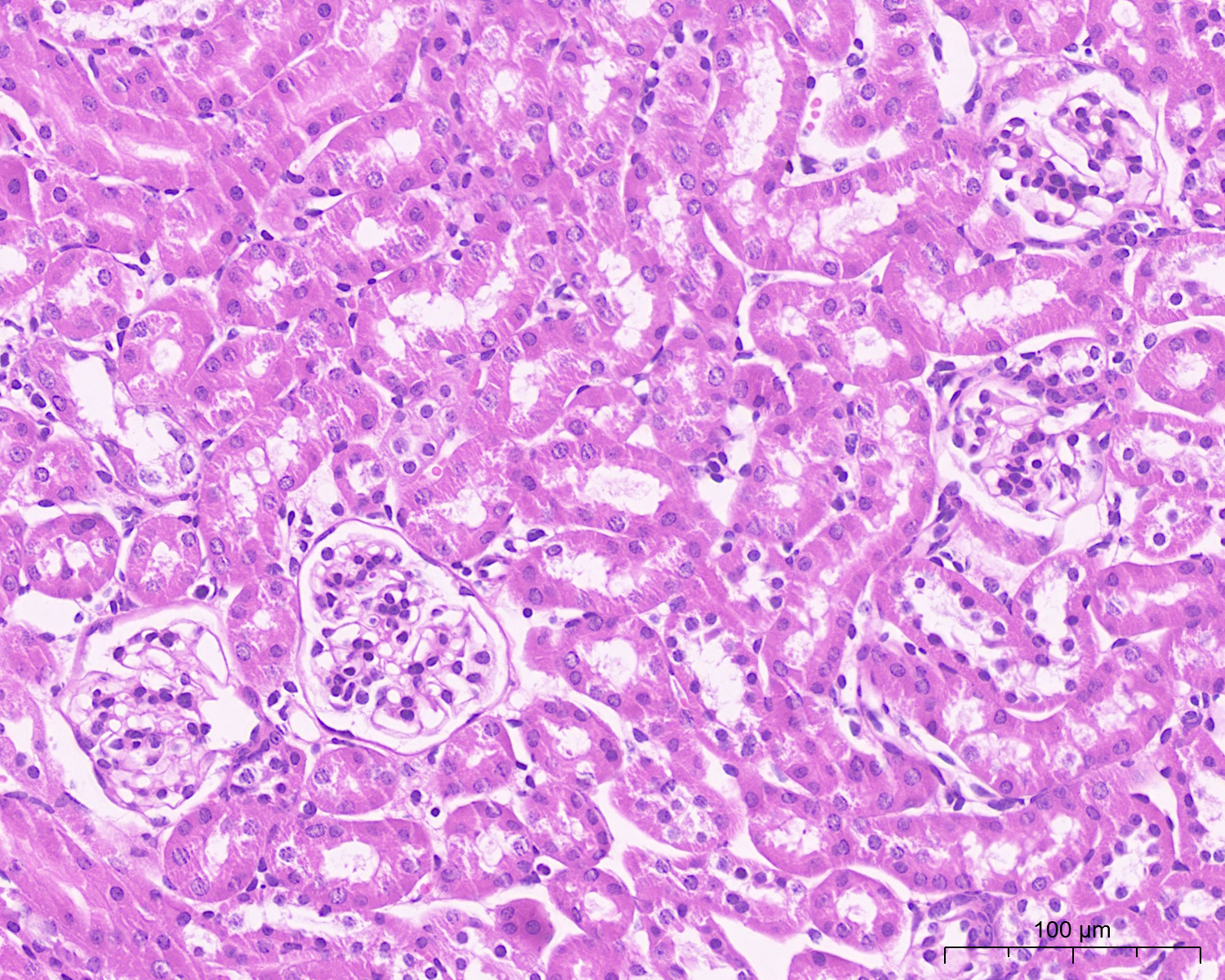

Supplement: Supplementary file 1 [file Data_Sheet_1.ZIP › HE/kidney/79R╔÷_40.0x.tif]

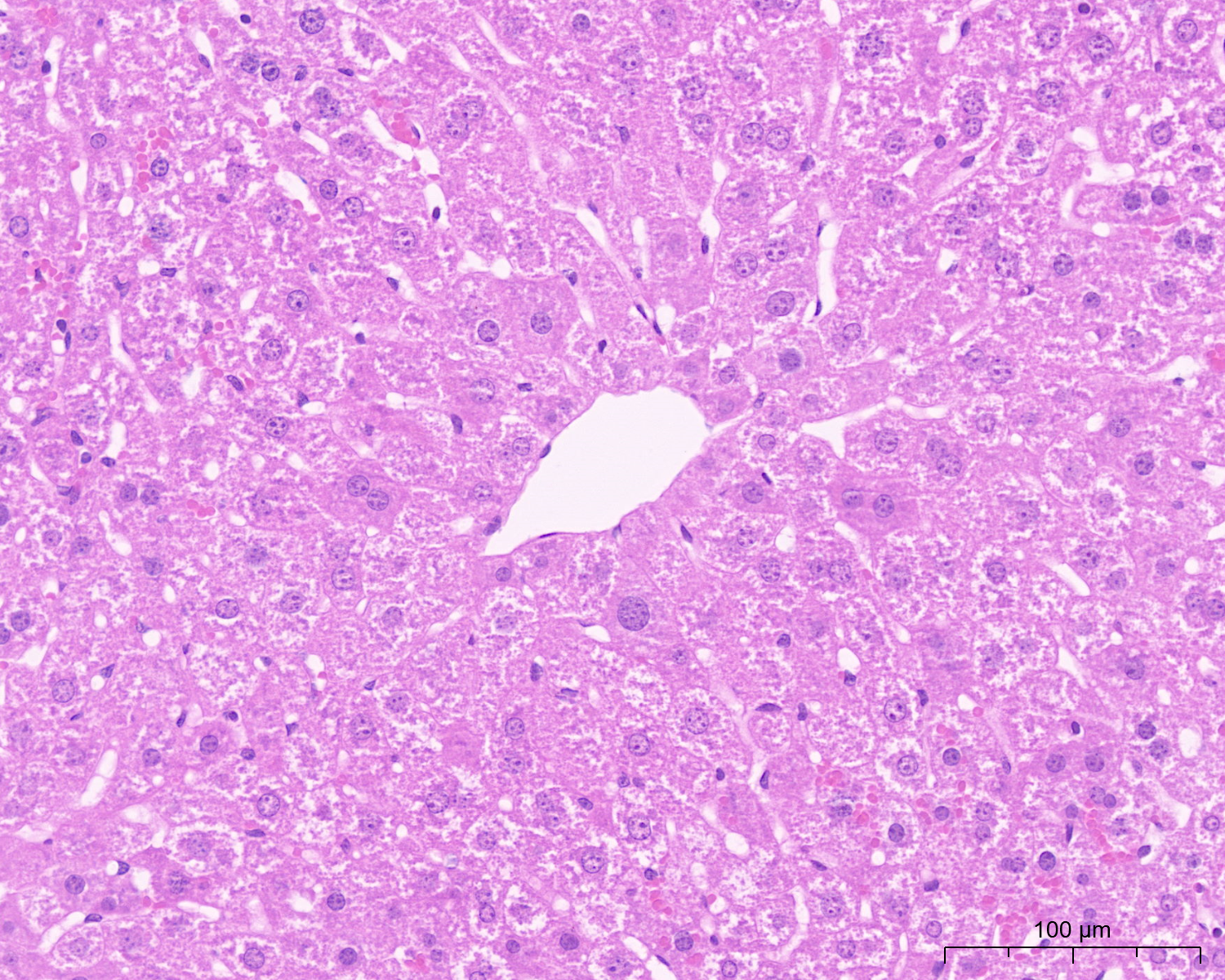

Supplement: Supplementary file 1 [file Data_Sheet_1.ZIP › HE/liver/58R╕╬_40.0x.tif]

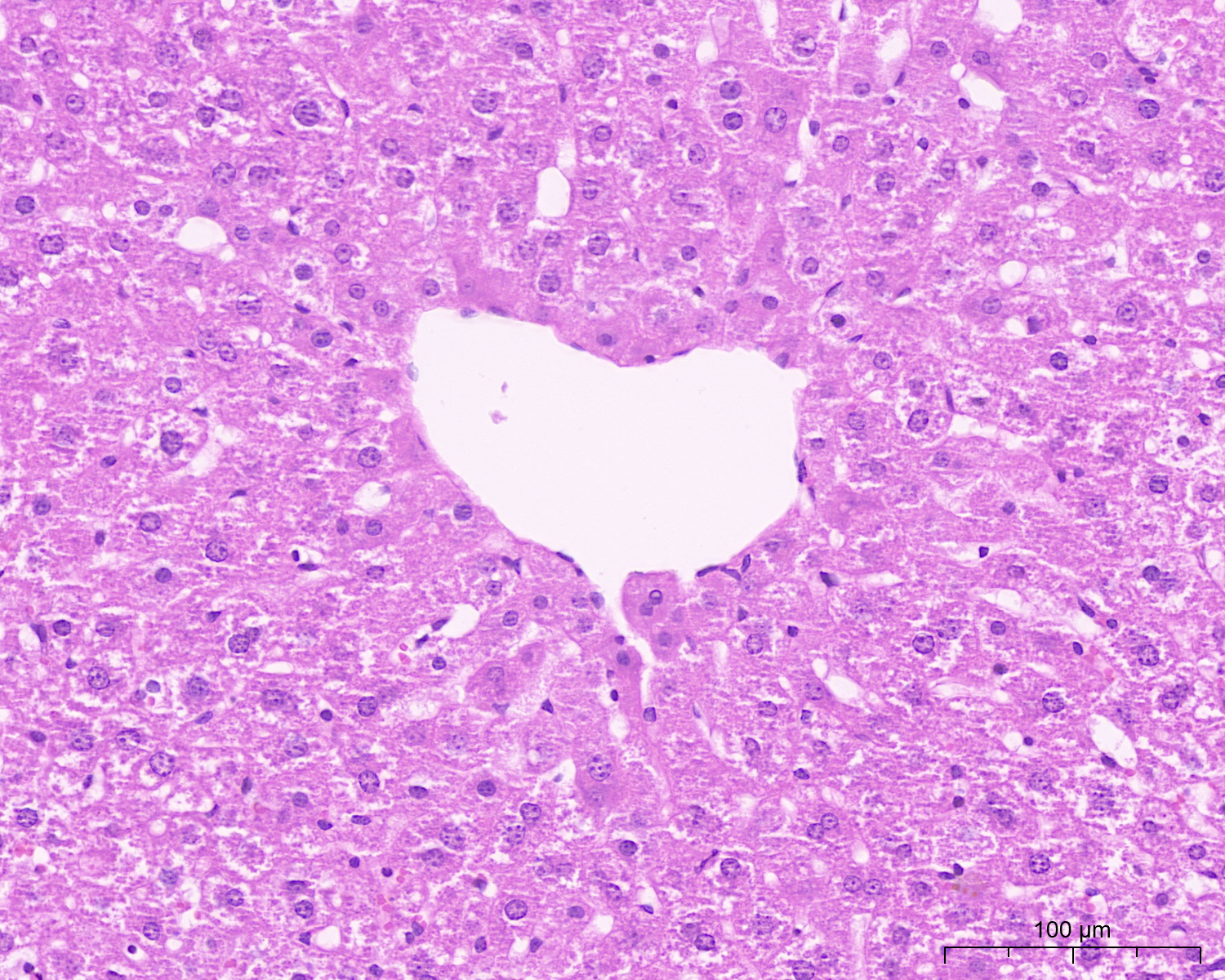

Supplement: Supplementary file 1 [file Data_Sheet_1.ZIP › HE/liver/78C╕╬_40.0x.tif]

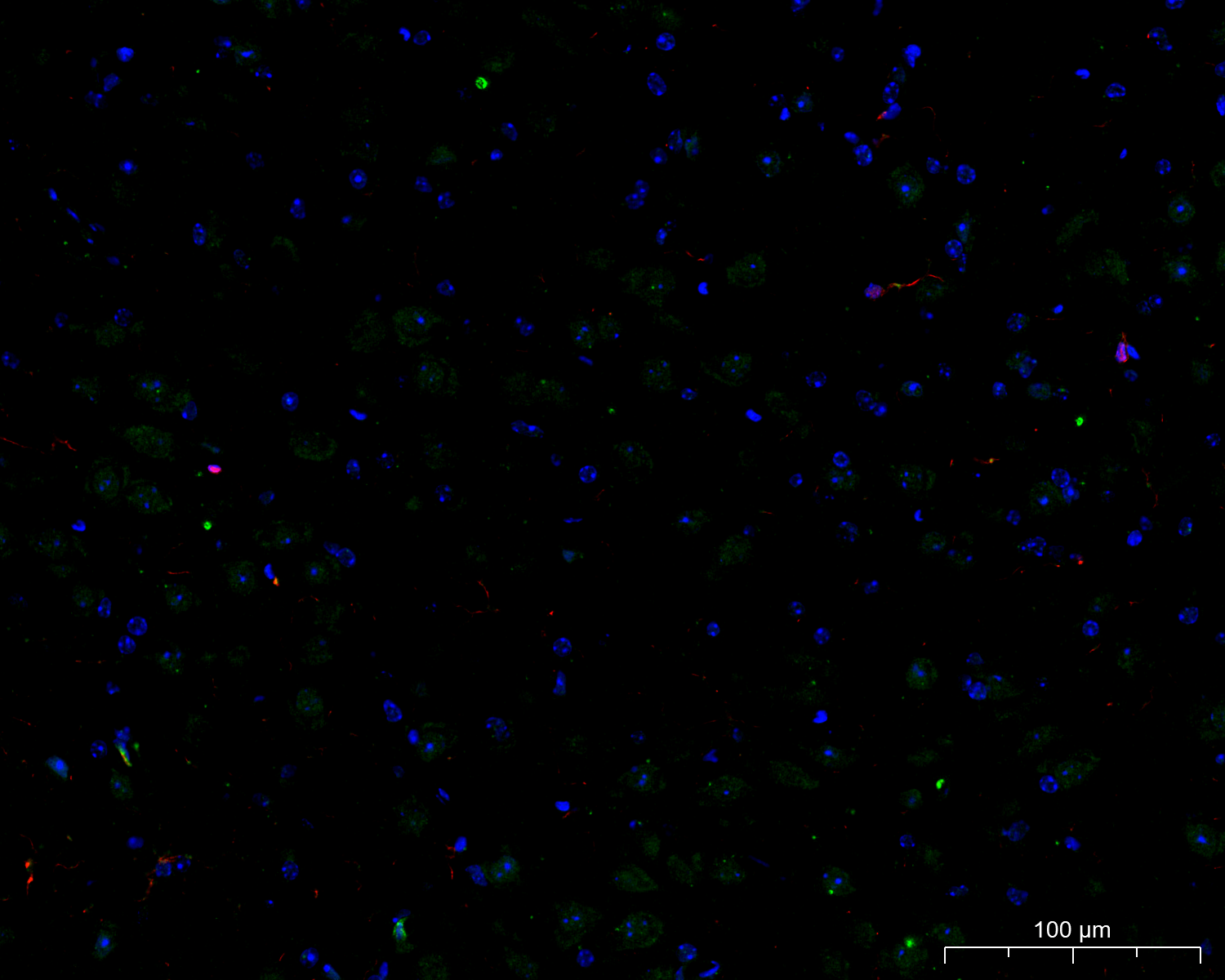

Supplement: Supplementary file 2 [file Data_Sheet_2.ZIP › IF/SN/C/69C║┌╓╩ IF IBA-1║∞+CD86┬╠_40.0x.tif]

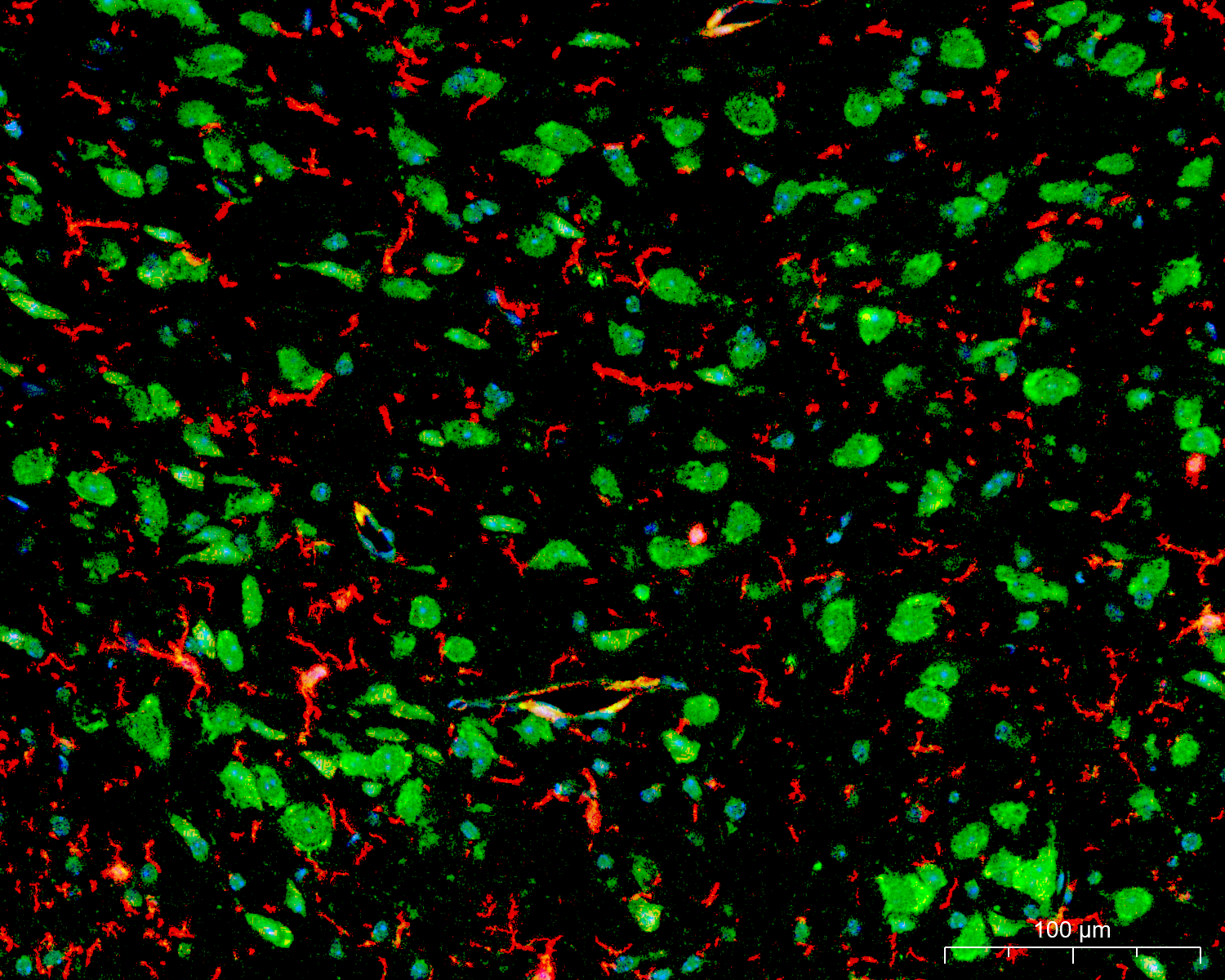

Supplement: Supplementary file 2 [file Data_Sheet_2.ZIP › IF/SN/M/21M║┌╓╩ IF IBA-1║∞+CD86┬╠_40.0x.tif]

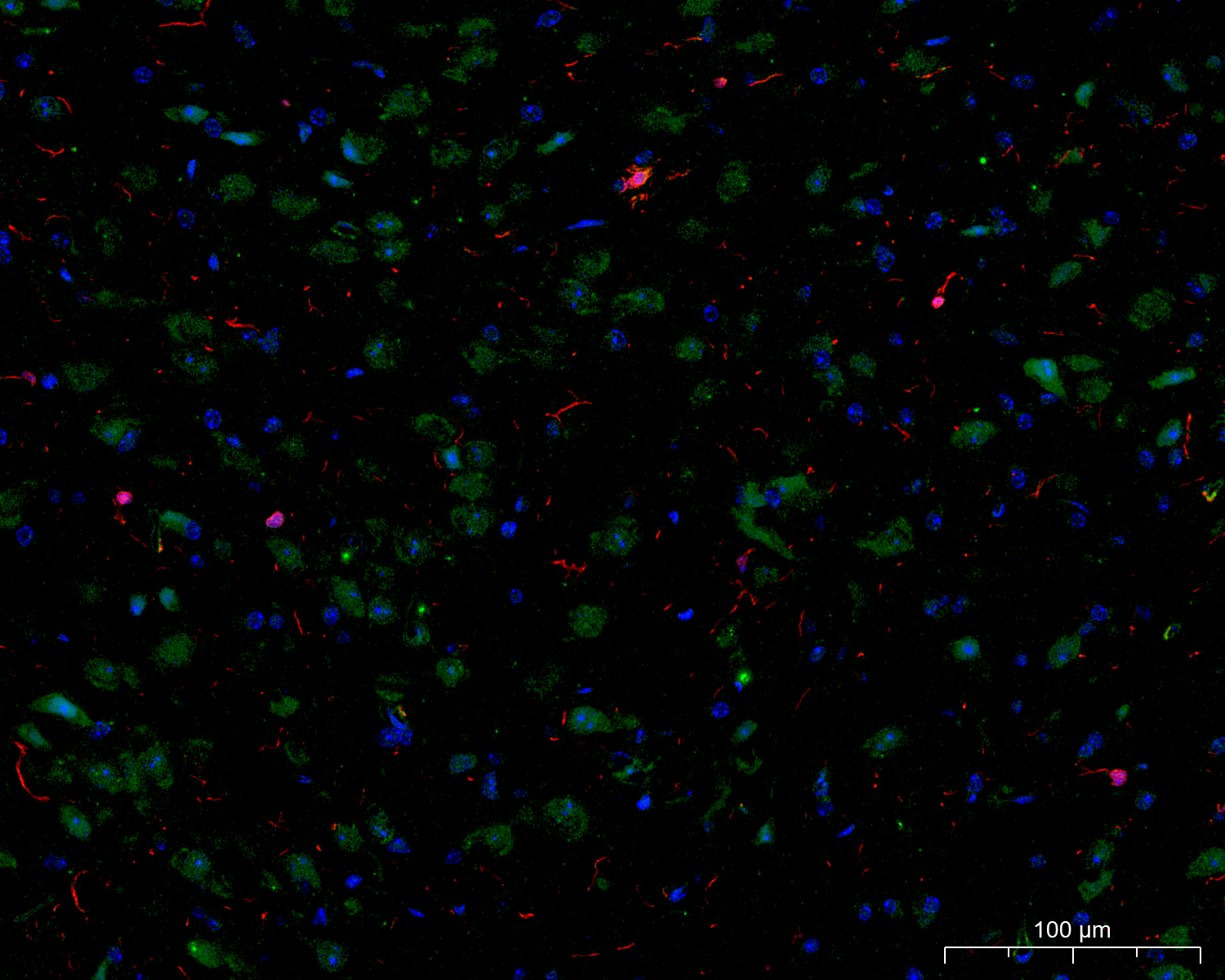

Supplement: Supplementary file 2 [file Data_Sheet_2.ZIP › IF/SN/MR/50MR║┌╓╩ IF IBA-1║∞+CD86┬╠_40.0x.tif]

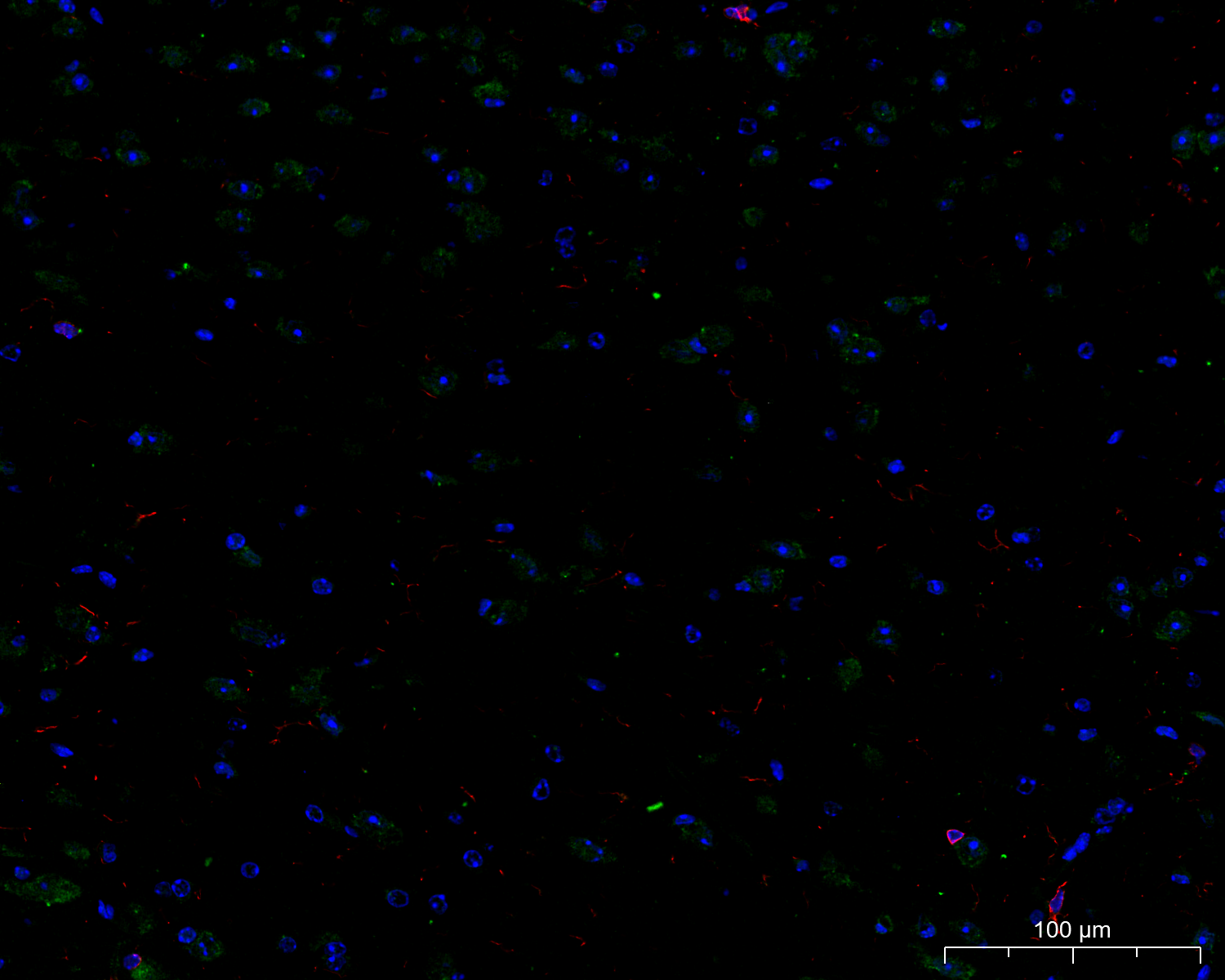

Supplement: Supplementary file 2 [file Data_Sheet_2.ZIP › IF/SN/R/62R║┌╓╩ IF IBA-1║∞+CD86┬╠_40.0x.tif]

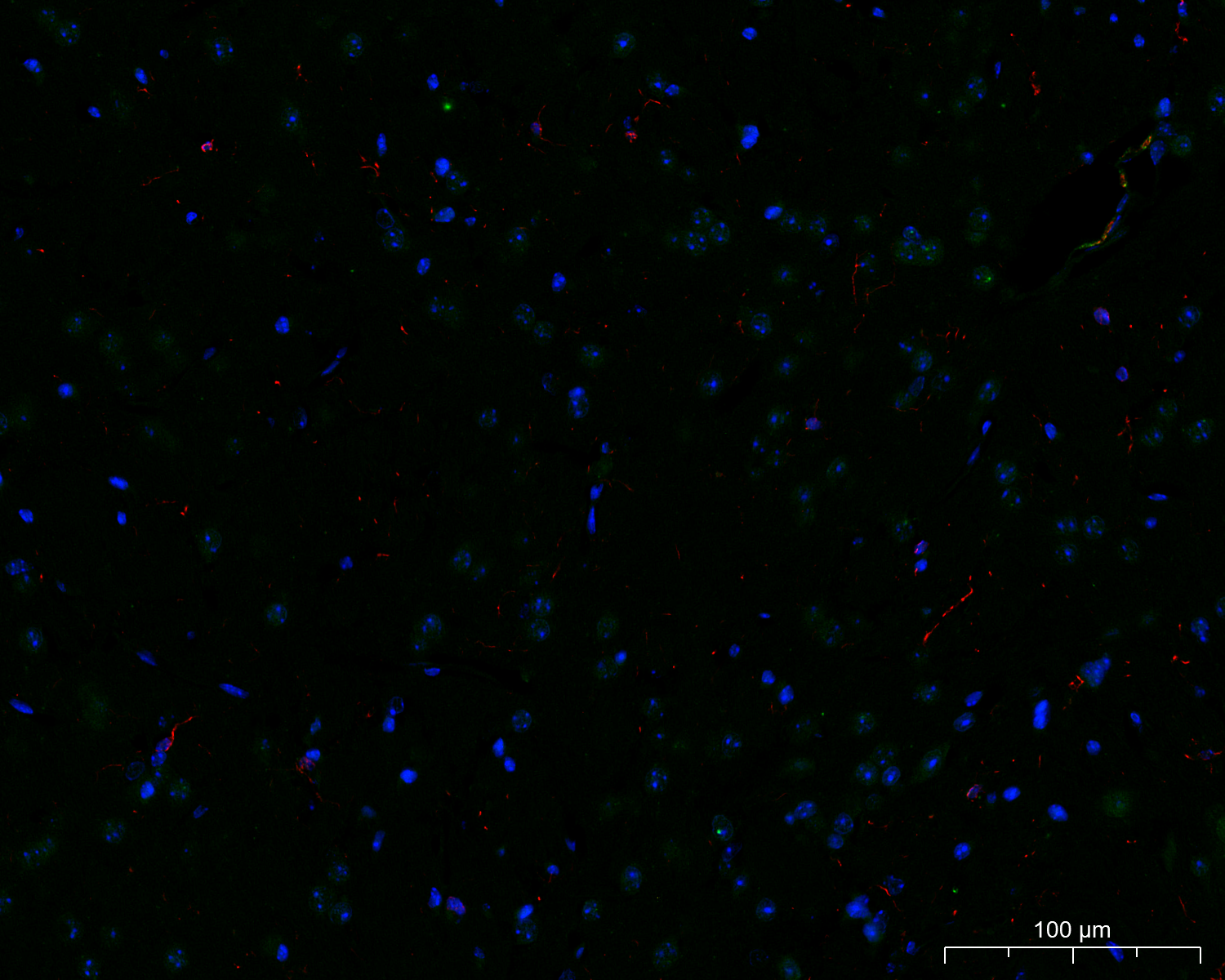

Supplement: Supplementary file 2 [file Data_Sheet_2.ZIP › IF/ST/C/83C╬╞╫┤╠σ IF IBA-1║∞+CD86┬╠_40.0x.tif]

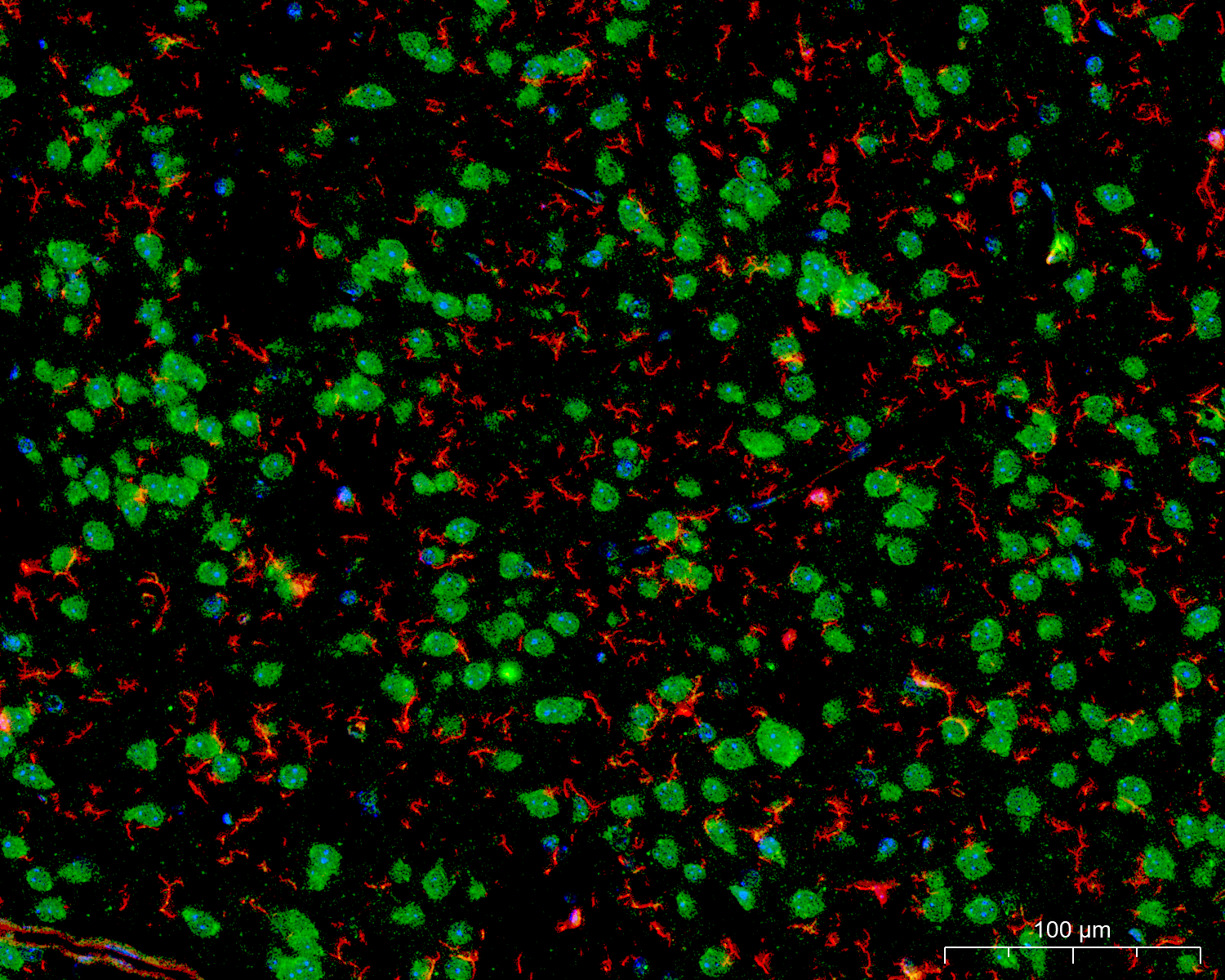

Supplement: Supplementary file 2 [file Data_Sheet_2.ZIP › IF/ST/M/27M╬╞╫┤╠σ IF IBA-1║∞+CD86┬╠_40.0x.tif]

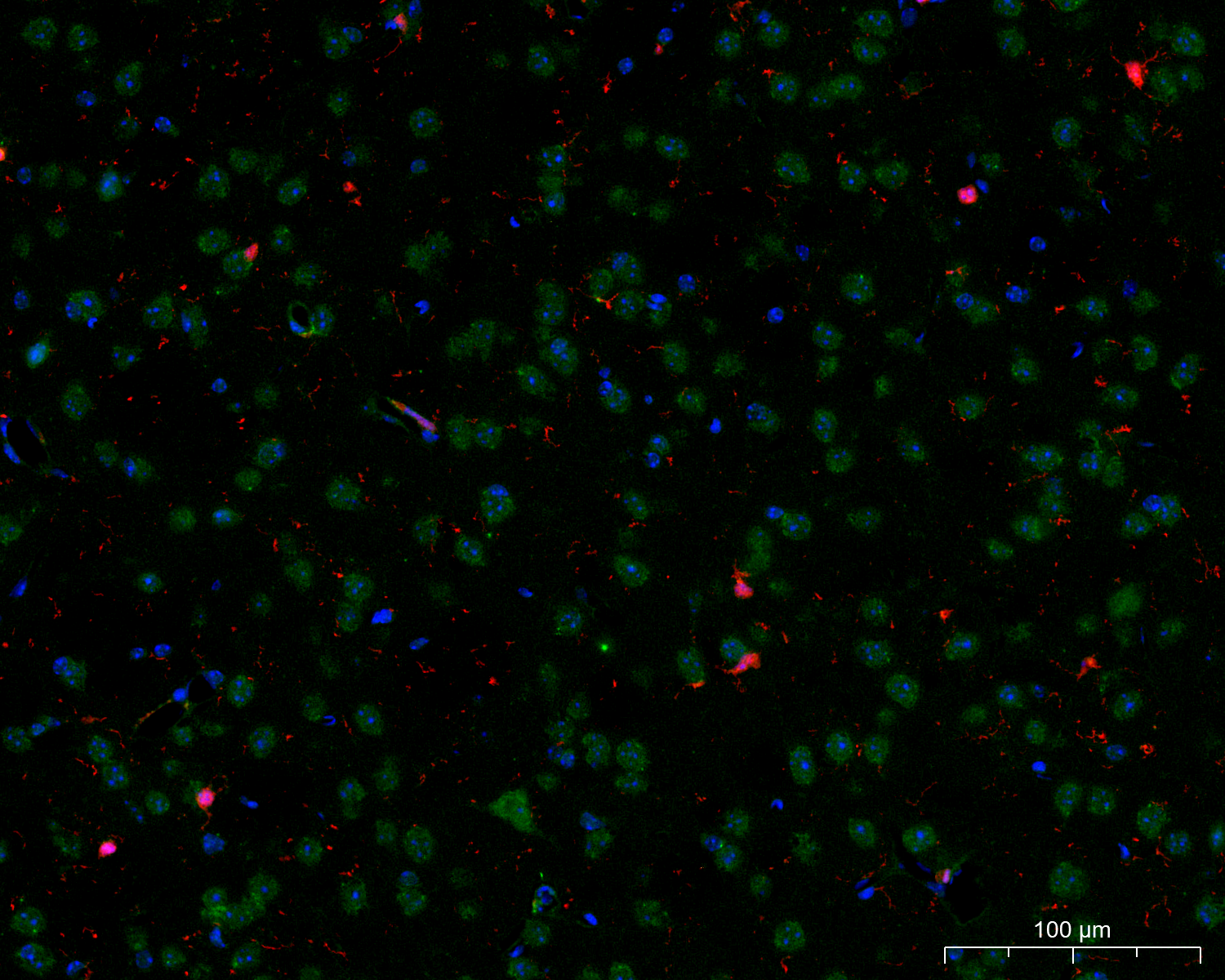

Supplement: Supplementary file 2 [file Data_Sheet_2.ZIP › IF/ST/MR/53MR╬╞╫┤╠σ IF IBA-1║∞+CD86┬╠_40.0x.tif]

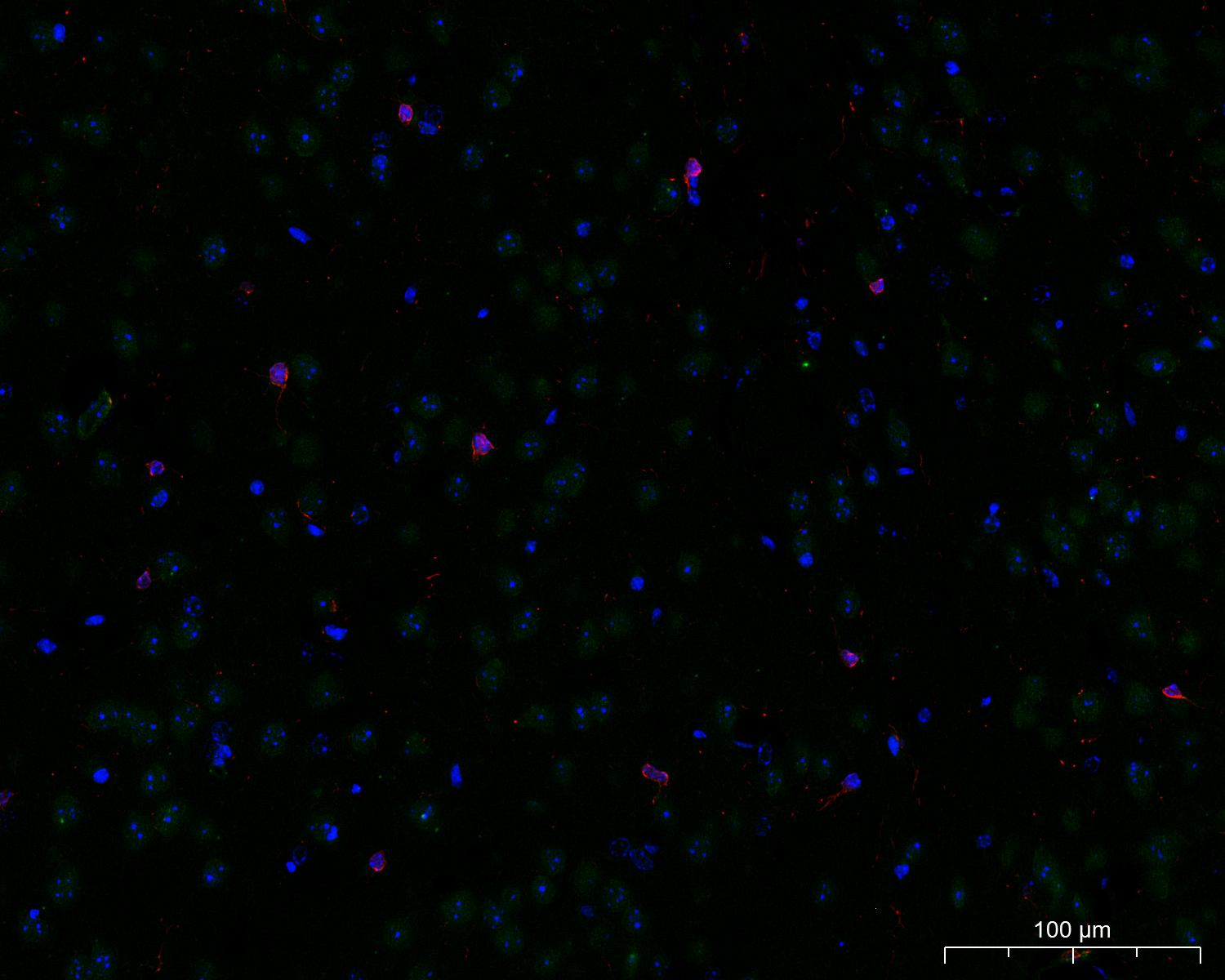

Supplement: Supplementary file 2 [file Data_Sheet_2.ZIP › IF/ST/R/62R╬╞╫┤╠σ IF IBA-1║∞+CD86┬╠_40.0x.tif]

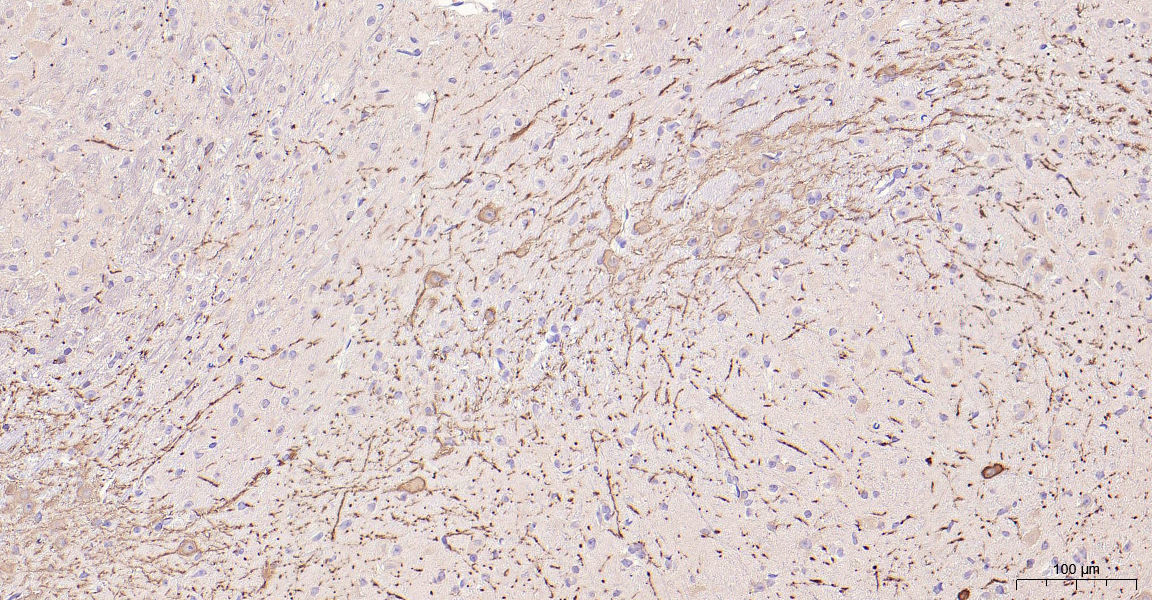

Supplement: Supplementary file 3 [file Data_Sheet_3.ZIP › IHC/SN/36M║┌╓╩ TH_20.0x.tif]

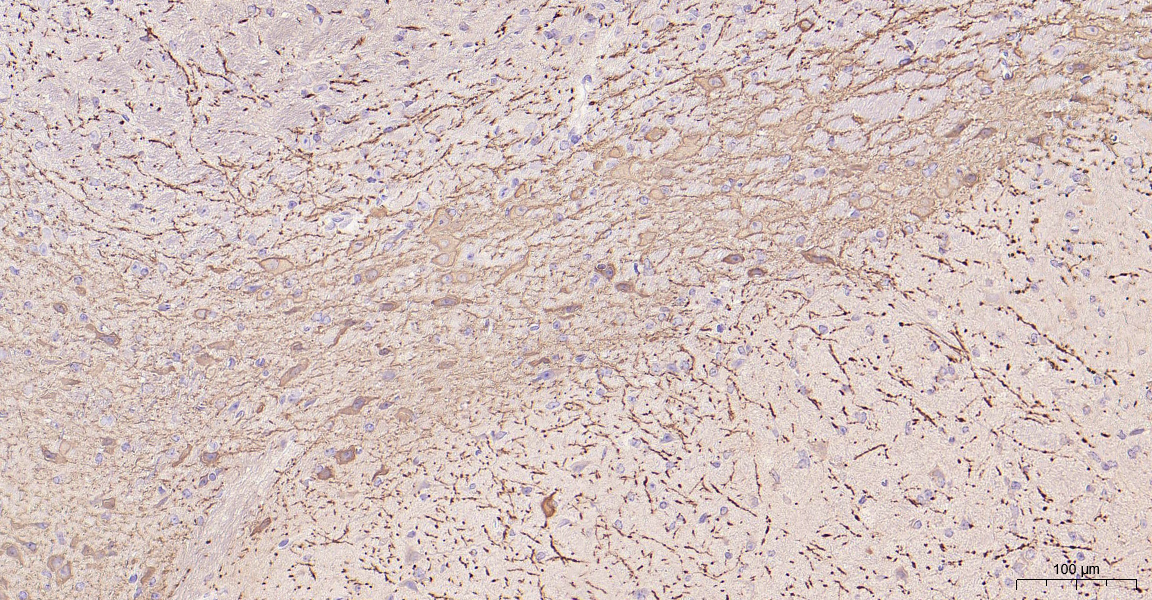

Supplement: Supplementary file 3 [file Data_Sheet_3.ZIP › IHC/SN/50MR║┌╓╩ TH_20.0x.tif]

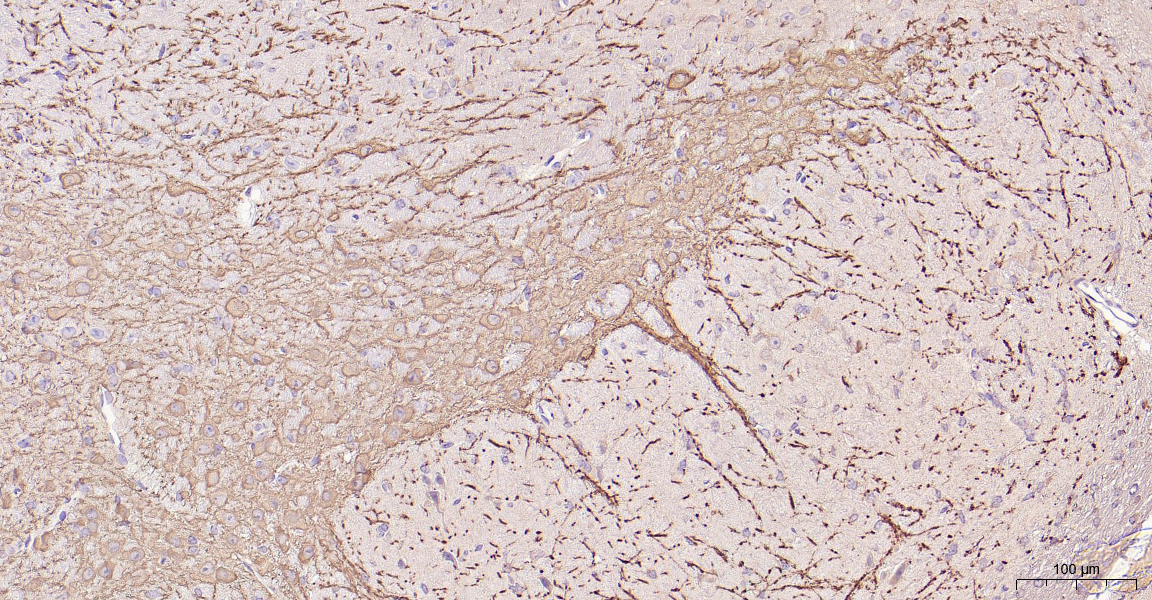

Supplement: Supplementary file 3 [file Data_Sheet_3.ZIP › IHC/SN/57C║┌╓╩ TH_20.0x.tif]

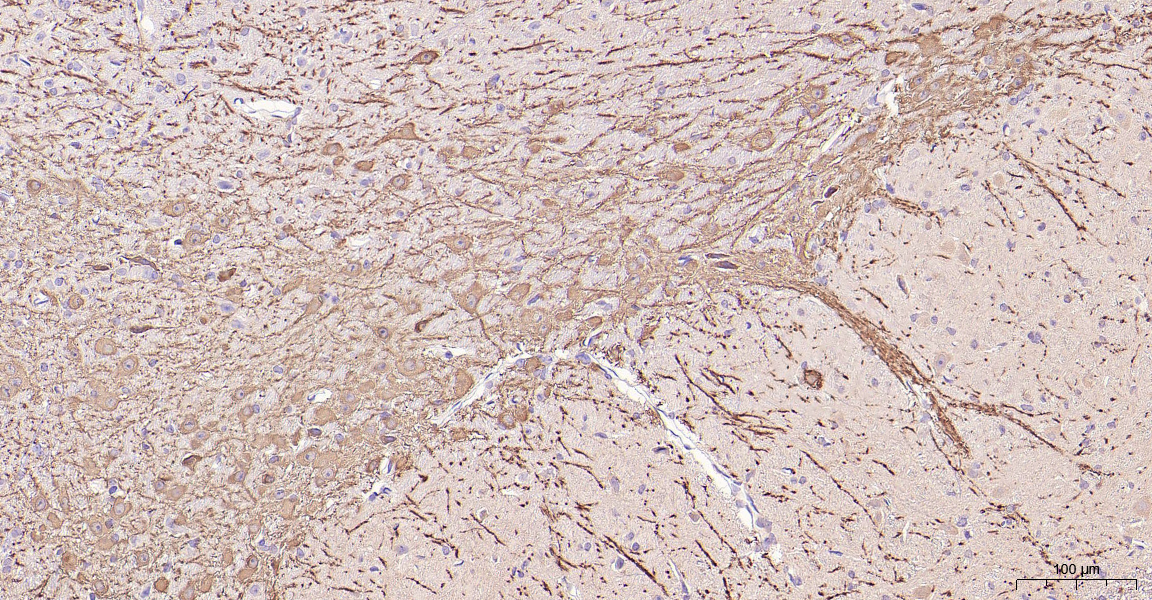

Supplement: Supplementary file 3 [file Data_Sheet_3.ZIP › IHC/SN/58R║┌╓╩ TH_20.0x.tif]

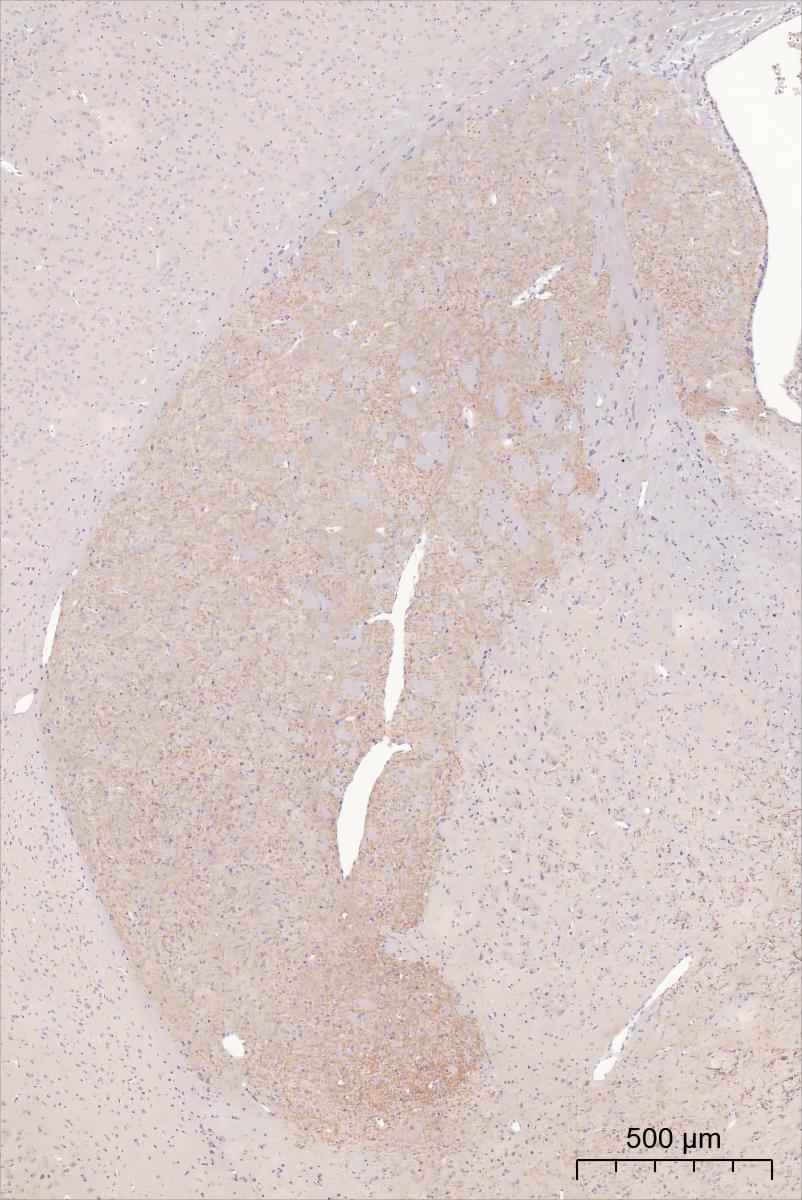

Supplement: Supplementary file 3 [file Data_Sheet_3.ZIP › IHC/ST/29M╬╞╫┤╠σ TH_5.0x(1).jpg]

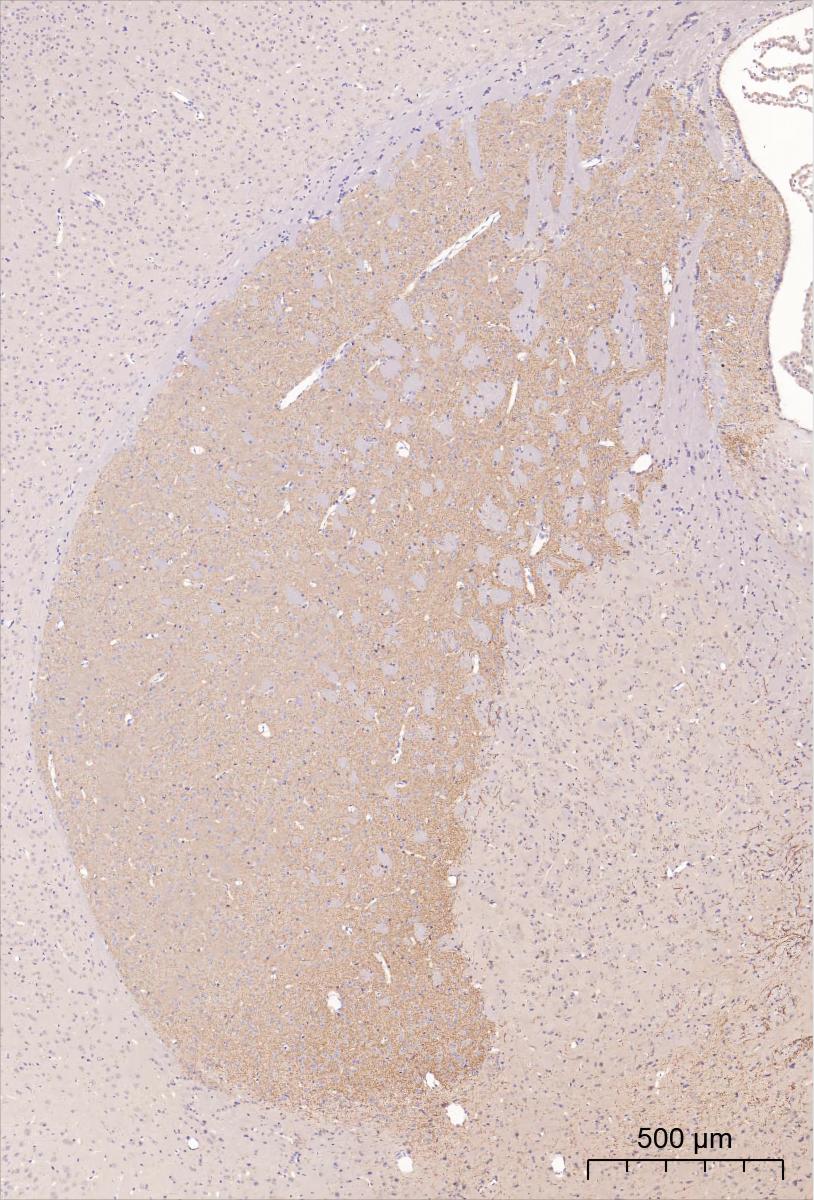

Supplement: Supplementary file 3 [file Data_Sheet_3.ZIP › IHC/ST/37R╬╞╫┤╠σ TH_5.0x(1).jpg]

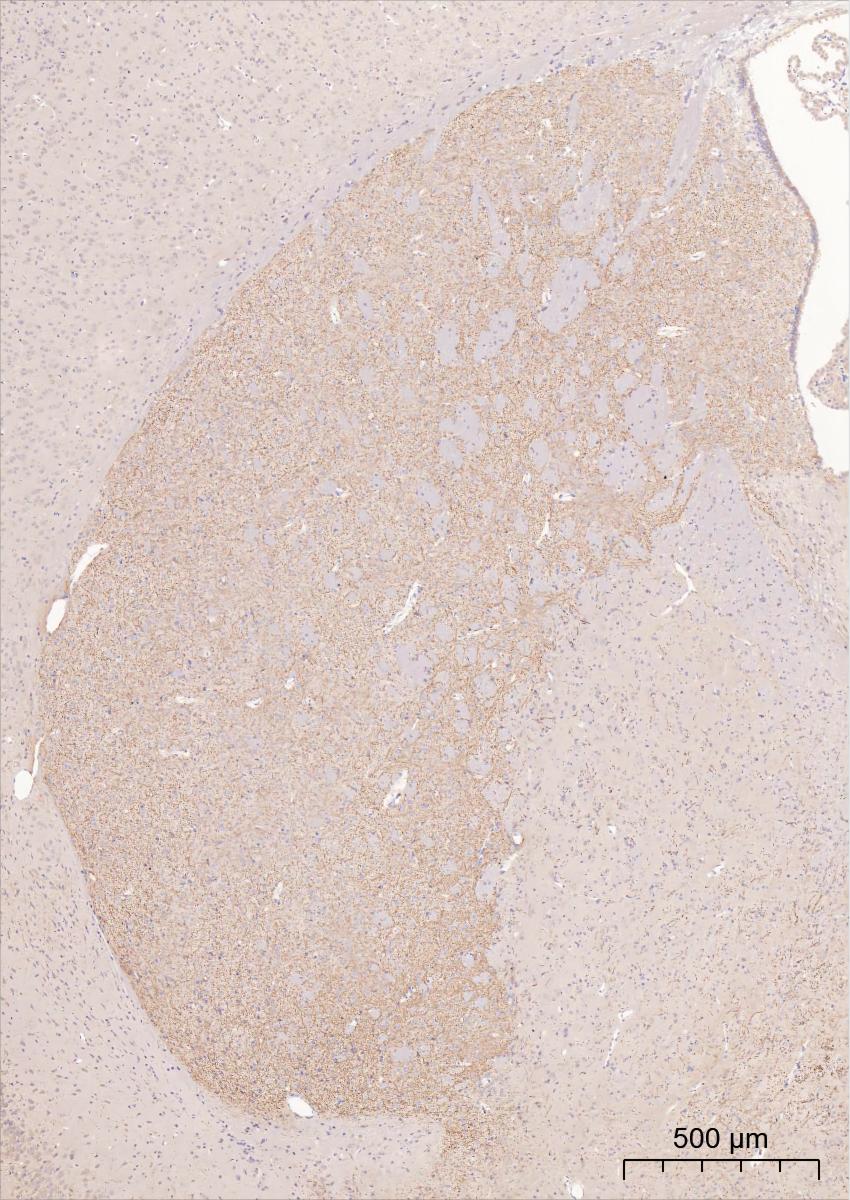

Supplement: Supplementary file 3 [file Data_Sheet_3.ZIP › IHC/ST/53MR╬╞╫┤╠σ TH_5.0x(1).jpg]

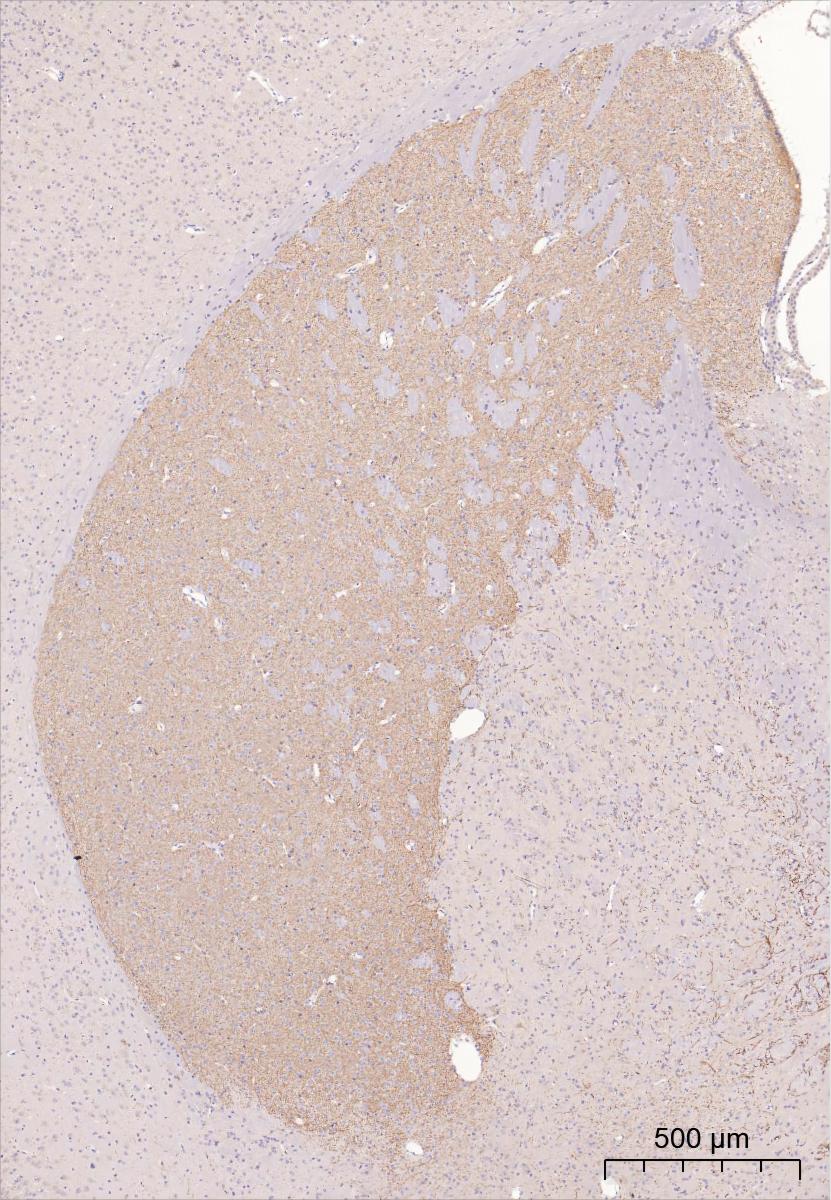

Supplement: Supplementary file 3 [file Data_Sheet_3.ZIP › IHC/ST/69C╬╞╫┤╠σ TH_5.0x(1).jpg]

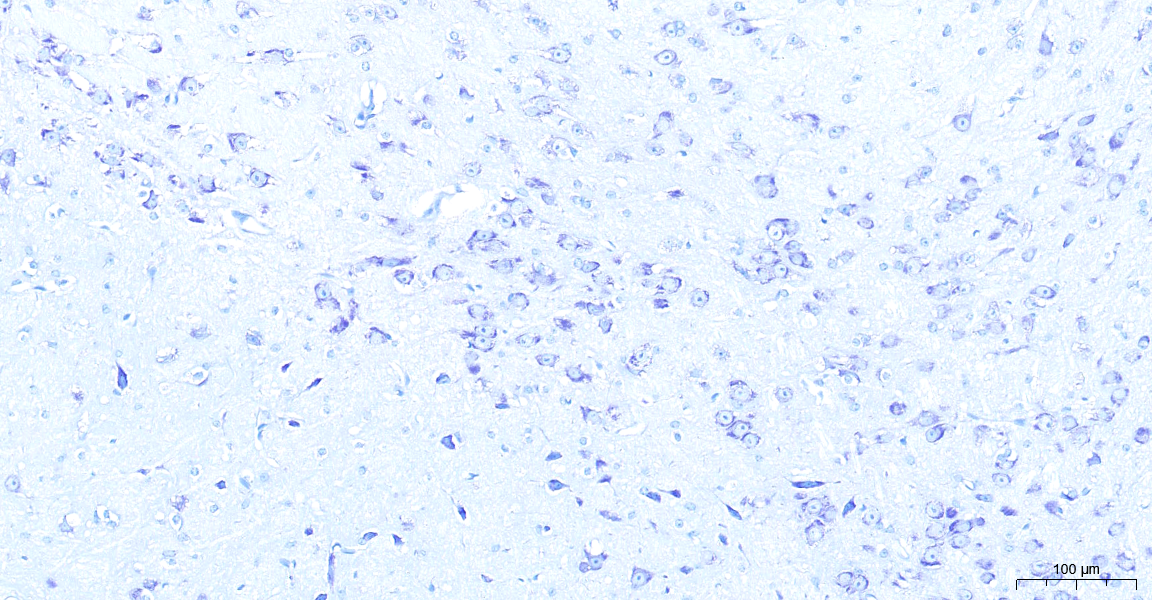

Supplement: Supplementary file 4 [file Data_Sheet_4.ZIP › Nissl's staining/26M║┌╓╩ ─ß╩╧_20.0x.tif]

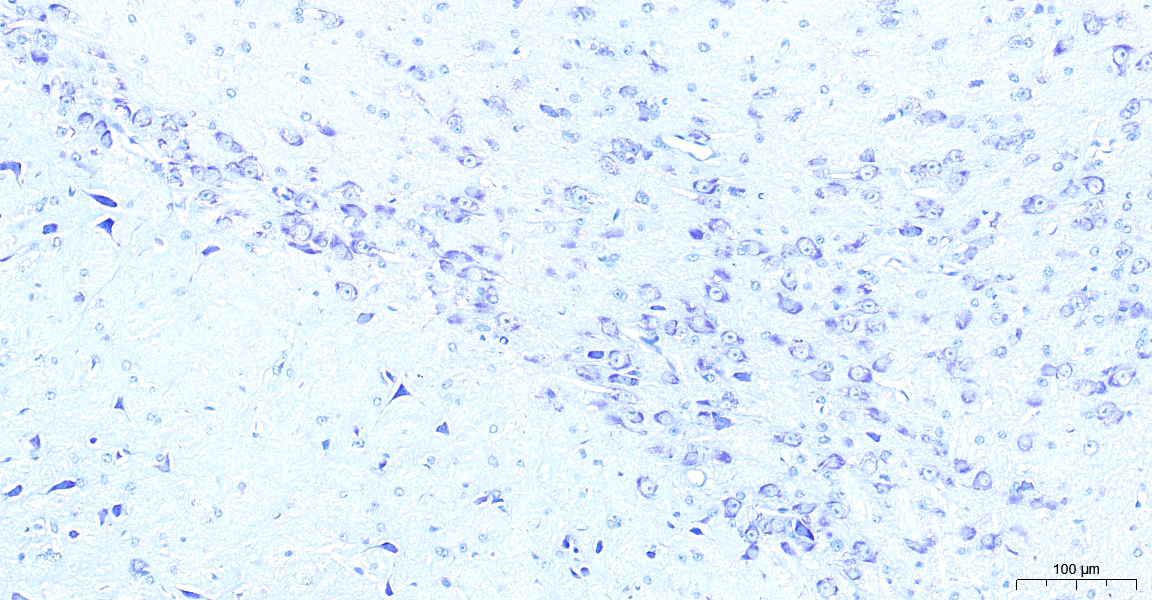

Supplement: Supplementary file 4 [file Data_Sheet_4.ZIP › Nissl's staining/50mr║┌╓╩ ─ß╩╧_20.0x.tif]

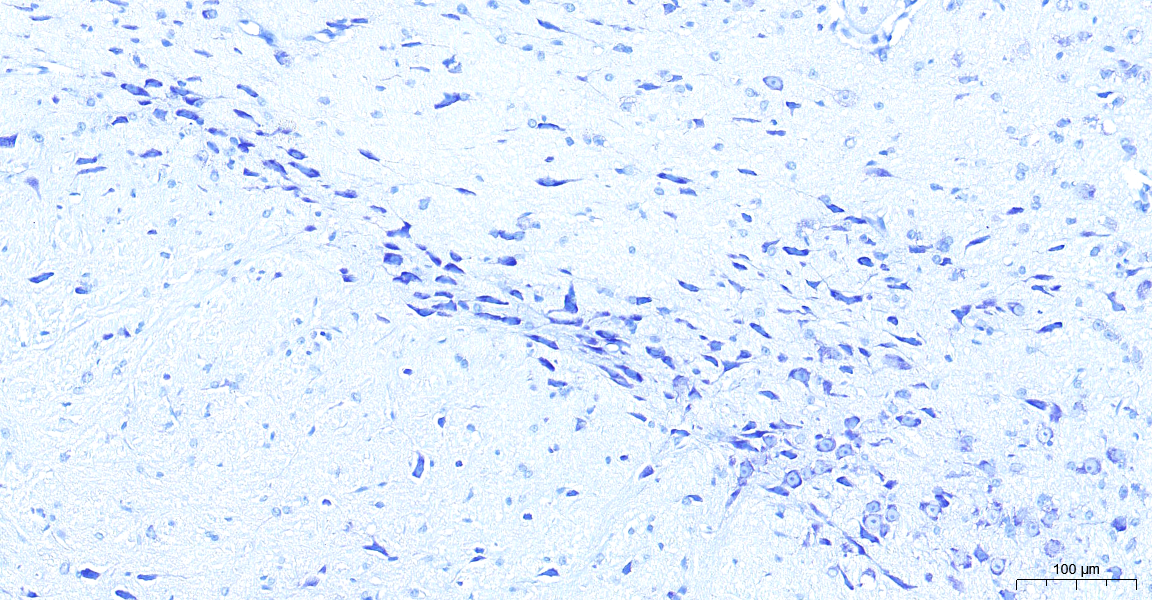

Supplement: Supplementary file 4 [file Data_Sheet_4.ZIP › Nissl's staining/57c║┌╓╩ ─ß╩╧_20.0x.tif]

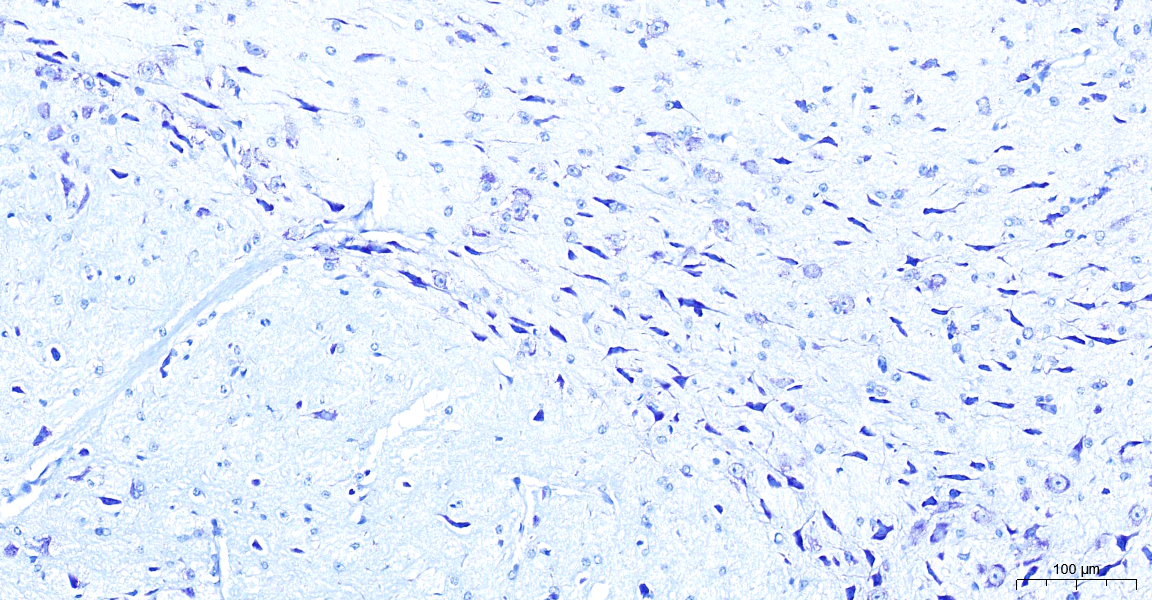

Supplement: Supplementary file 4 [file Data_Sheet_4.ZIP › Nissl's staining/65R║┌╓╩ ─ß╩╧_20.0x.tif]

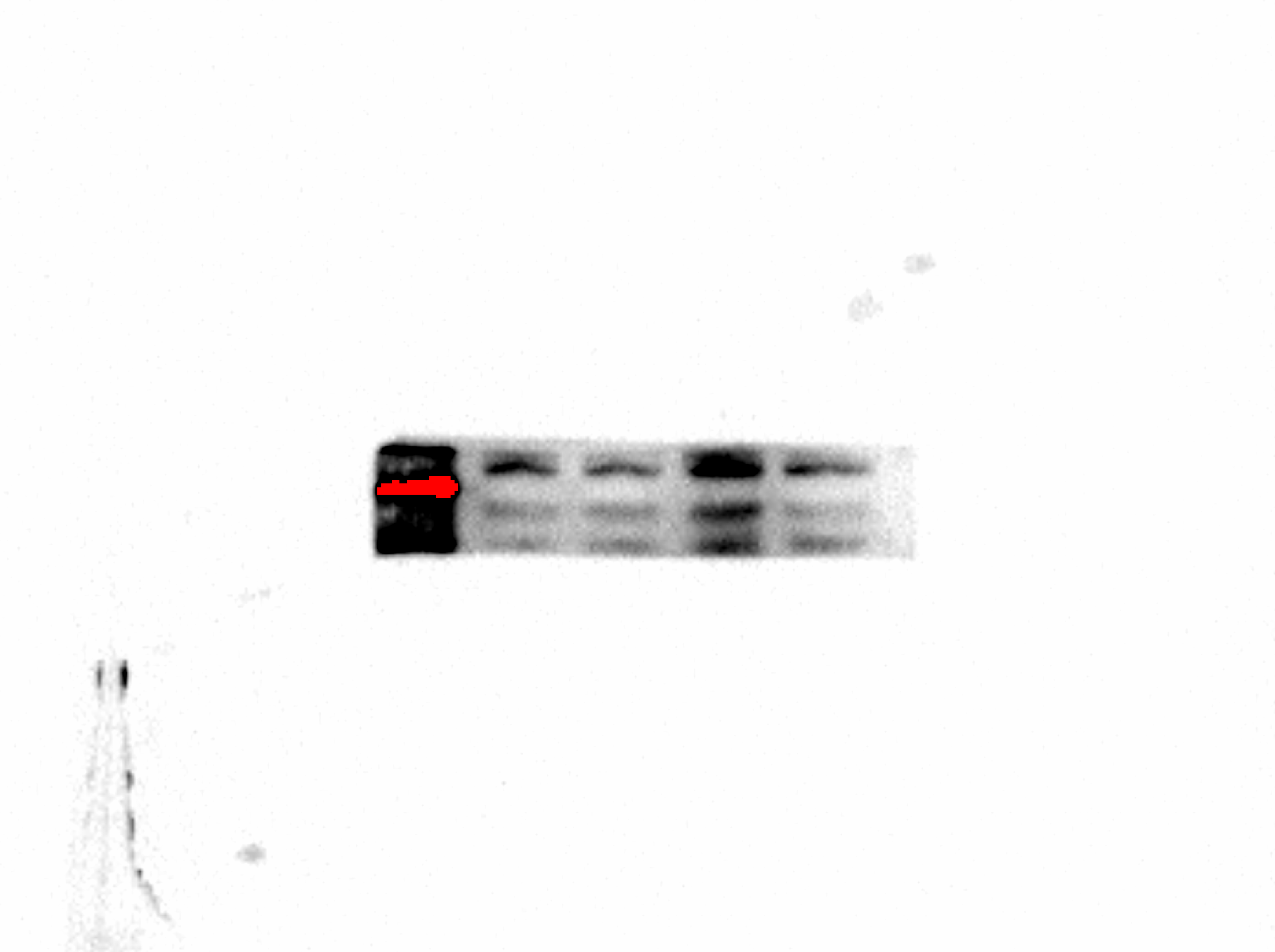

Supplement: Supplementary file 5 [file Data_Sheet_5.ZIP › Western blot/CD86/SN/╡╝│÷╖ó▓╝í╠╔╧═╝Administrator 2023-07-18_19h41m50s_Exposure_23.0sec - ╕▒▒╛.tif]

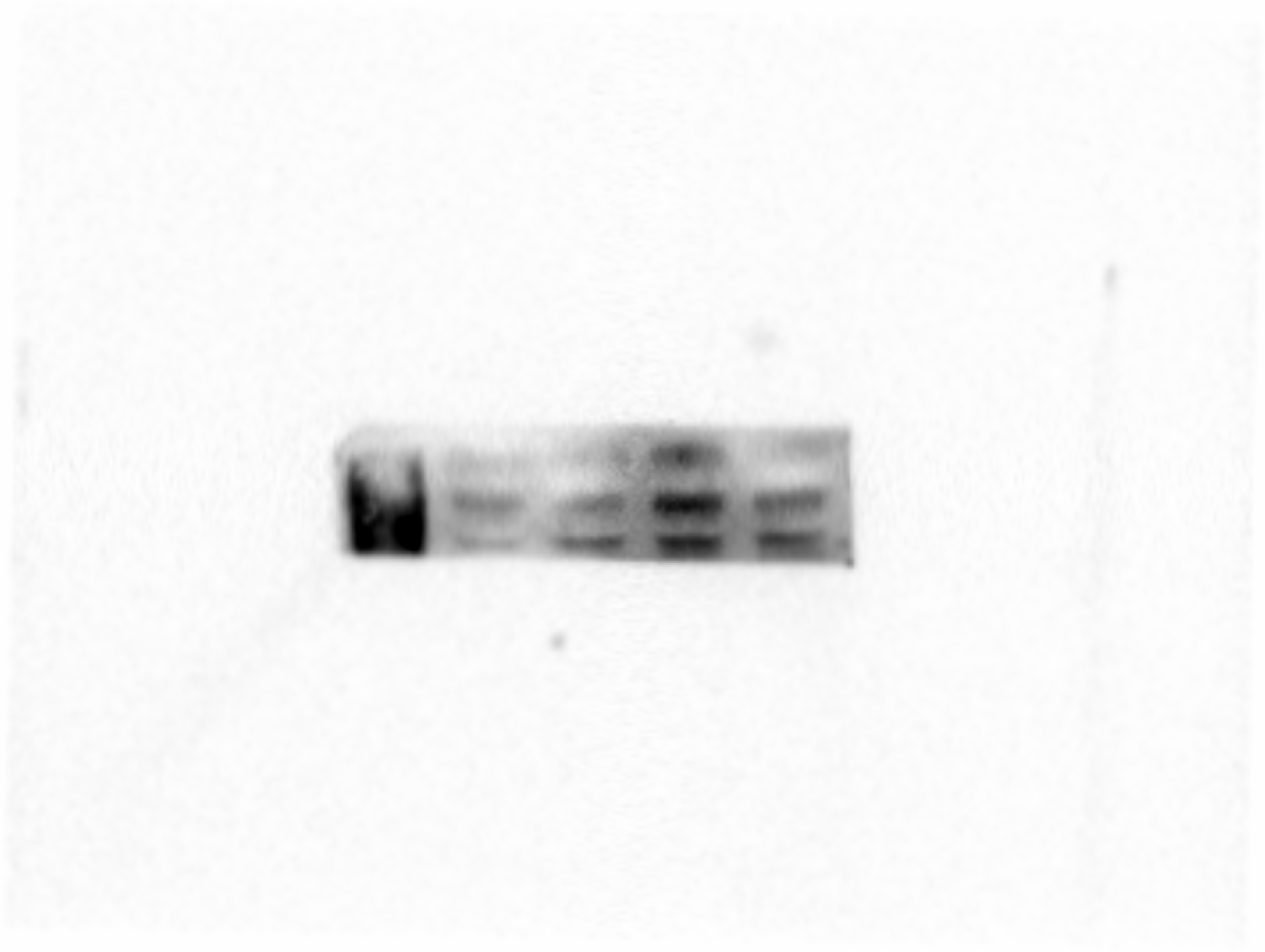

Supplement: Supplementary file 5 [file Data_Sheet_5.ZIP › Western blot/CD86/ST/Administrator 2023-07-13_23h42m31s_Exposure_29.0sec - ╕▒▒╛.tif]

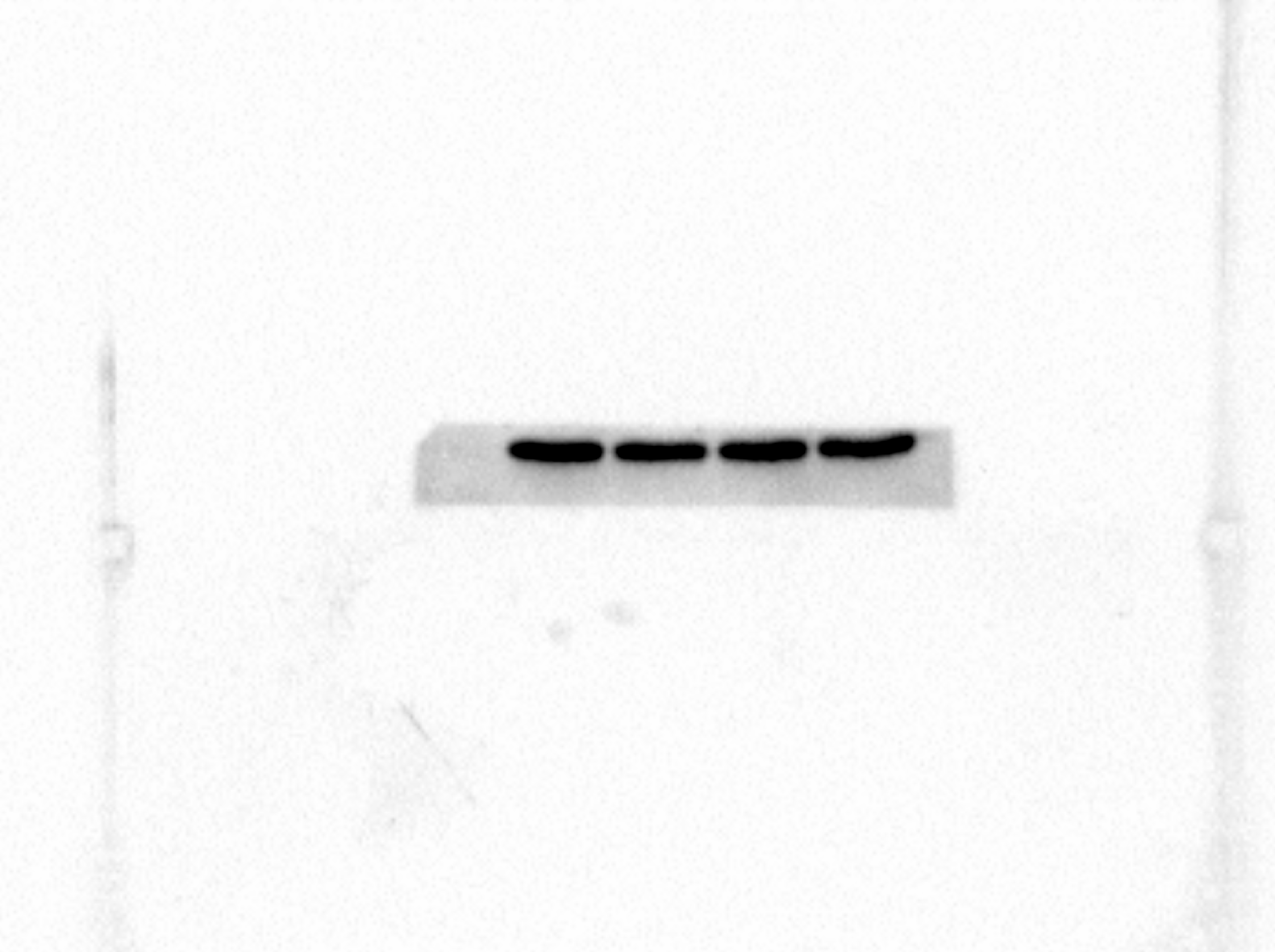

Supplement: Supplementary file 5 [file Data_Sheet_5.ZIP › Western blot/GAPDH/SN/╖╓╬÷í╠╔╧═╝Administrator 2023-07-11_20h41m26s_Exposure_5.0sec - ╕▒▒╛.tif]

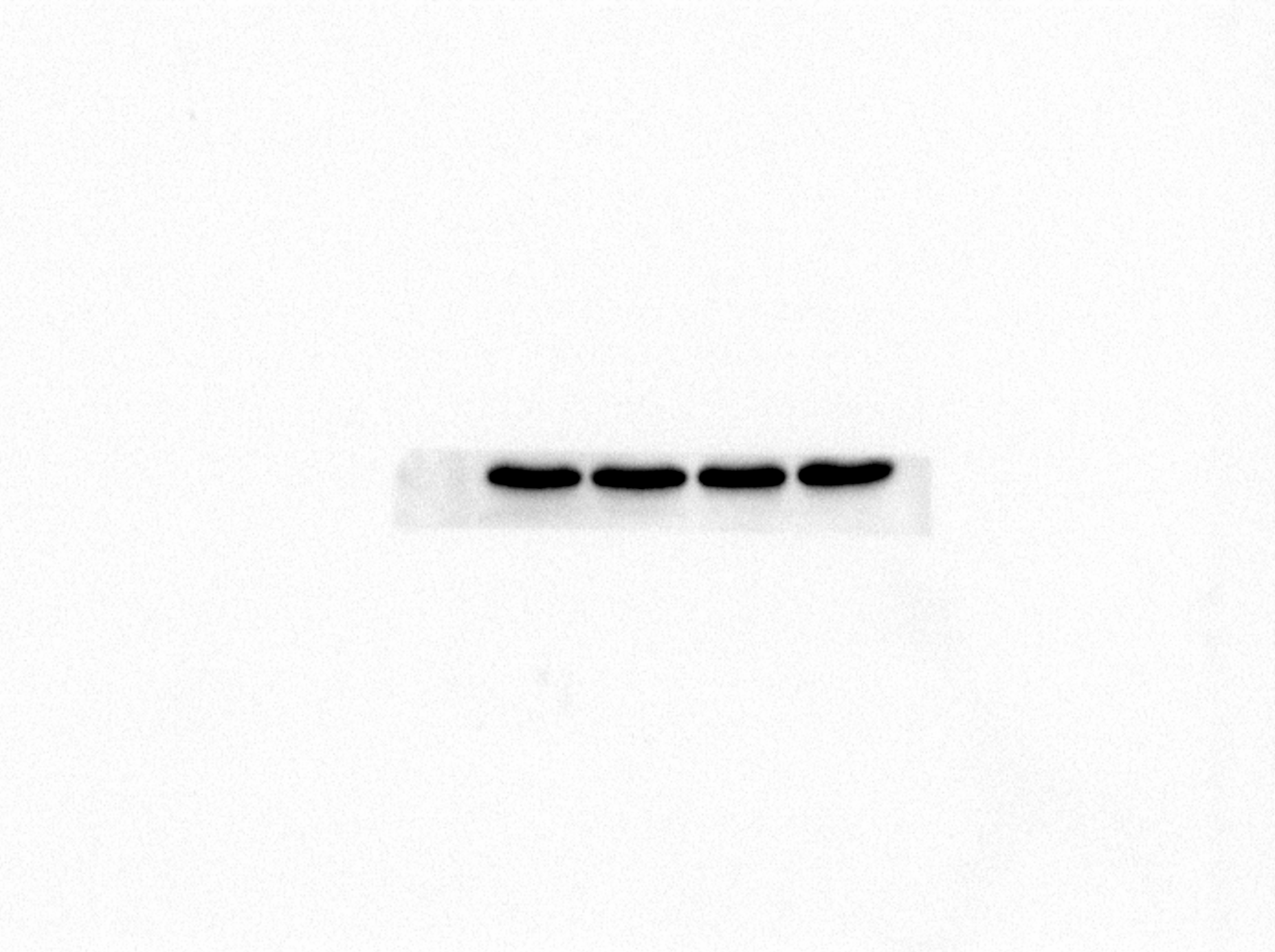

Supplement: Supplementary file 5 [file Data_Sheet_5.ZIP › Western blot/GAPDH/SN/╖╓╬÷í╠╔╧═╝Administrator 2023-07-13_22h47m57s_Exposure_8.0sec - ╕▒▒╛.tif]

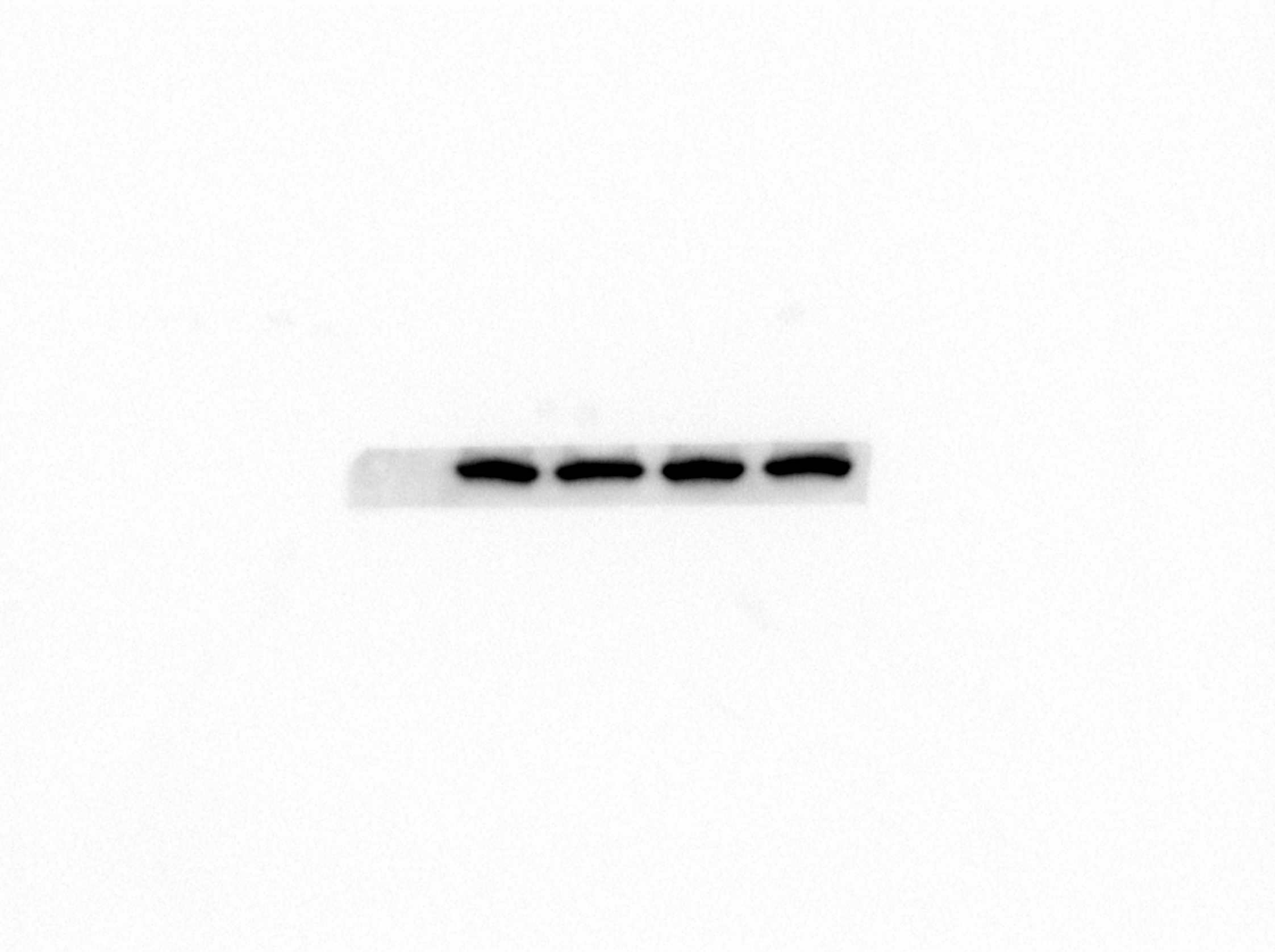

Supplement: Supplementary file 5 [file Data_Sheet_5.ZIP › Western blot/GAPDH/ST/í╠╔╧═╝Administrator 2023-07-13_23h01m57s_Exposure_7.0sec.tif]

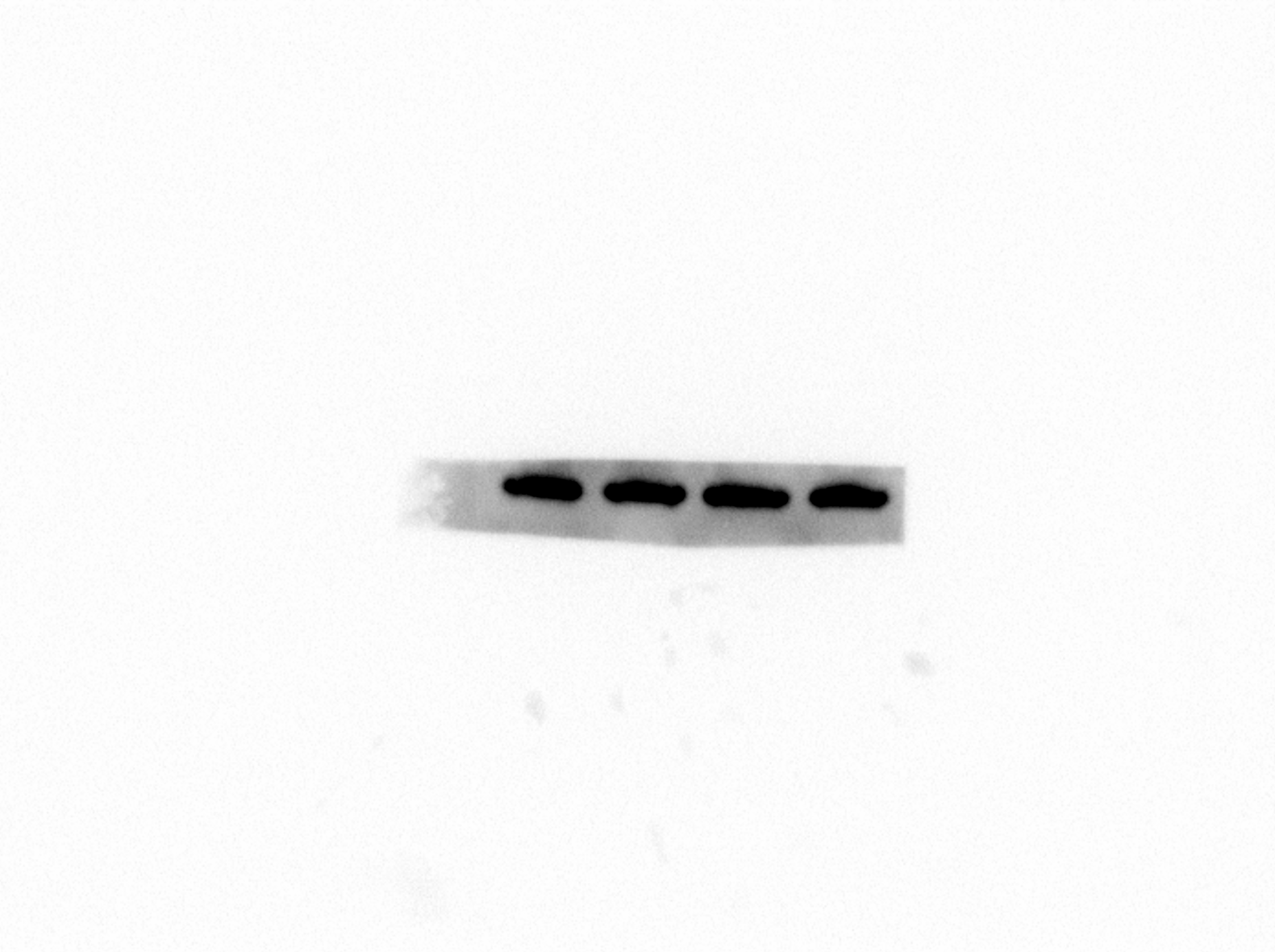

Supplement: Supplementary file 5 [file Data_Sheet_5.ZIP › Western blot/GAPDH/ST/í╠╔╧═╝Administrator 2023-07-14_20h44m50s_Exposure_3.0sec.tif]

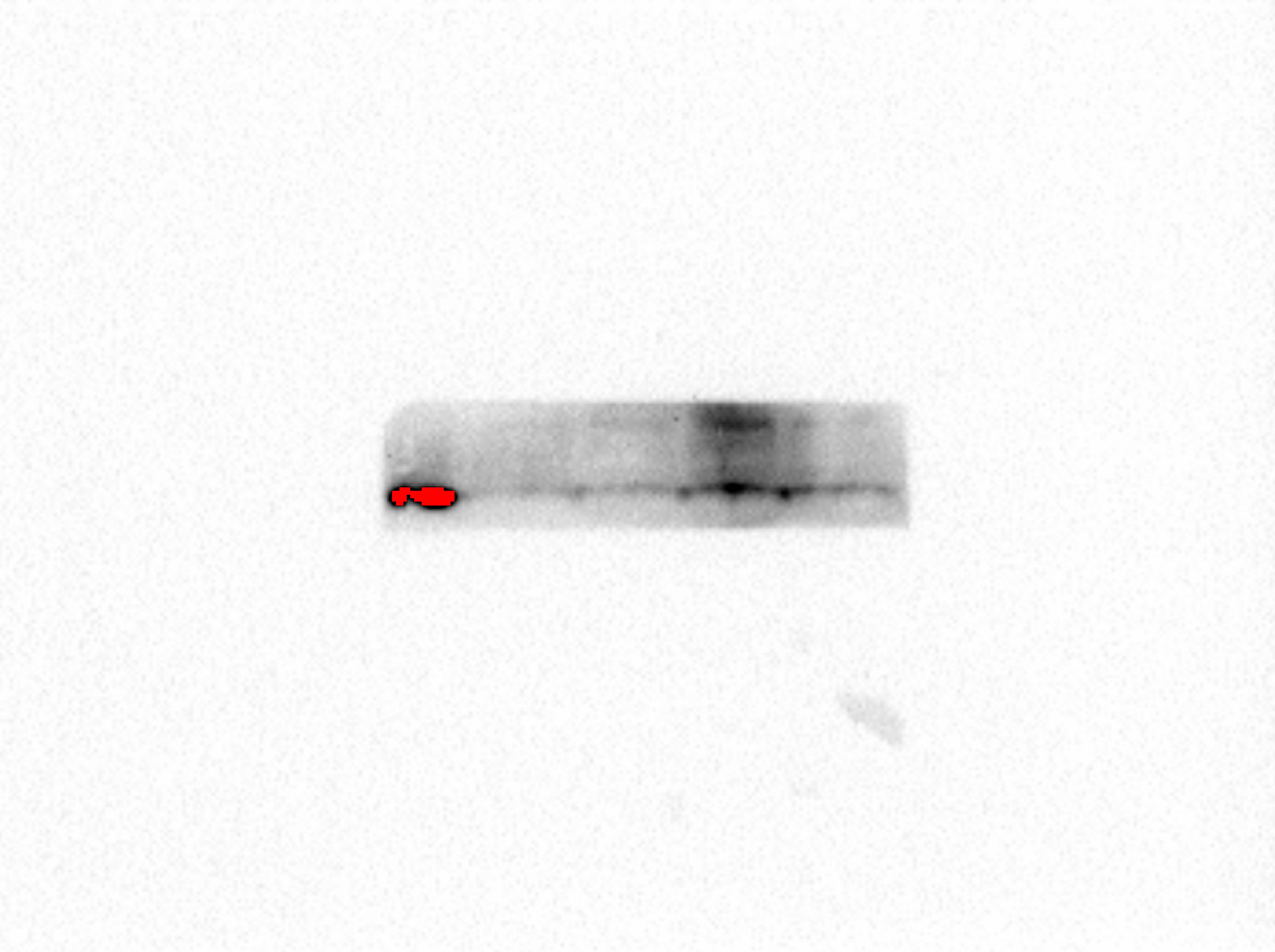

Supplement: Supplementary file 5 [file Data_Sheet_5.ZIP › Western blot/Iba1/SN/í╠╔╧═╝Administrator 2023-07-14_23h52m14s_Exposure_30.0sec.tif]

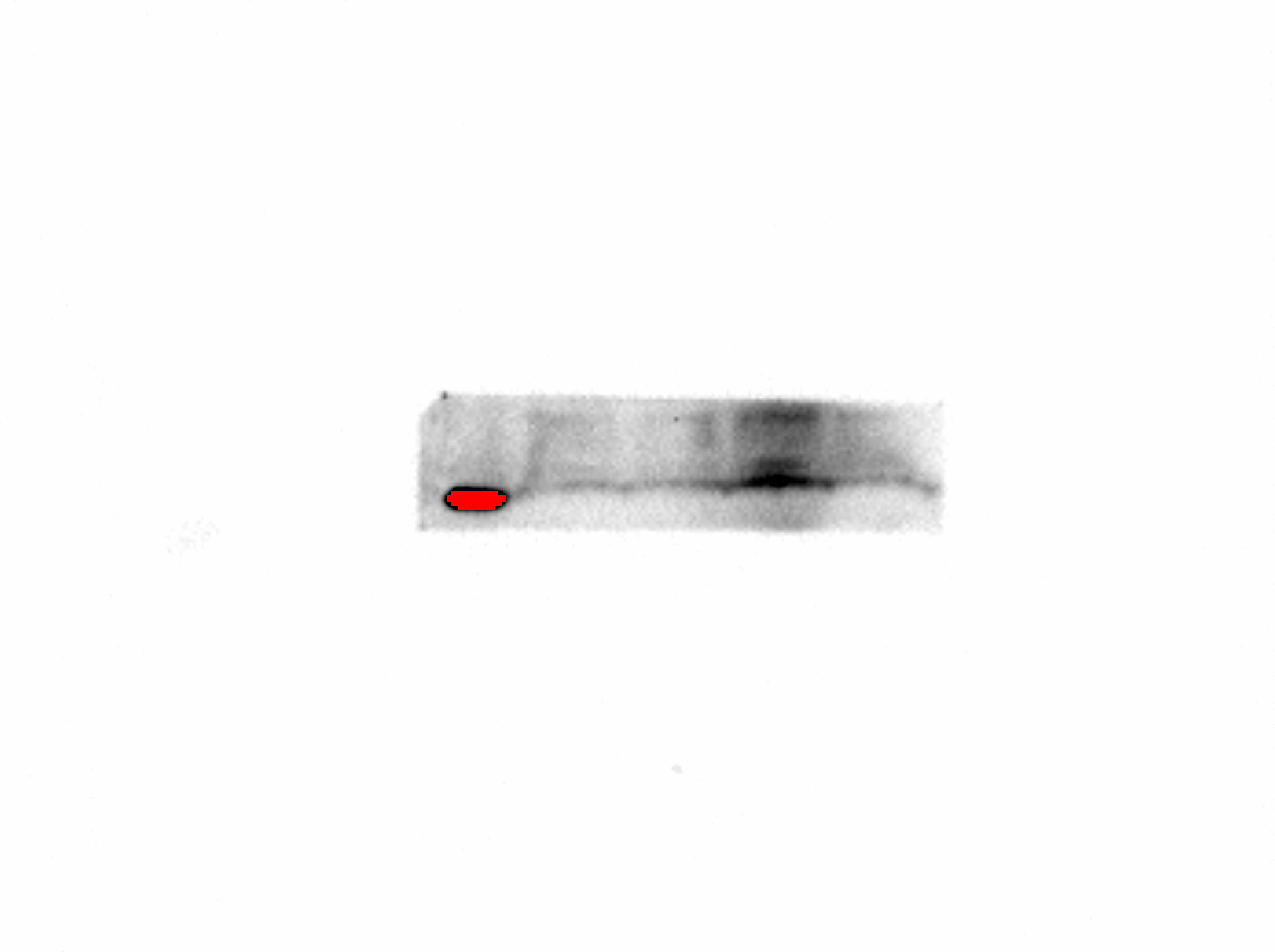

Supplement: Supplementary file 5 [file Data_Sheet_5.ZIP › Western blot/Iba1/ST/í╠╔╧═╝Administrator 2023-07-15_00h04m27s_Exposure_29.0sec.tif]

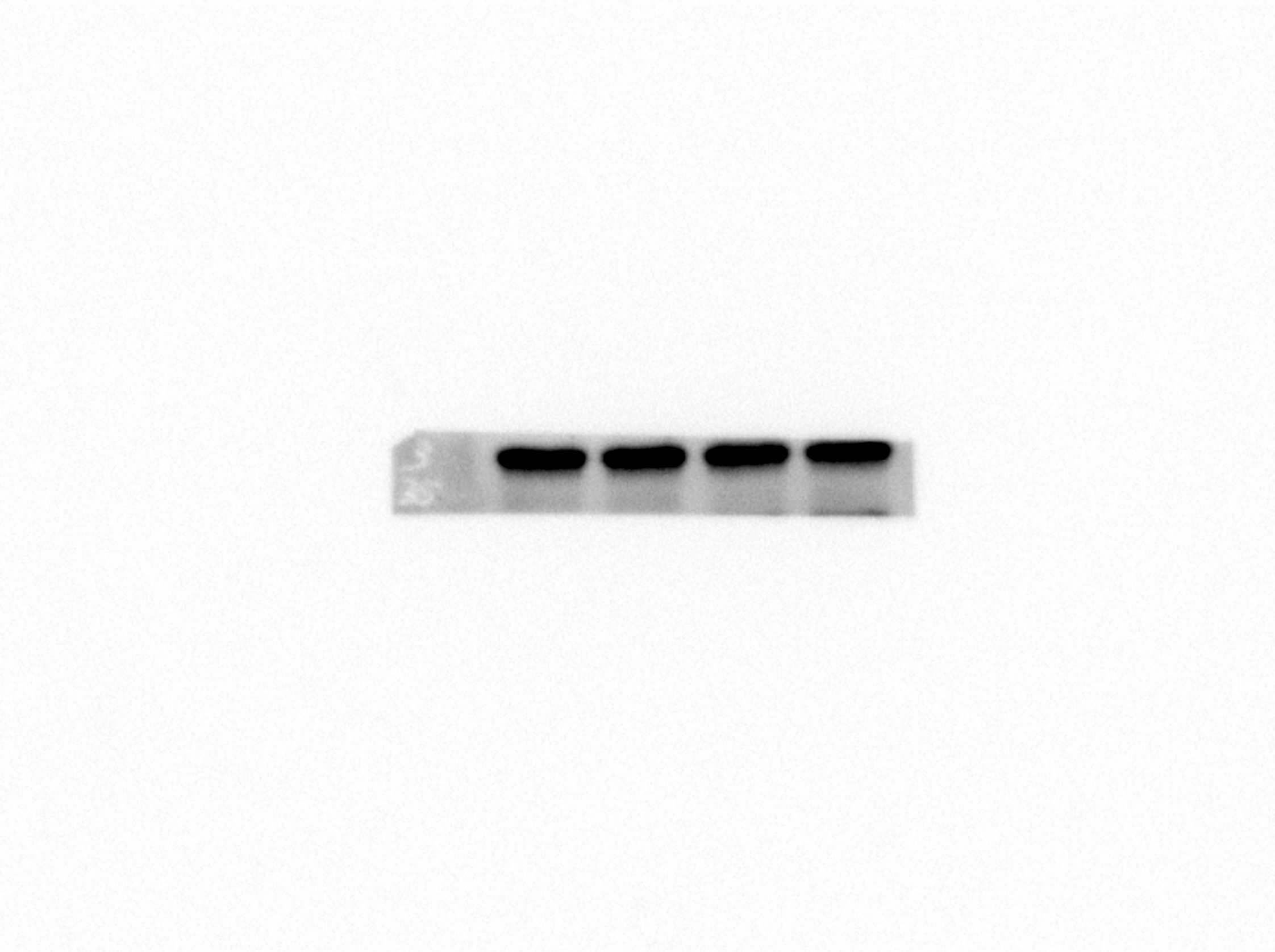

Supplement: Supplementary file 5 [file Data_Sheet_5.ZIP › Western blot/IKB/SN/í╠╔╧═╝Administrator 2023-07-14_21h16m47s_Exposure_3.0sec.tif]

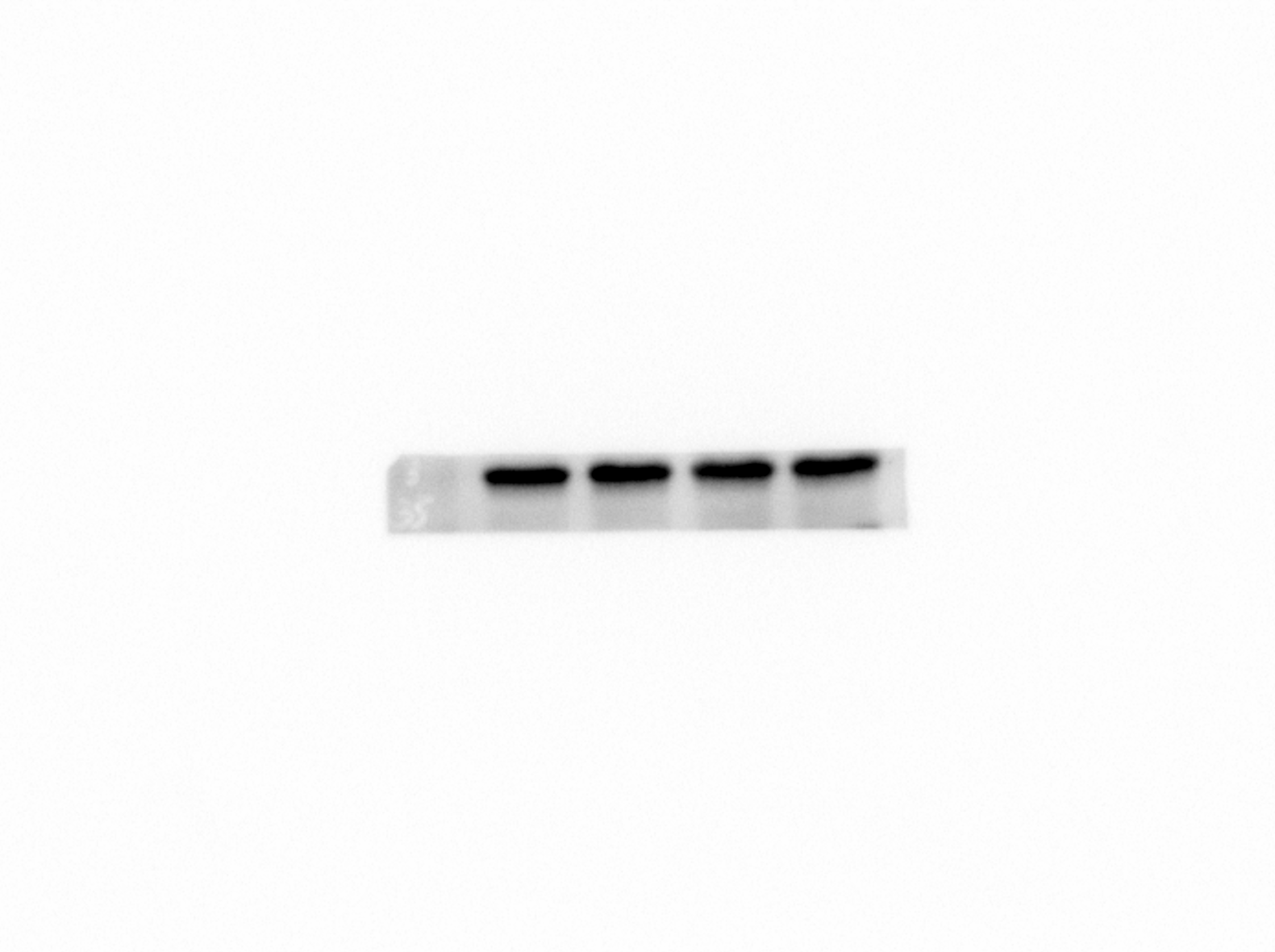

Supplement: Supplementary file 5 [file Data_Sheet_5.ZIP › Western blot/IKB/ST/í╠╔╧═╝Administrator 2023-07-14_21h37m55s_Exposure_2.0sec.tif]

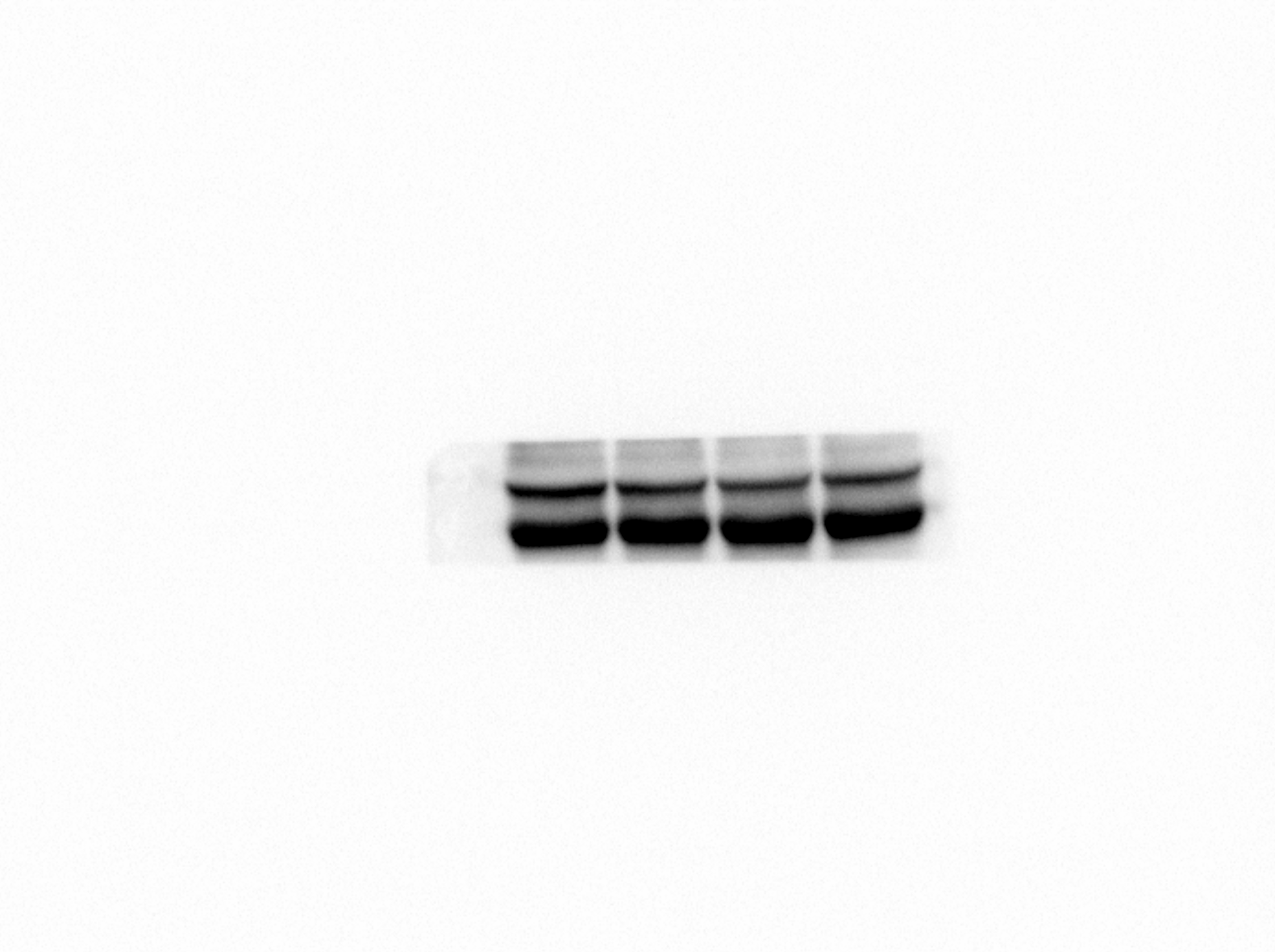

Supplement: Supplementary file 5 [file Data_Sheet_5.ZIP › Western blot/JNK/SN/í╠╔╧═╝Administrator 2023-07-14_22h45m12s_Exposure_4.0sec.tif]

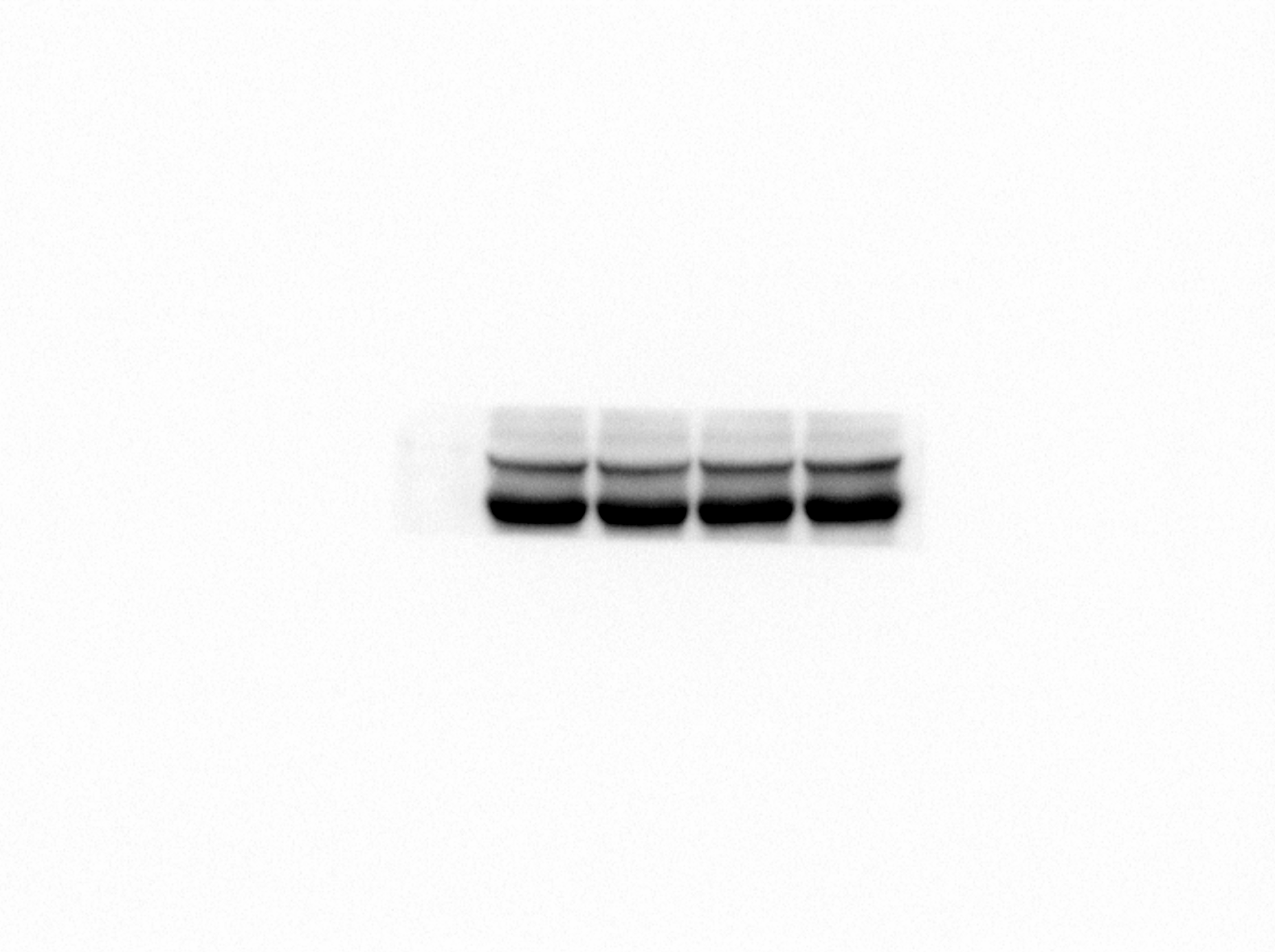

Supplement: Supplementary file 5 [file Data_Sheet_5.ZIP › Western blot/JNK/ST/í╠╔╧═╝Administrator 2023-07-14_23h08m20s_Exposure_5.0sec.tif]

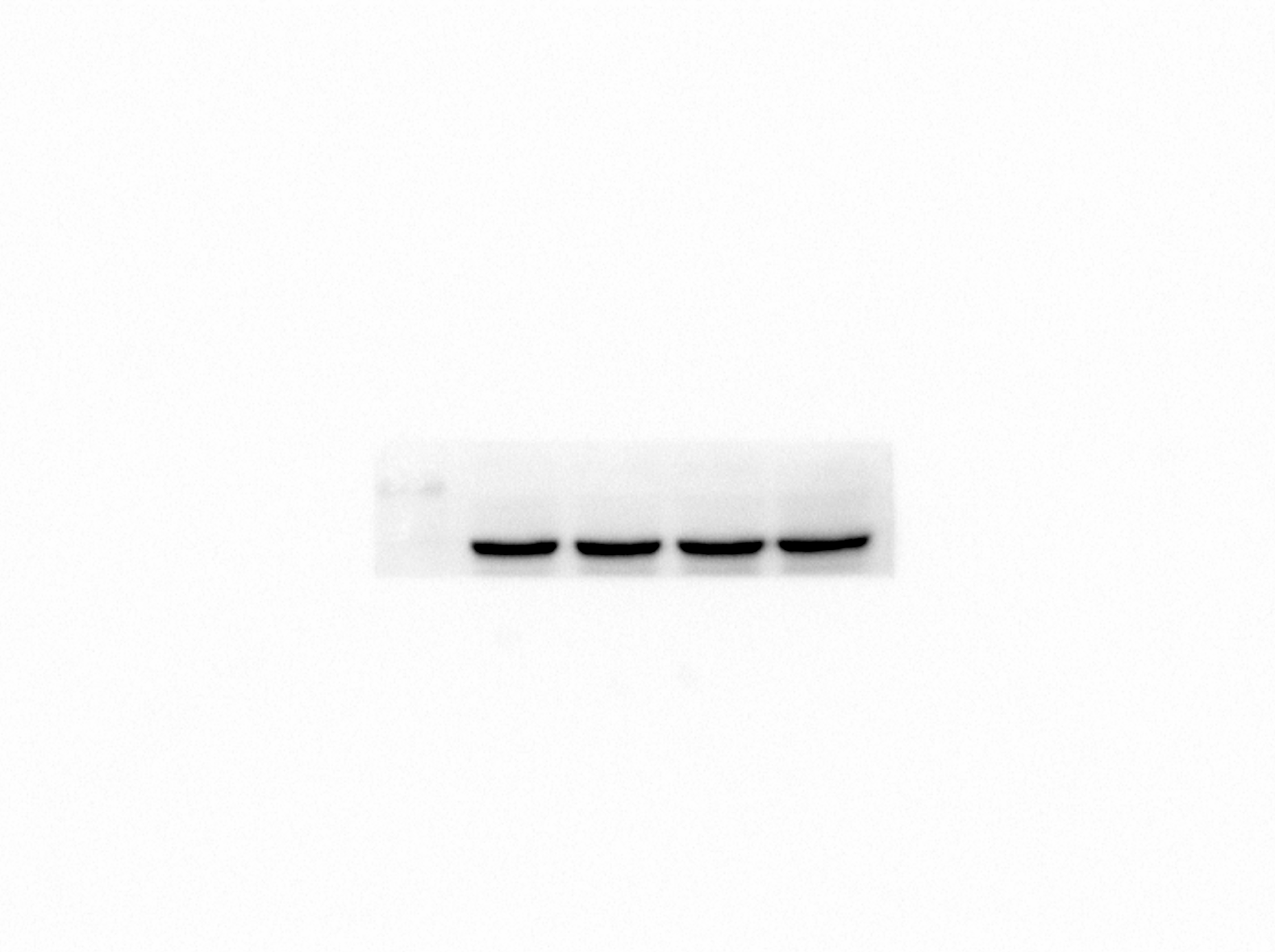

Supplement: Supplementary file 5 [file Data_Sheet_5.ZIP › Western blot/P38/SN/í╠╔╧═╝Administrator 2023-07-14_22h10m51s_Exposure_3.0sec.tif]

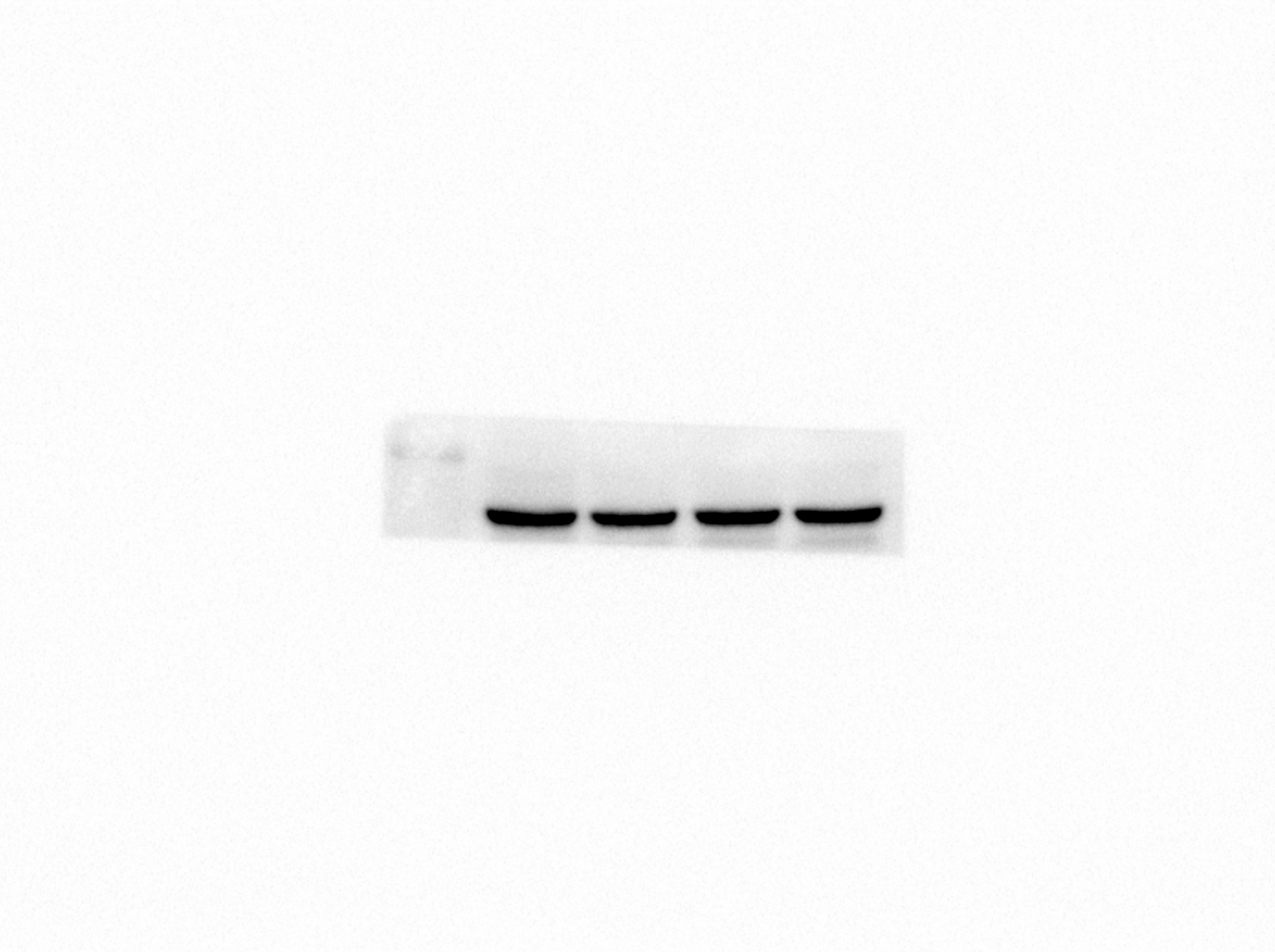

Supplement: Supplementary file 5 [file Data_Sheet_5.ZIP › Western blot/P38/ST/í╠╔╧═╝Administrator 2023-07-14_22h54m55s_Exposure_4.0sec.tif]

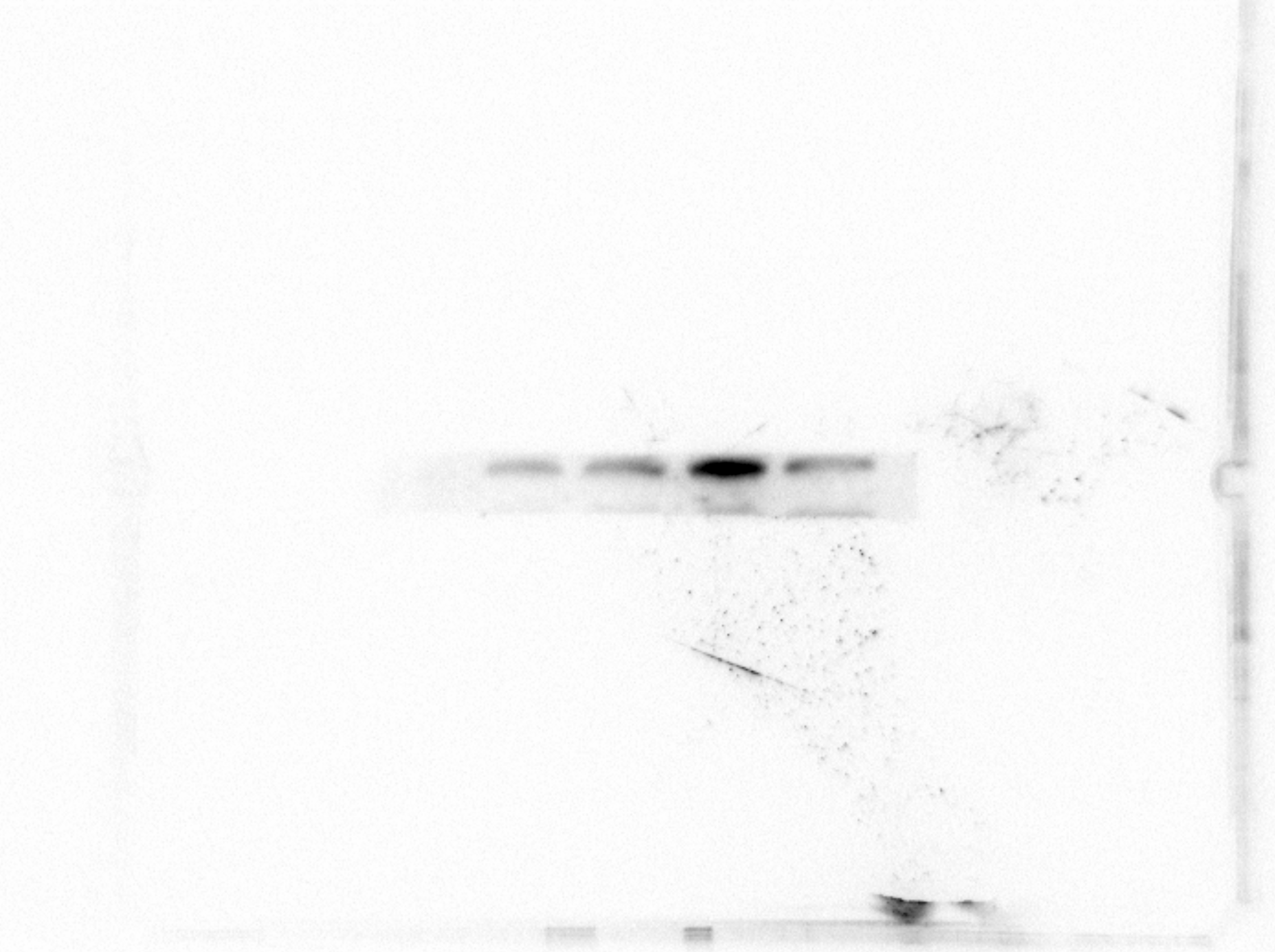

Supplement: Supplementary file 5 [file Data_Sheet_5.ZIP › Western blot/pIKB/SN/í╠╔╧═╝Administrator 2023-07-11_21h15m10s_Exposure_10.0sec.tif]

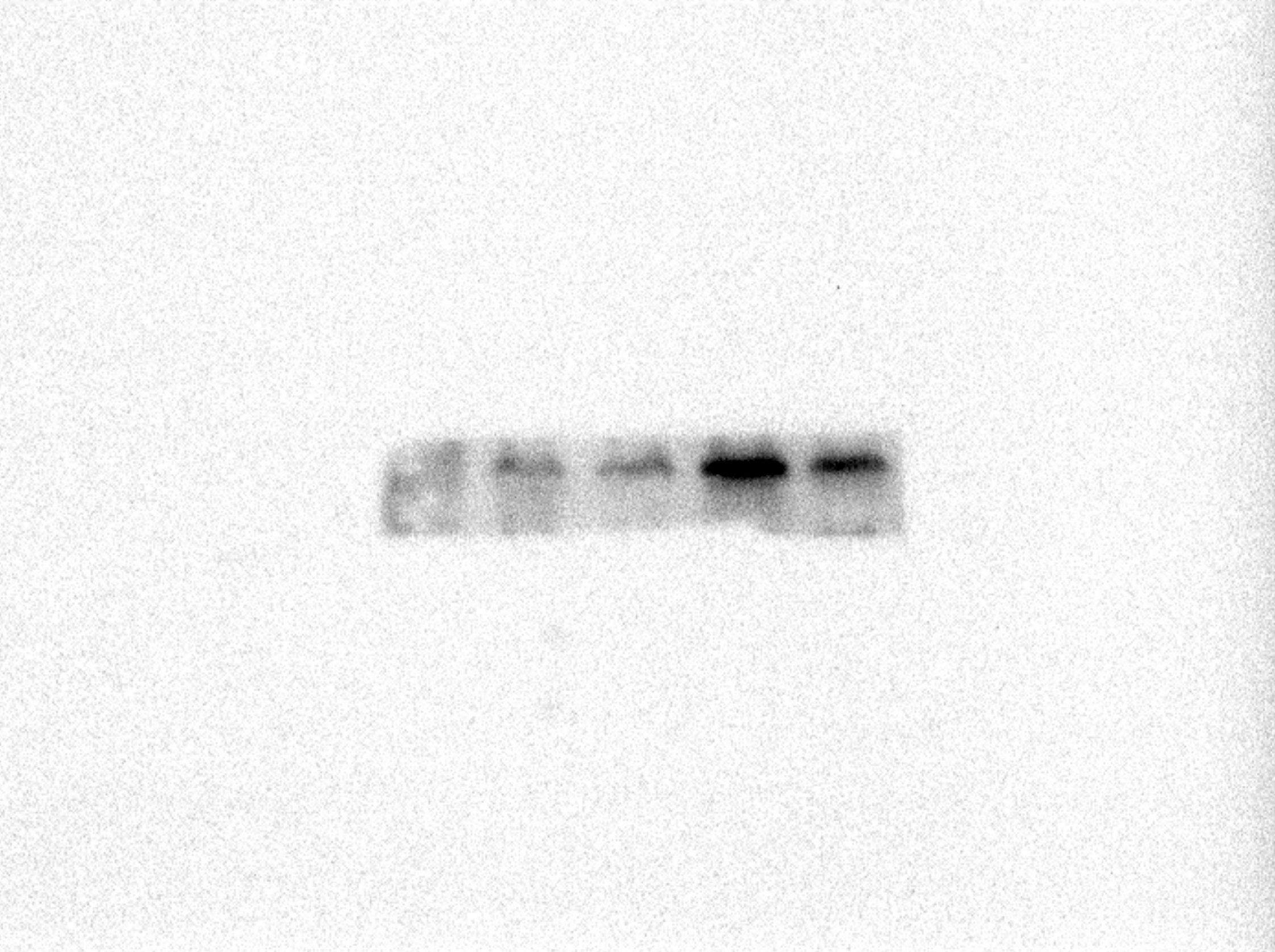

Supplement: Supplementary file 5 [file Data_Sheet_5.ZIP › Western blot/pIKB/ST/í╠╔╧═╝dell 2023-07-18 23 ╩▒ 13 ╖╓_Exposure_70.0sec.tif]

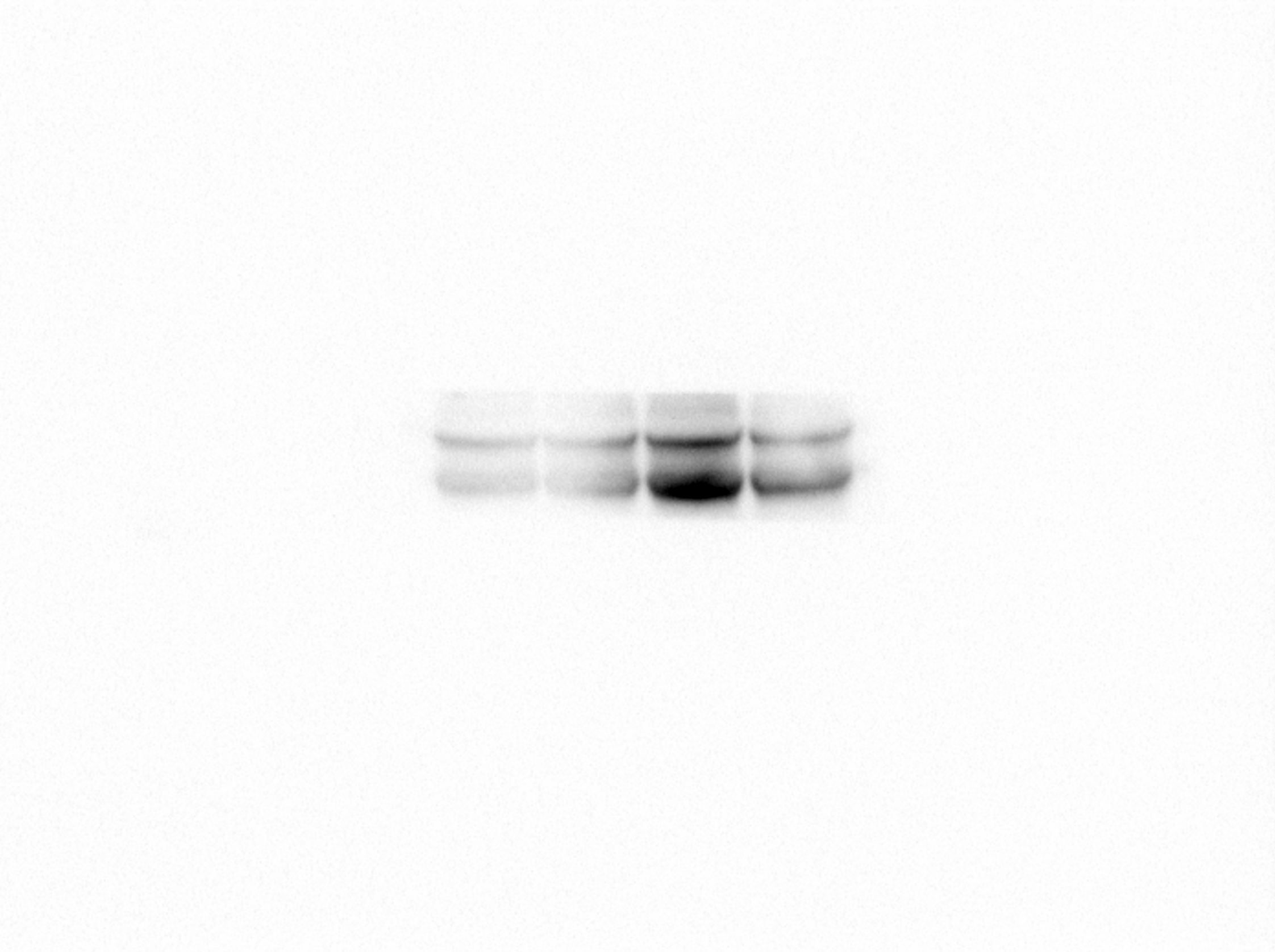

Supplement: Supplementary file 5 [file Data_Sheet_5.ZIP › Western blot/pJNK/SN/í╠╔╧═╝Administrator 2023-07-14_22h35m11s_Exposure_12.0sec.tif]

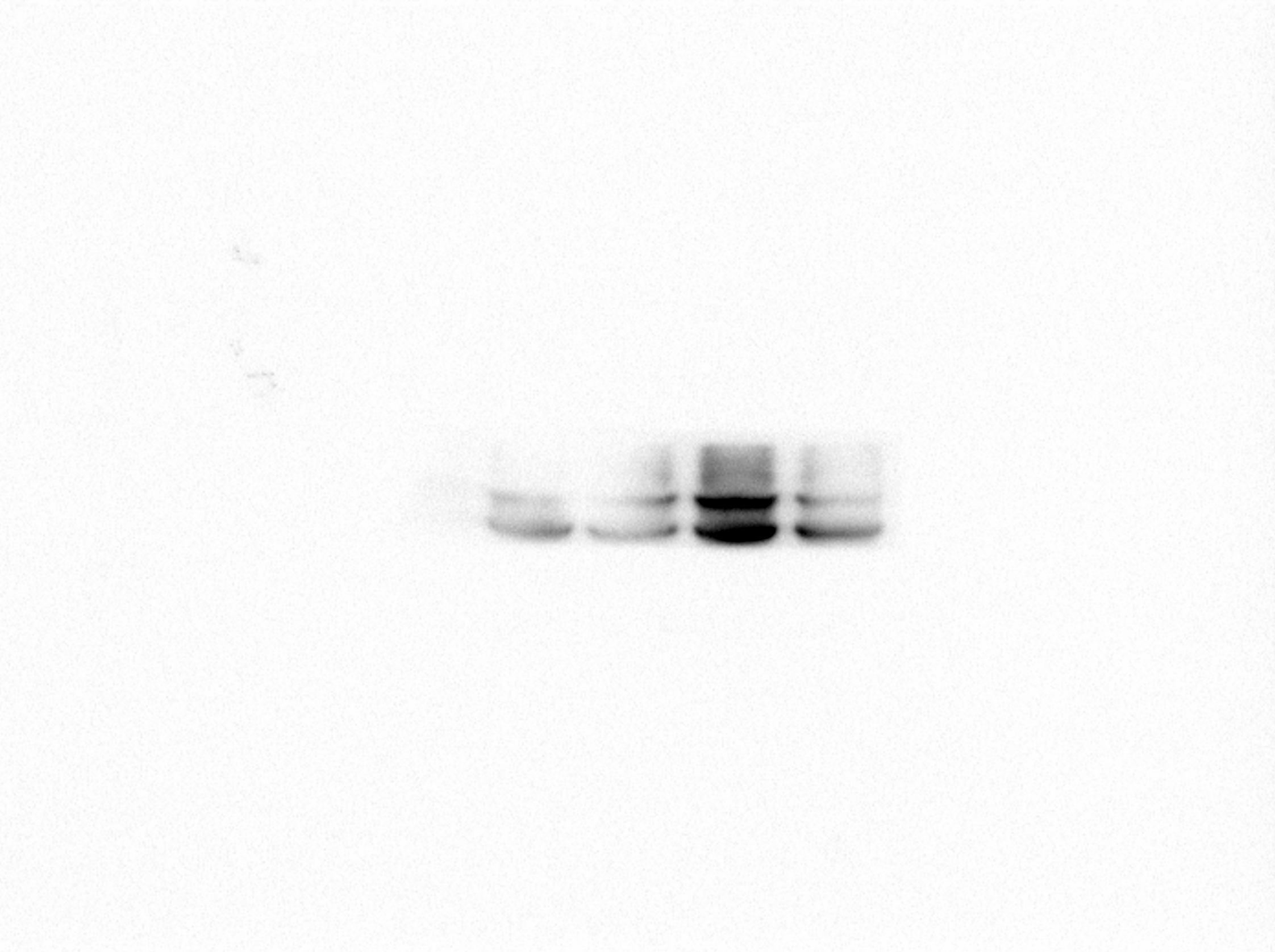

Supplement: Supplementary file 5 [file Data_Sheet_5.ZIP › Western blot/pJNK/ST/í╠╔╧═╝Administrator 2023-07-11_23h06m47s_Exposure_17.0sec.tif]

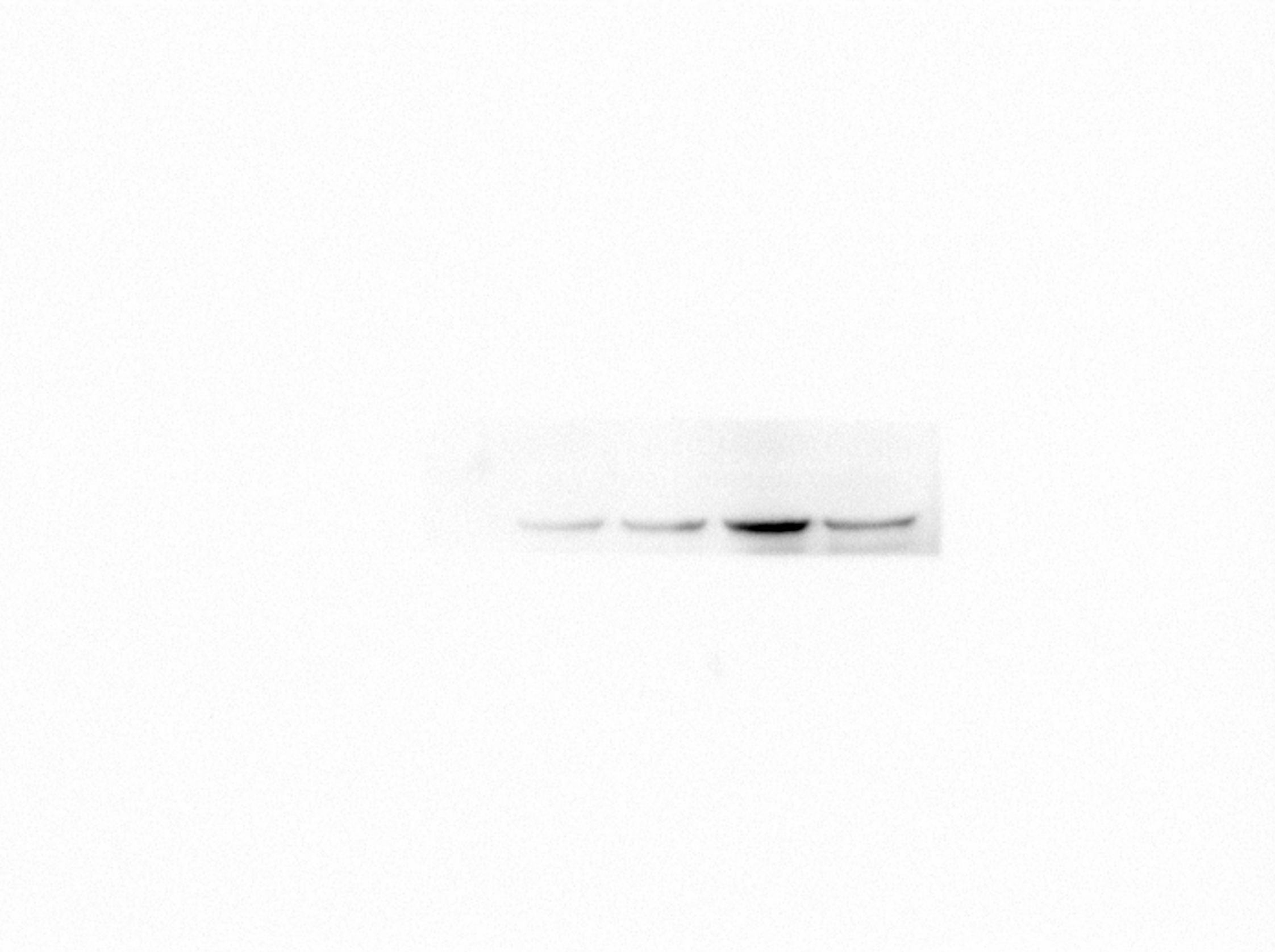

Supplement: Supplementary file 5 [file Data_Sheet_5.ZIP › Western blot/pP38/SN/í╠╔╧═╝Administrator 2023-07-14_22h17m25s_Exposure_10.0sec.tif]

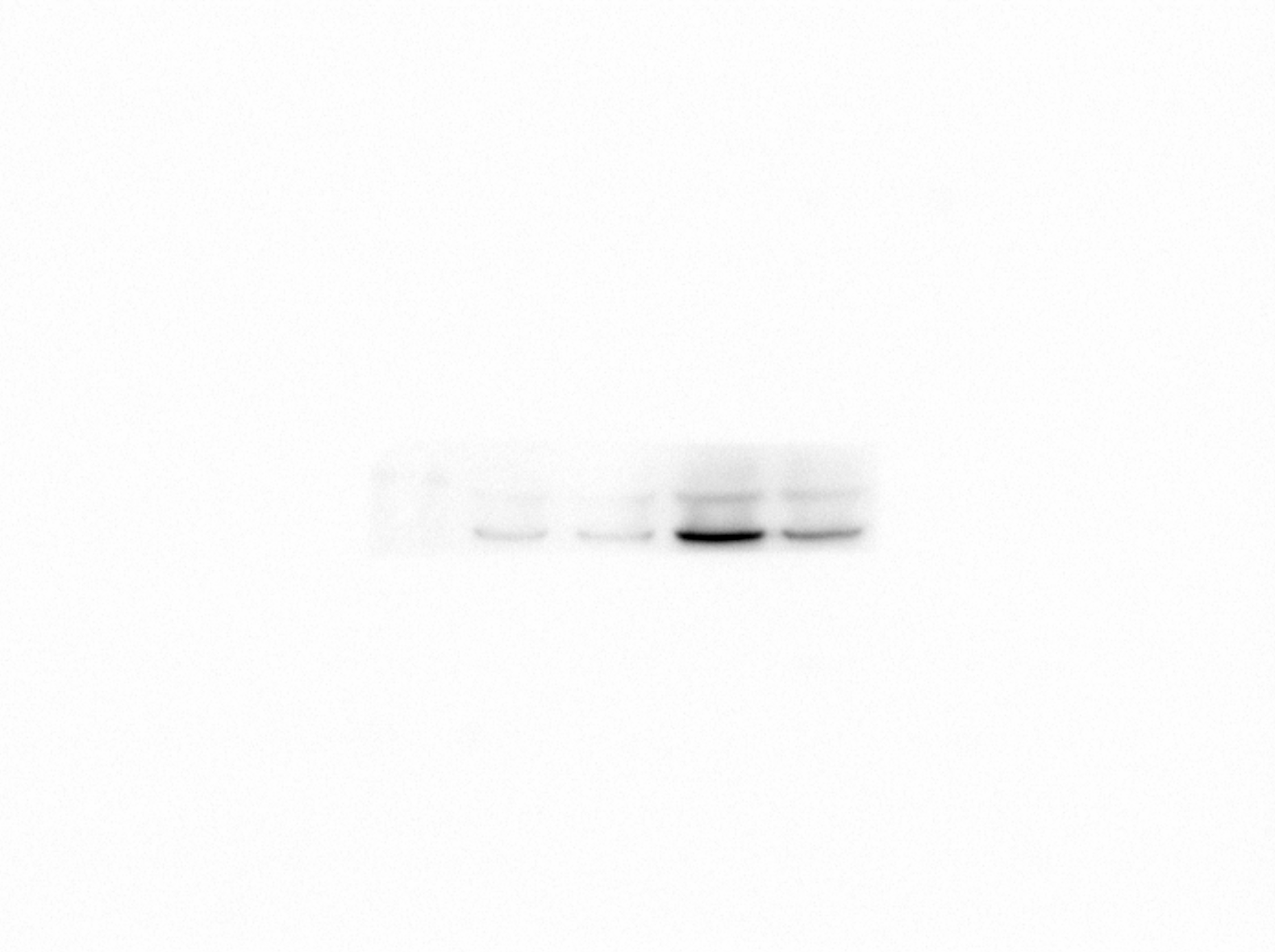

Supplement: Supplementary file 5 [file Data_Sheet_5.ZIP › Western blot/pP38/ST/í╠╔╧═╝Administrator 2023-07-17_23h33m01s_Exposure_7.0sec.tif]

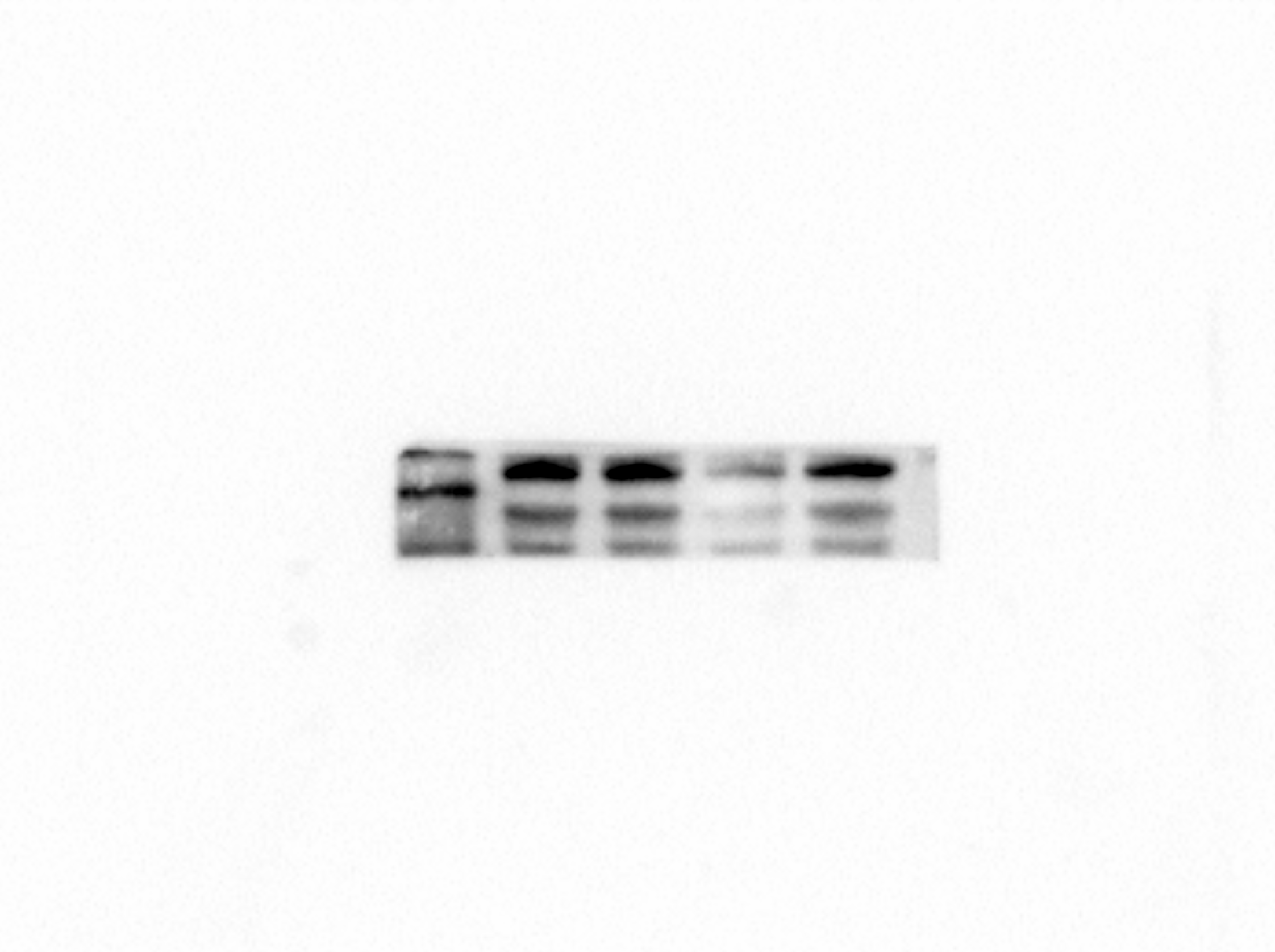

Supplement: Supplementary file 5 [file Data_Sheet_5.ZIP › Western blot/TH/SN/í╠╔╧═╝Administrator 2023-07-13_21h50m08s_Exposure_3.0sec.tif]

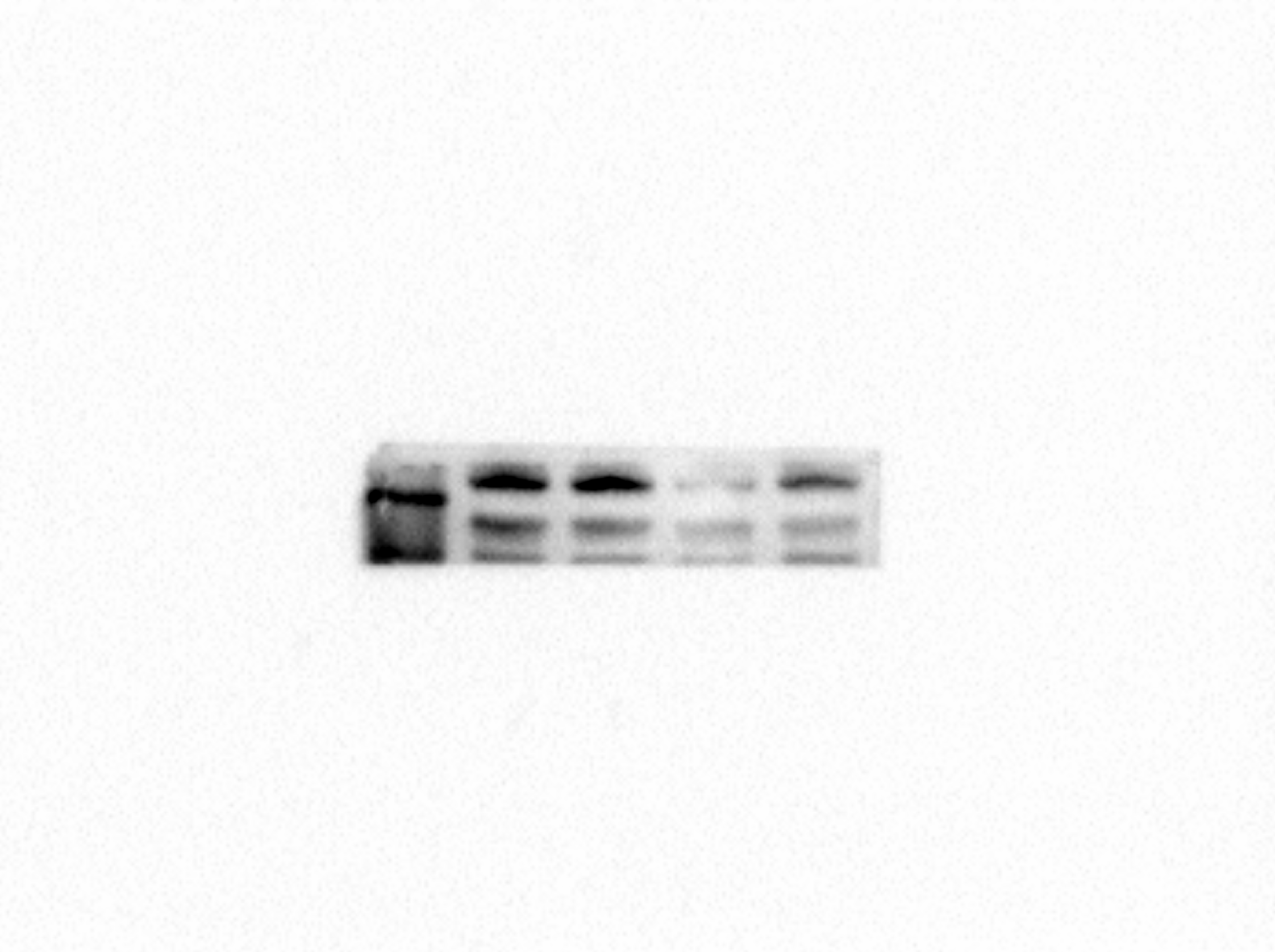

Supplement: Supplementary file 5 [file Data_Sheet_5.ZIP › Western blot/TH/ST/í╠╔╧═╝Administrator 2023-07-13_22h15m32s_Exposure_10.0sec.tif]

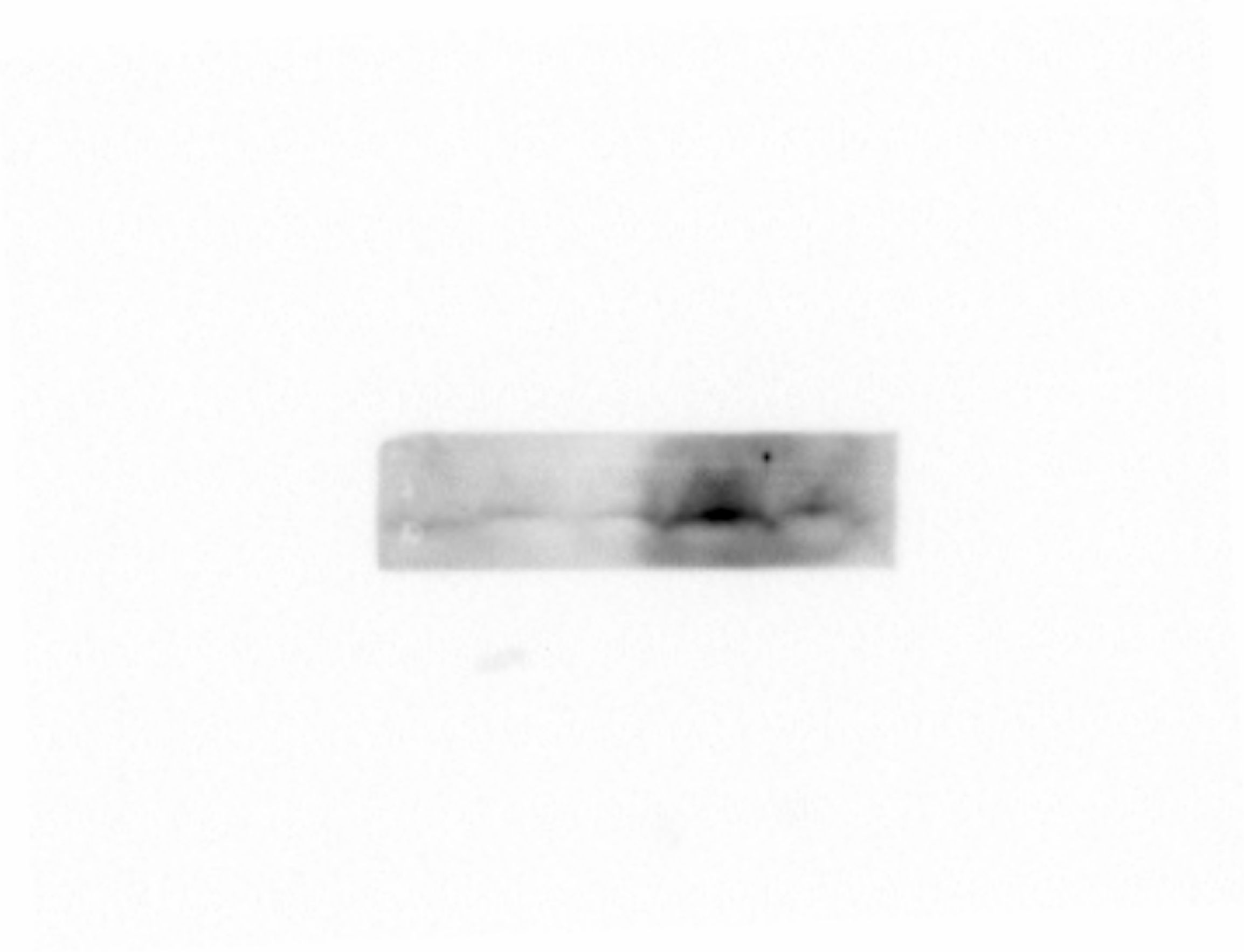

Supplement: Supplementary file 5 [file Data_Sheet_5.ZIP › Western blot/a┴-Synuclein/SN/í╠╔╧═╝Administrator 2023-07-14_23h41m24s_Exposure_4.0sec.tif]

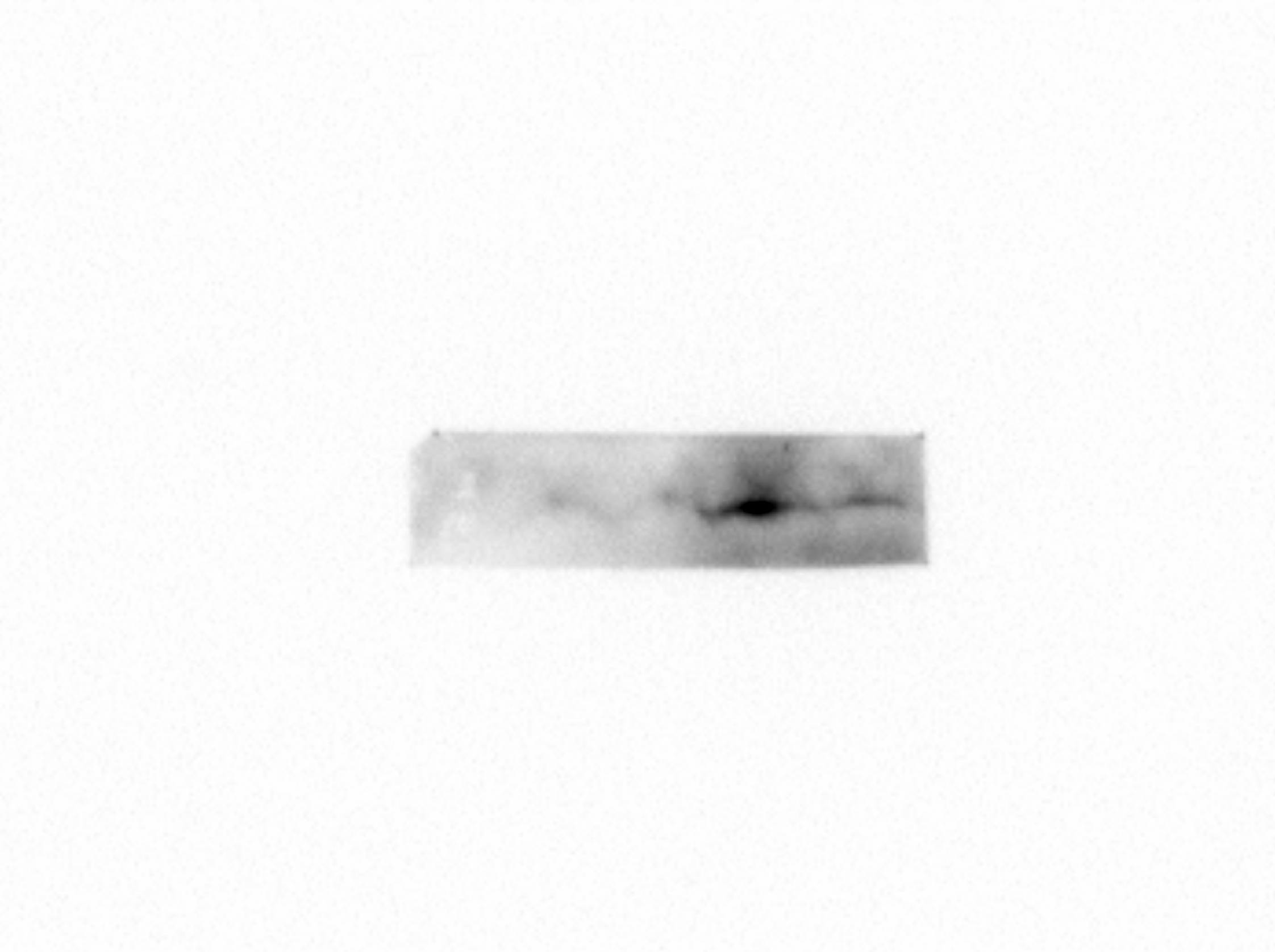

Supplement: Supplementary file 5 [file Data_Sheet_5.ZIP › Western blot/a┴-Synuclein/ST/í╠╔╧═╝Administrator 2023-07-15_00h14m22s_Exposure_6.0sec.tif]

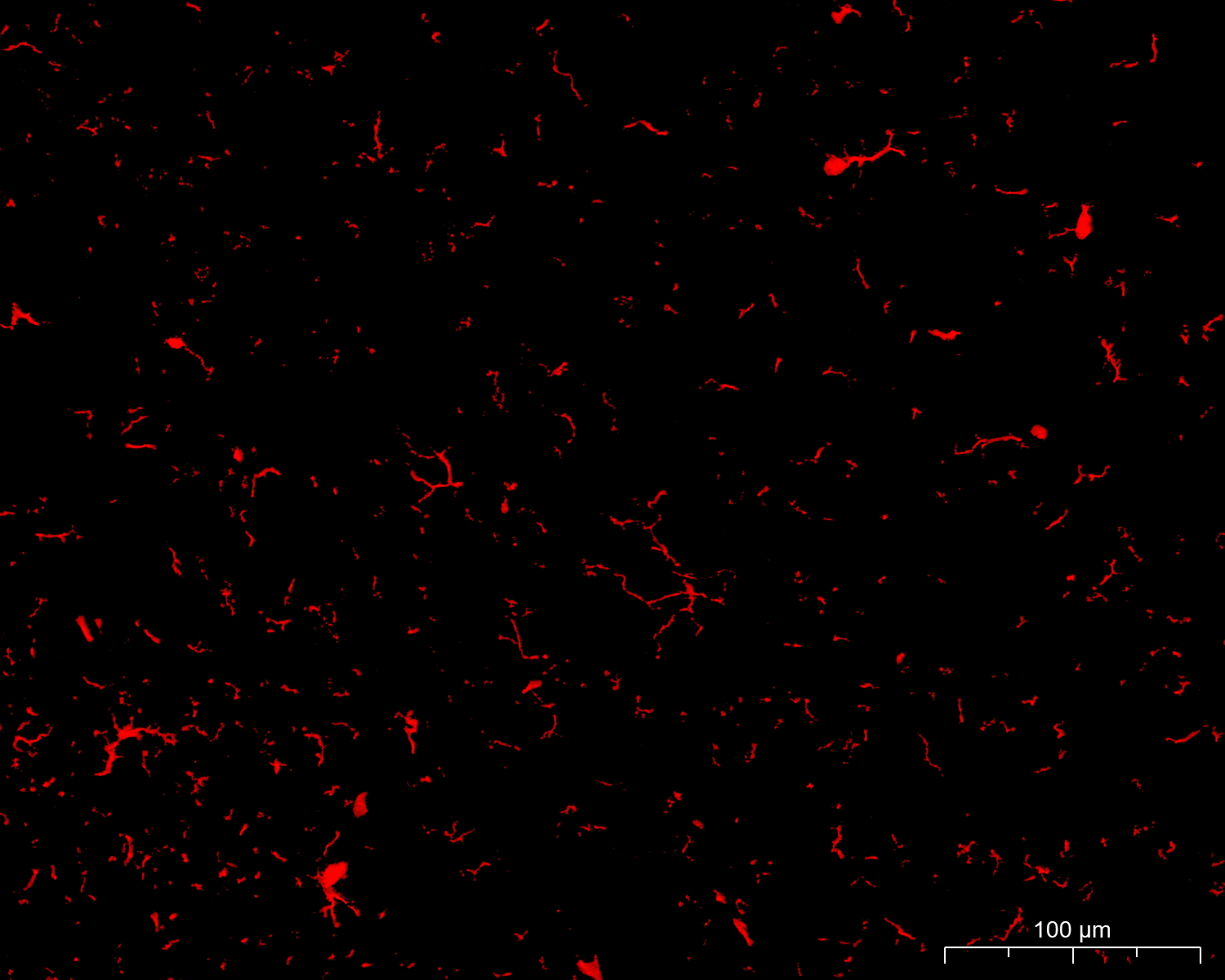

Supplement: Supplementary file 12 [file Data_Sheet_12.ZIP › SN/C/1.jpg]

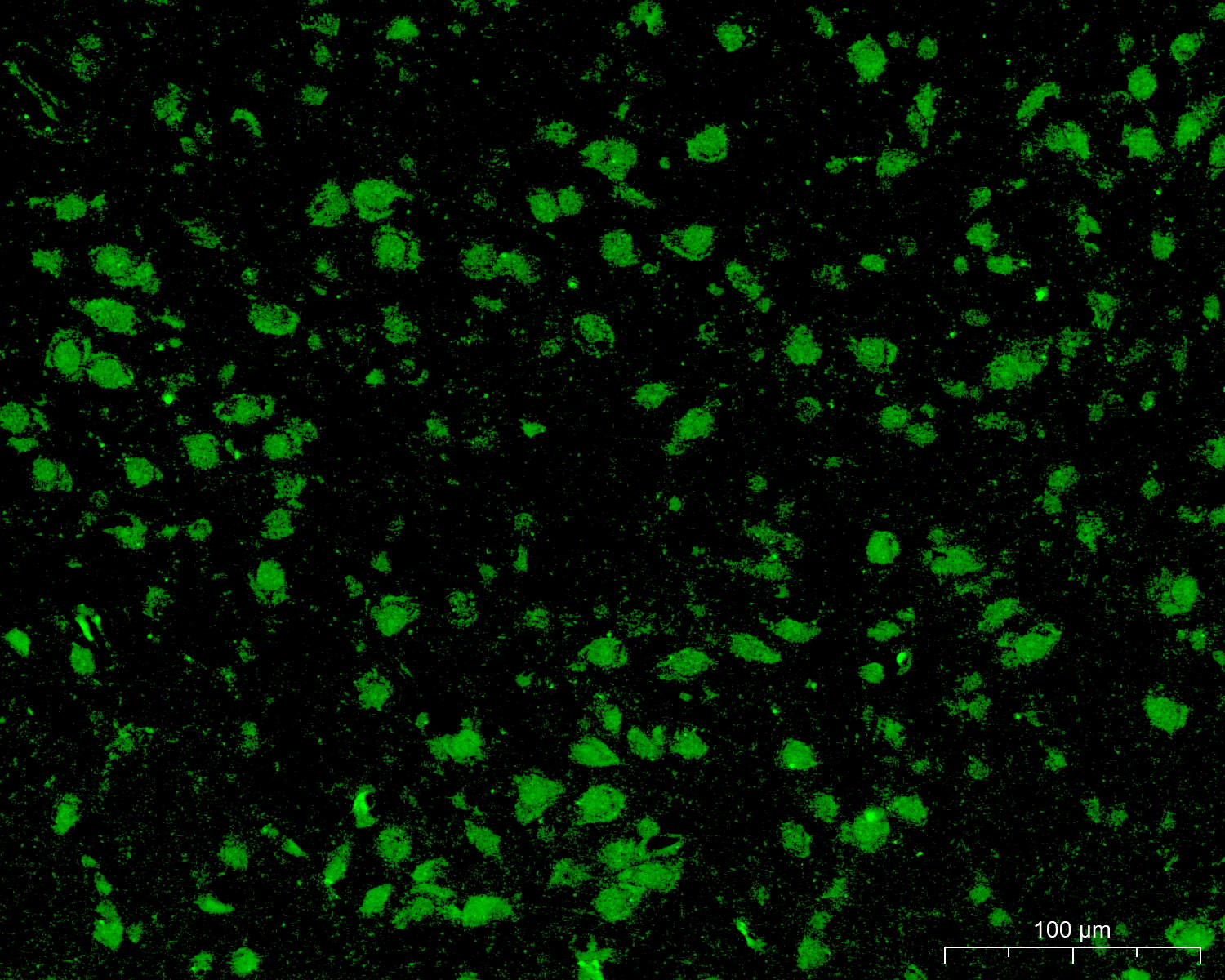

Supplement: Supplementary file 12 [file Data_Sheet_12.ZIP › SN/C/2.jpg]

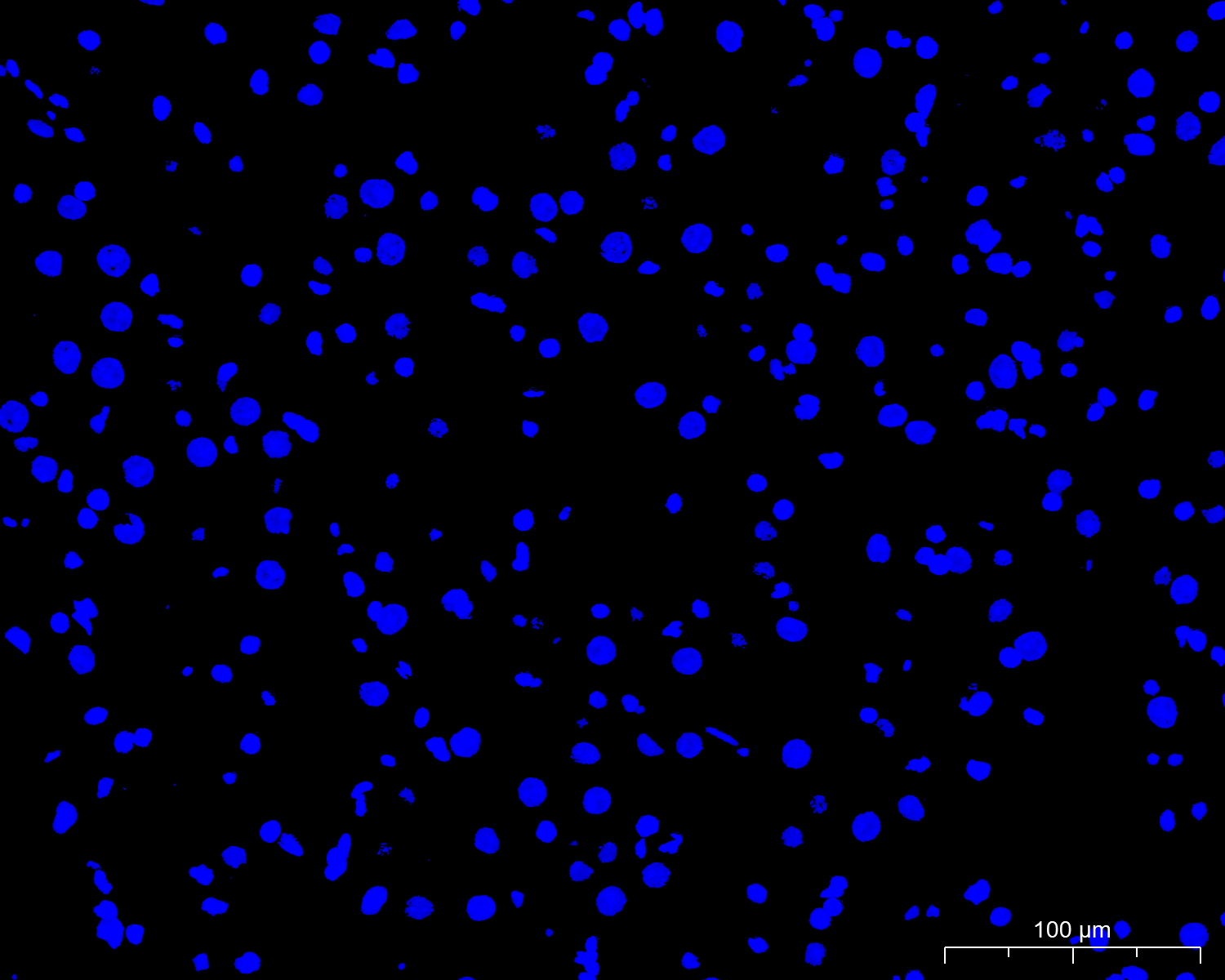

Supplement: Supplementary file 12 [file Data_Sheet_12.ZIP › SN/C/3.jpg]

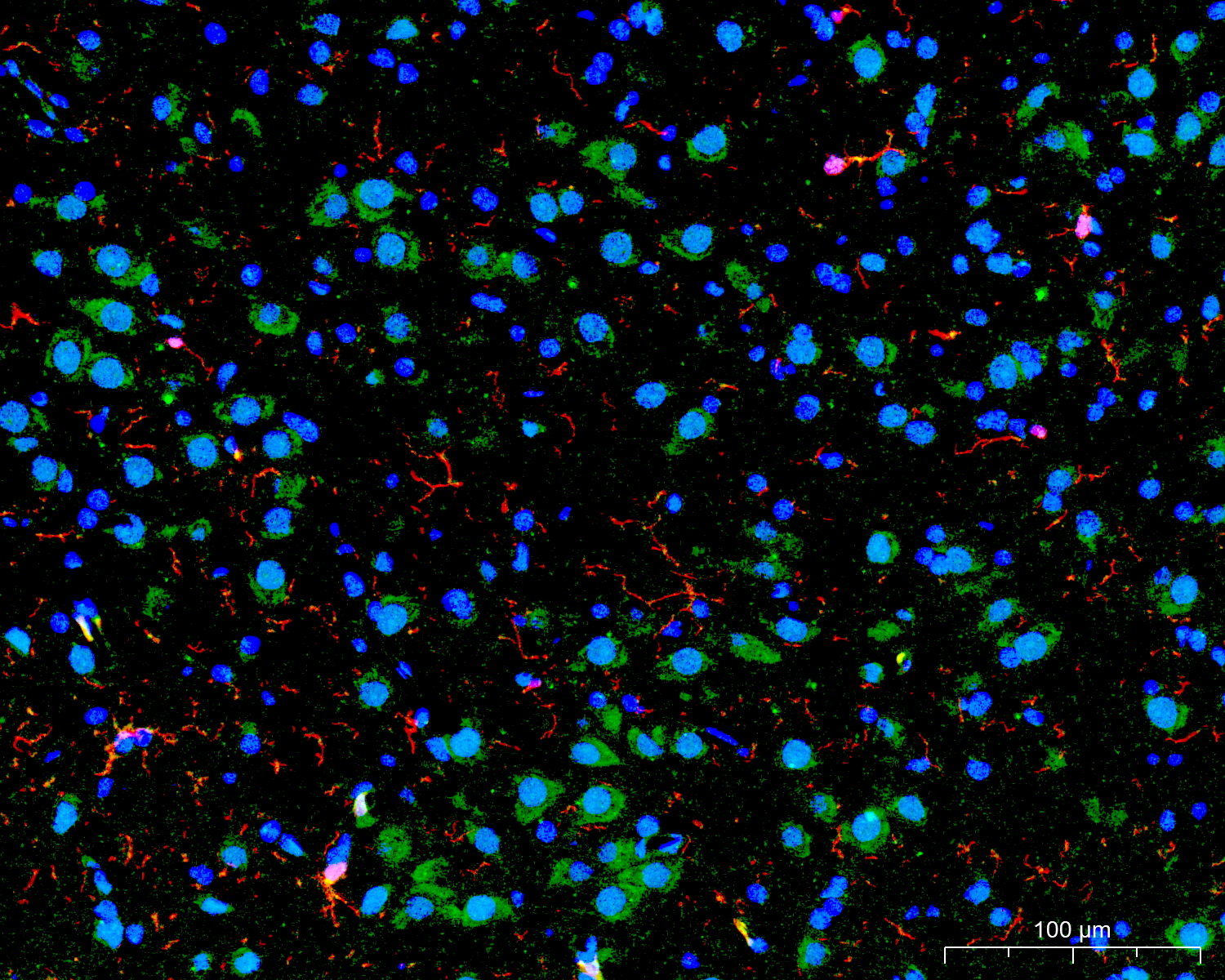

Supplement: Supplementary file 12 [file Data_Sheet_12.ZIP › SN/C/4.jpg]

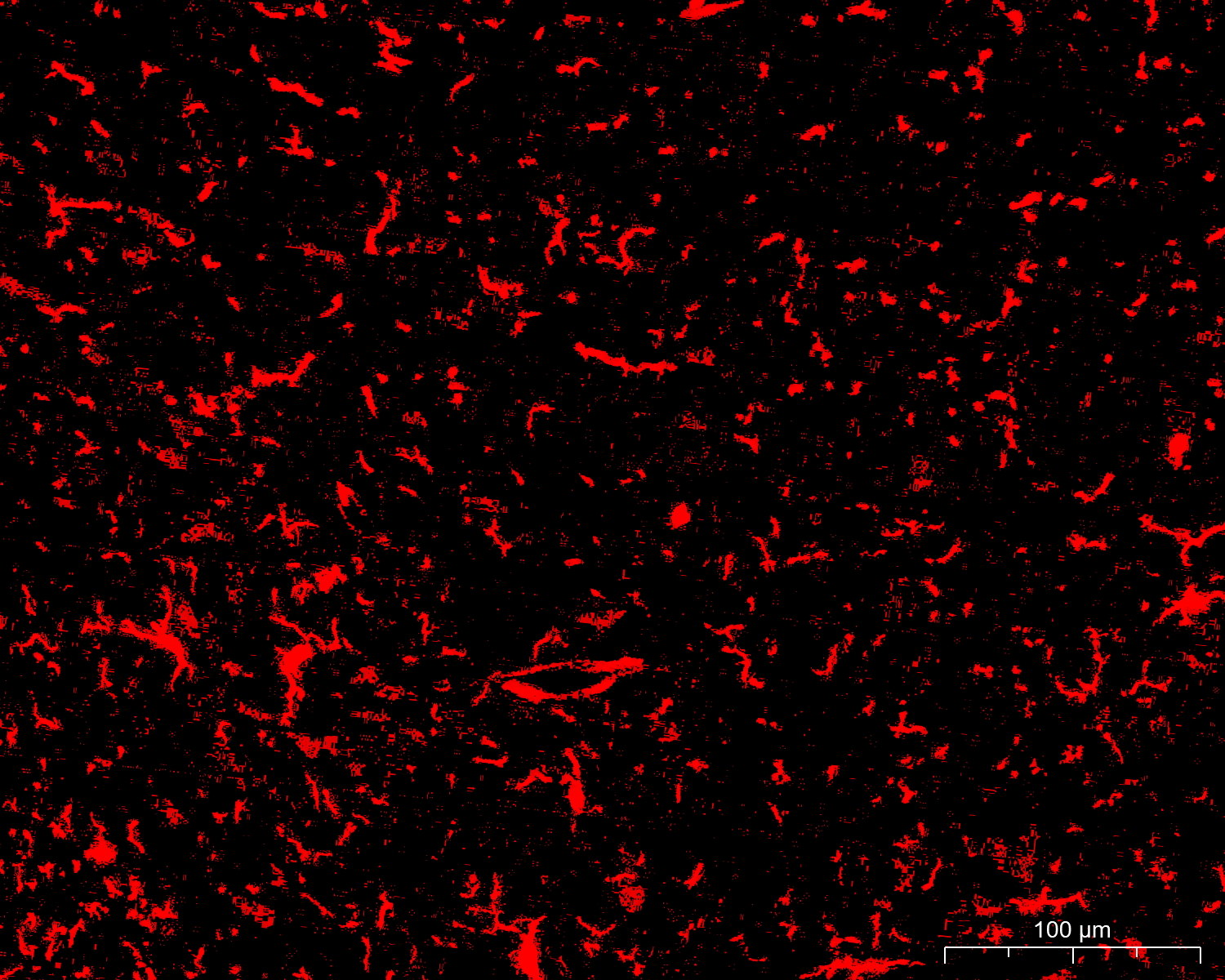

Supplement: Supplementary file 12 [file Data_Sheet_12.ZIP › SN/M/1.jpg]

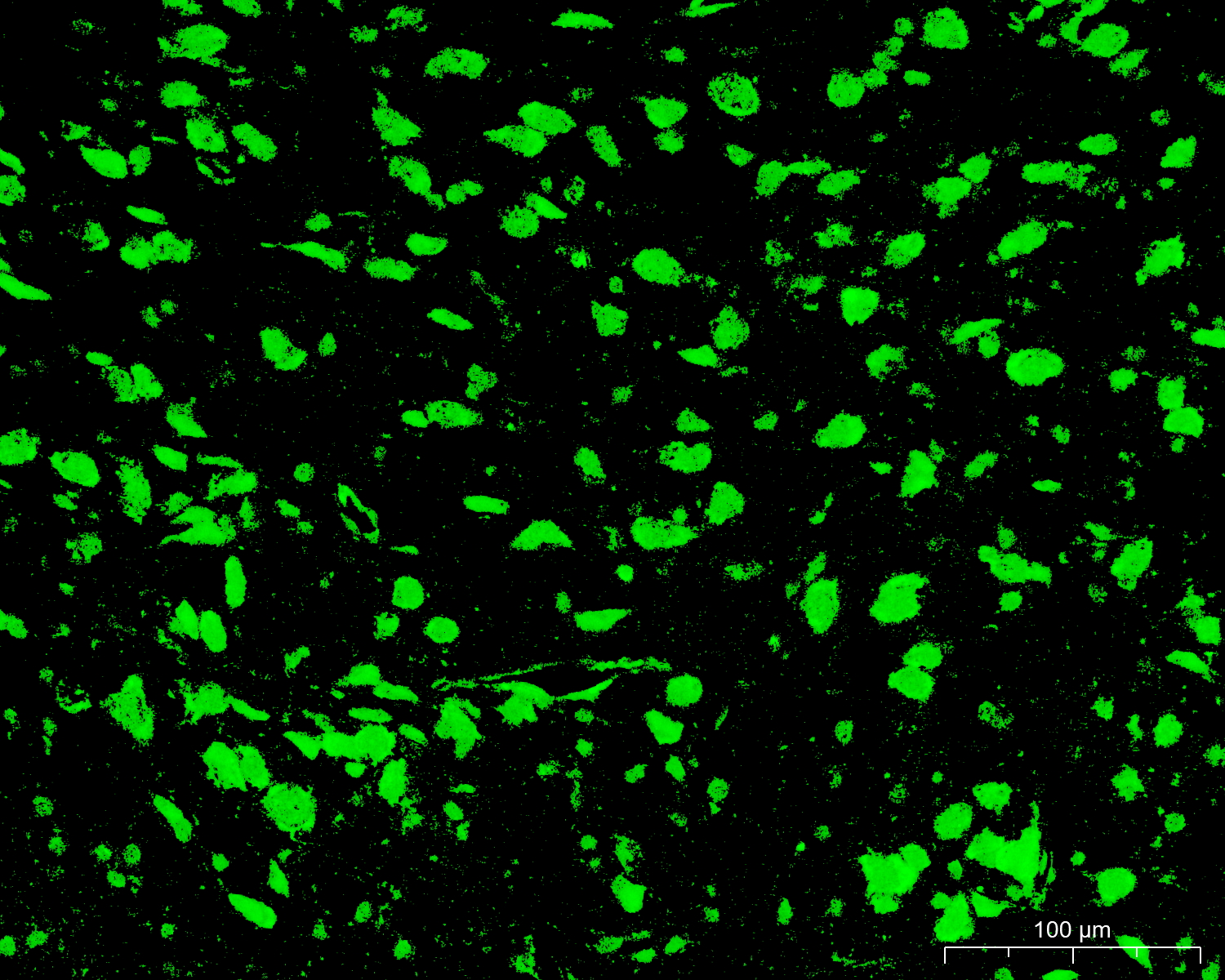

Supplement: Supplementary file 12 [file Data_Sheet_12.ZIP › SN/M/2.jpg]

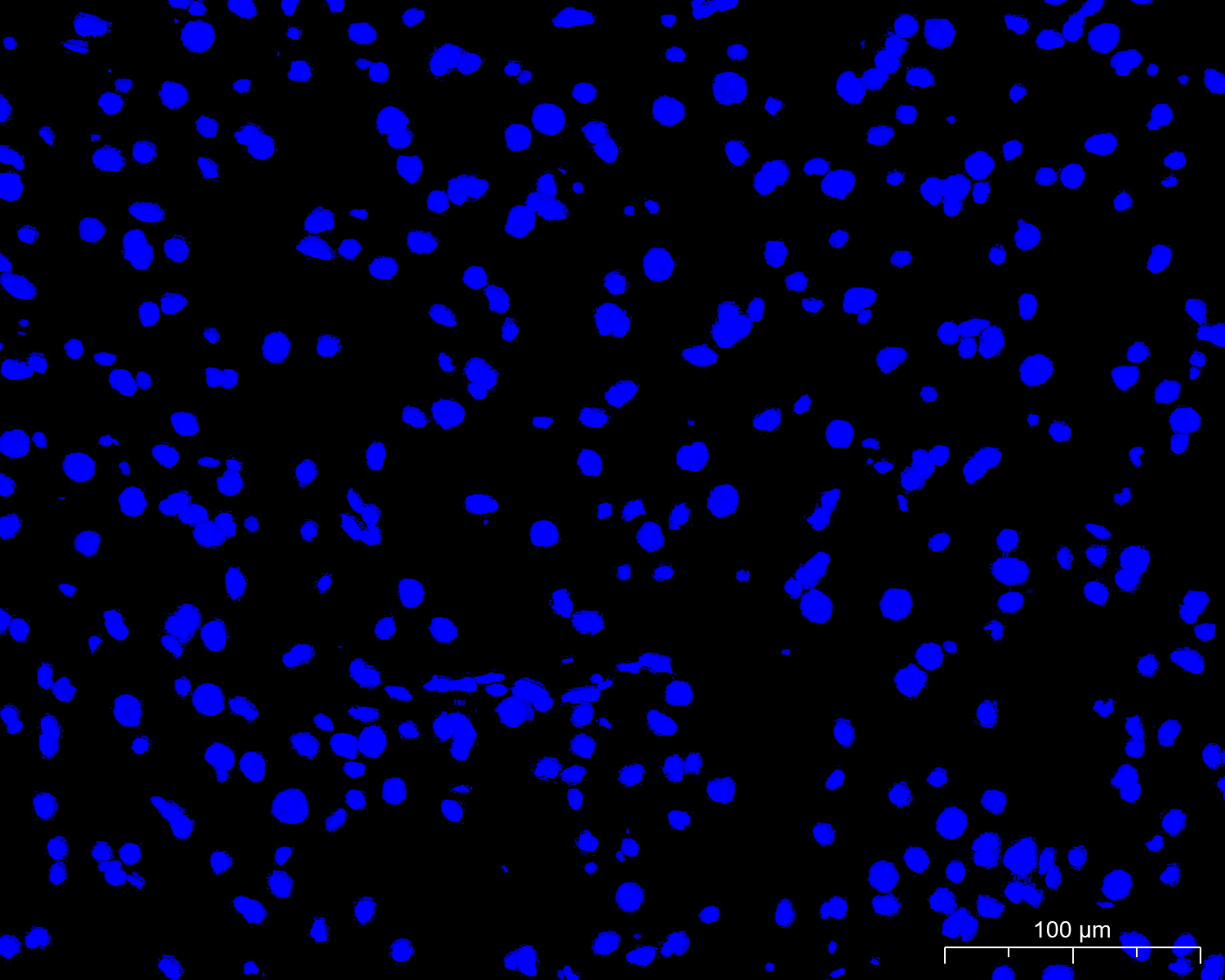

Supplement: Supplementary file 12 [file Data_Sheet_12.ZIP › SN/M/3.jpg]

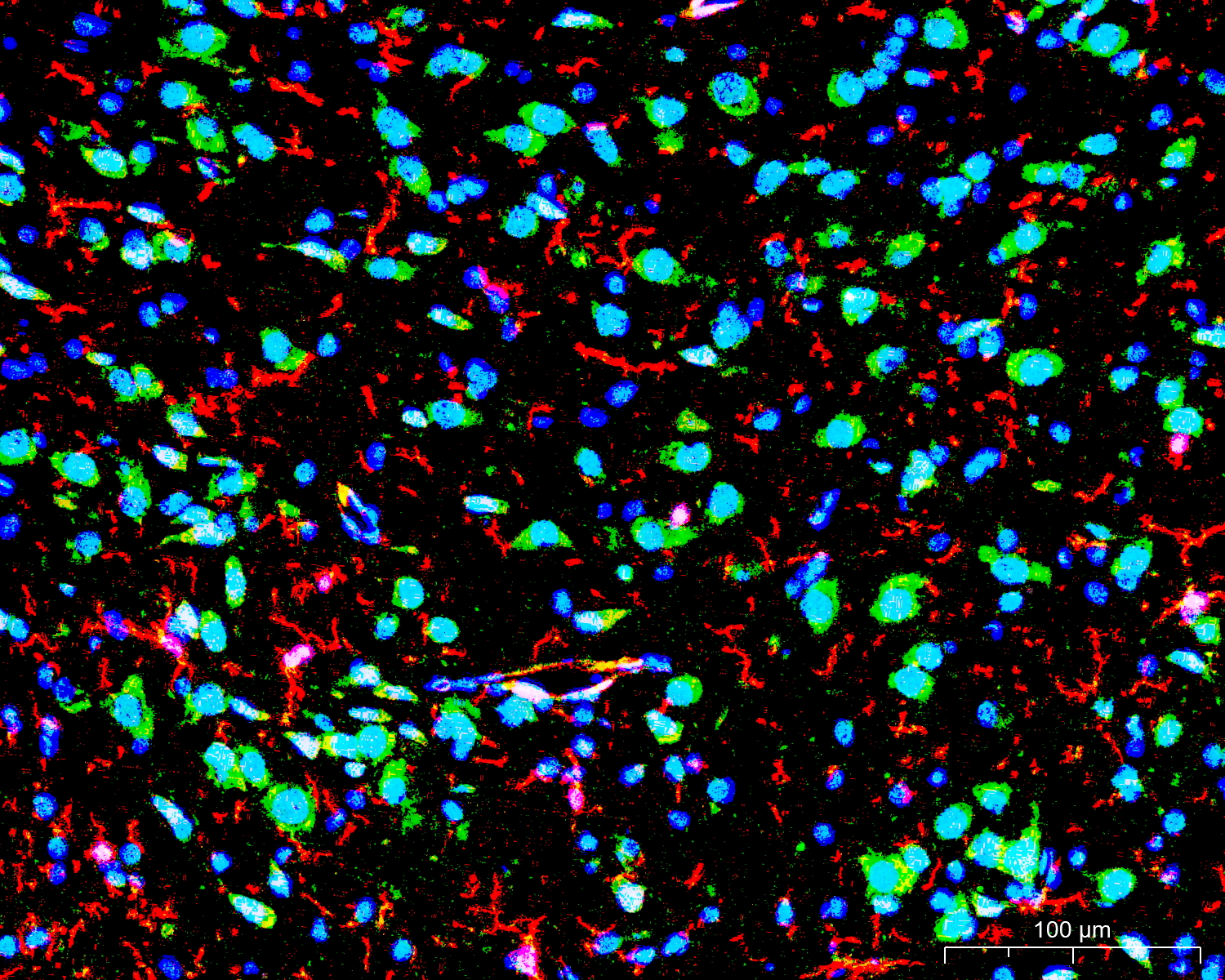

Supplement: Supplementary file 12 [file Data_Sheet_12.ZIP › SN/M/4.jpg]

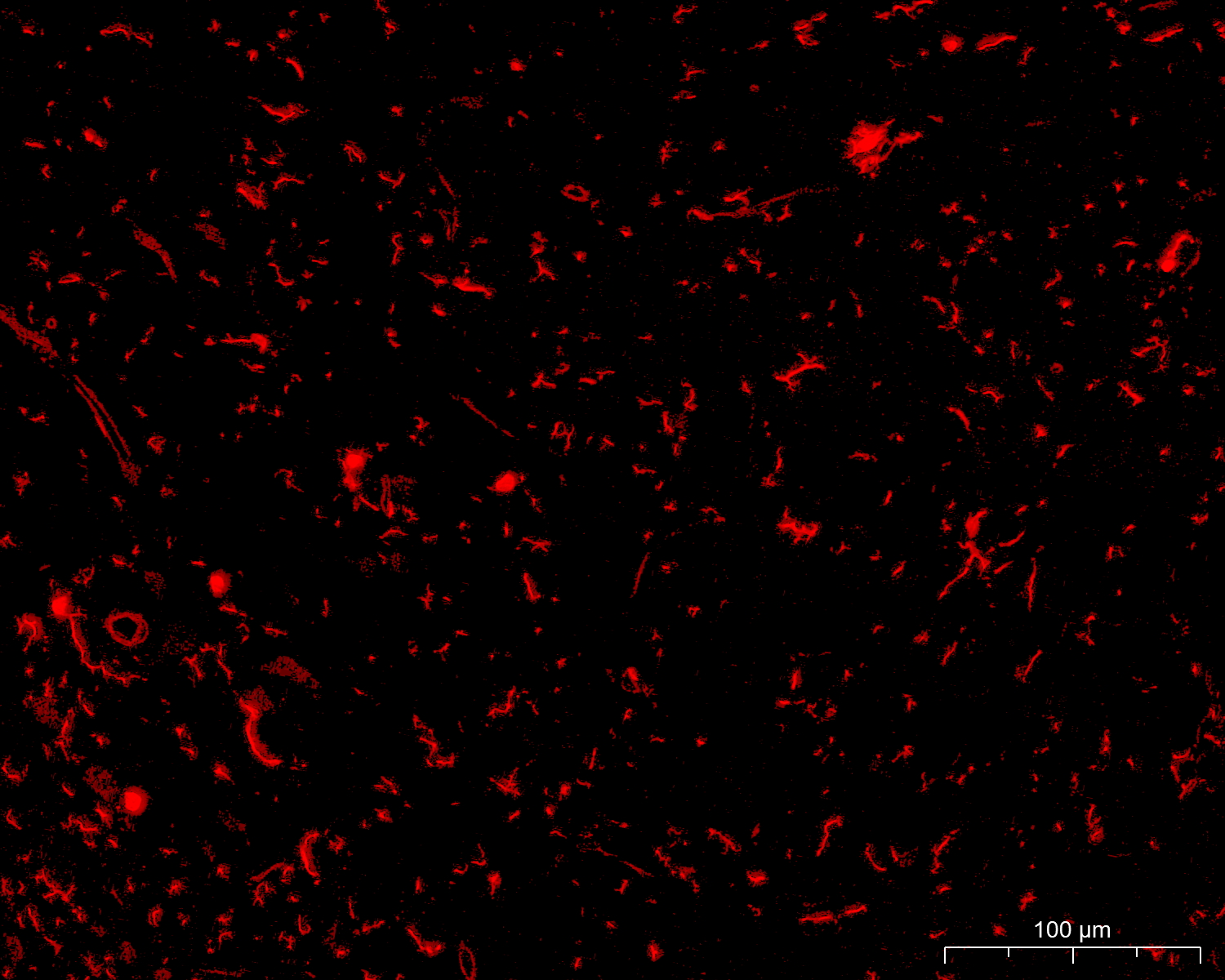

Supplement: Supplementary file 12 [file Data_Sheet_12.ZIP › SN/MR/1.jpg]

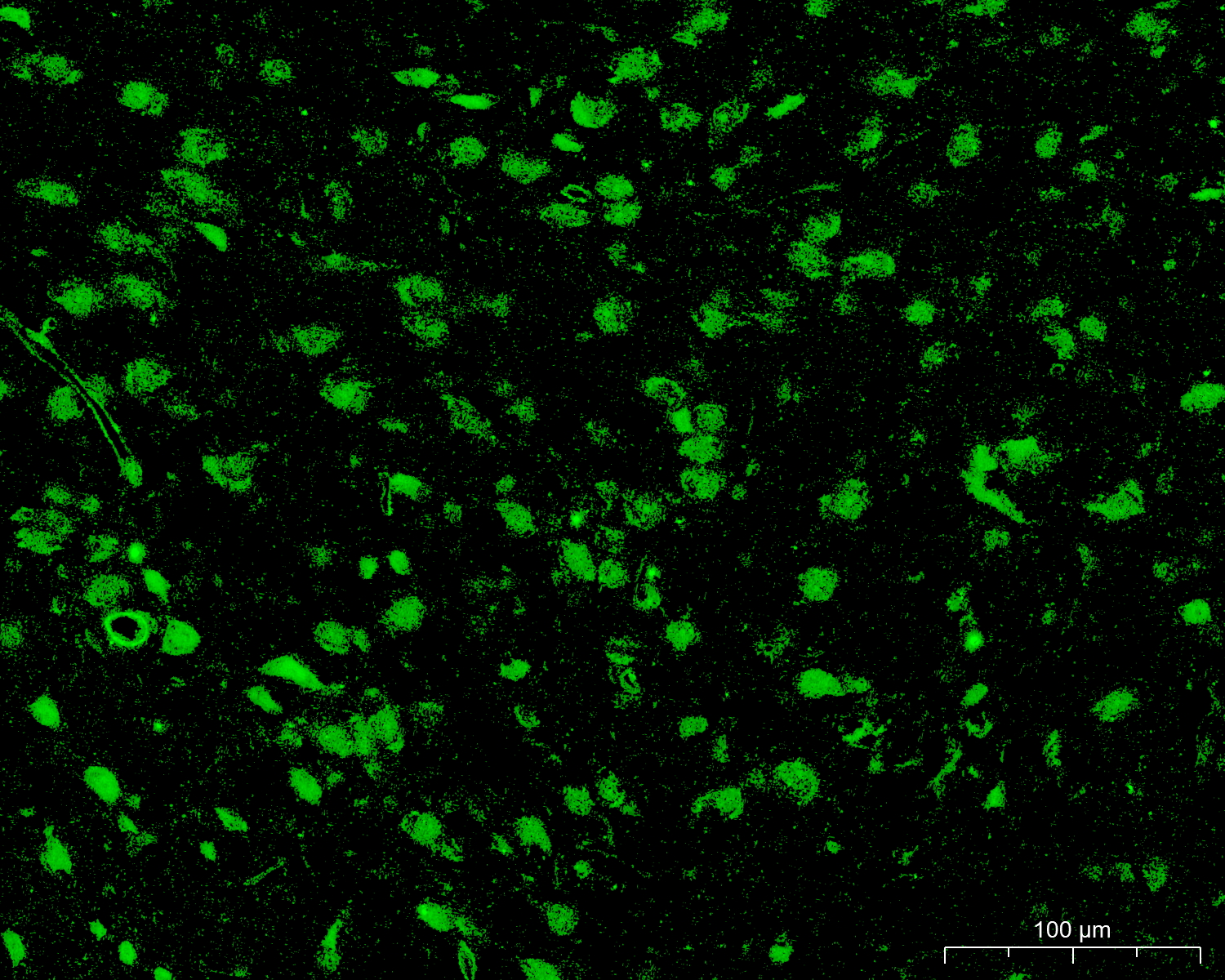

Supplement: Supplementary file 12 [file Data_Sheet_12.ZIP › SN/MR/2.jpg]

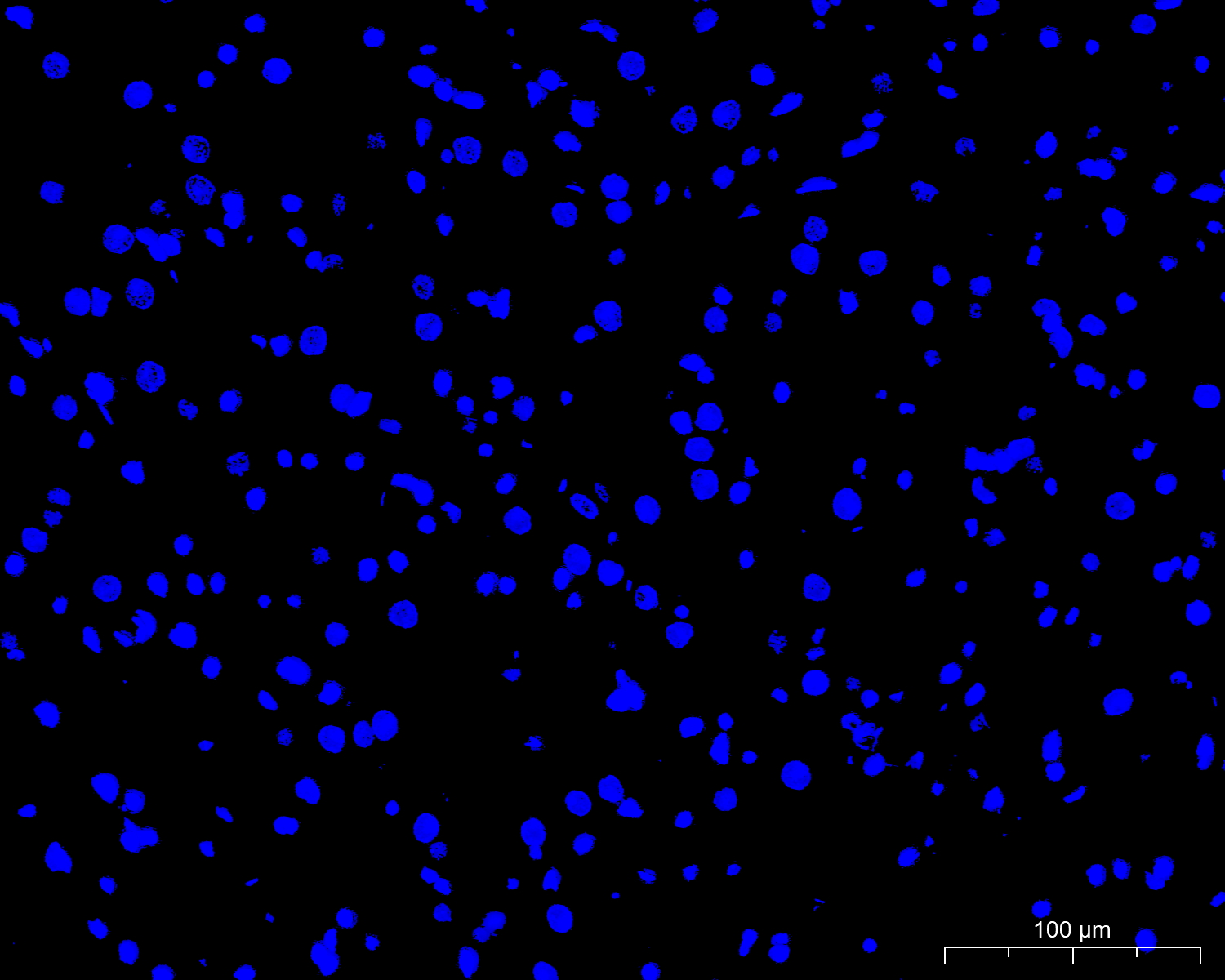

Supplement: Supplementary file 12 [file Data_Sheet_12.ZIP › SN/MR/3.jpg]

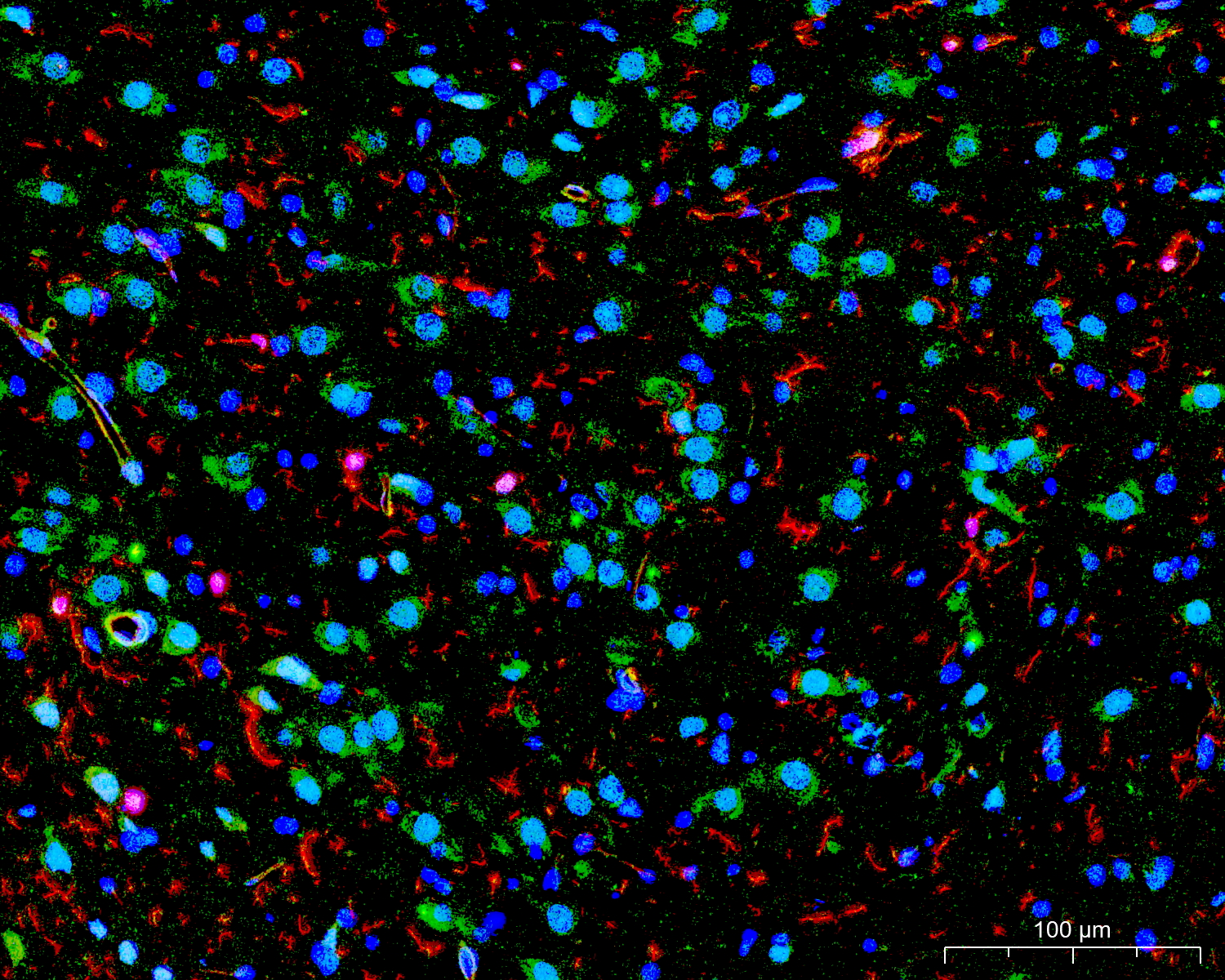

Supplement: Supplementary file 12 [file Data_Sheet_12.ZIP › SN/MR/4.jpg]

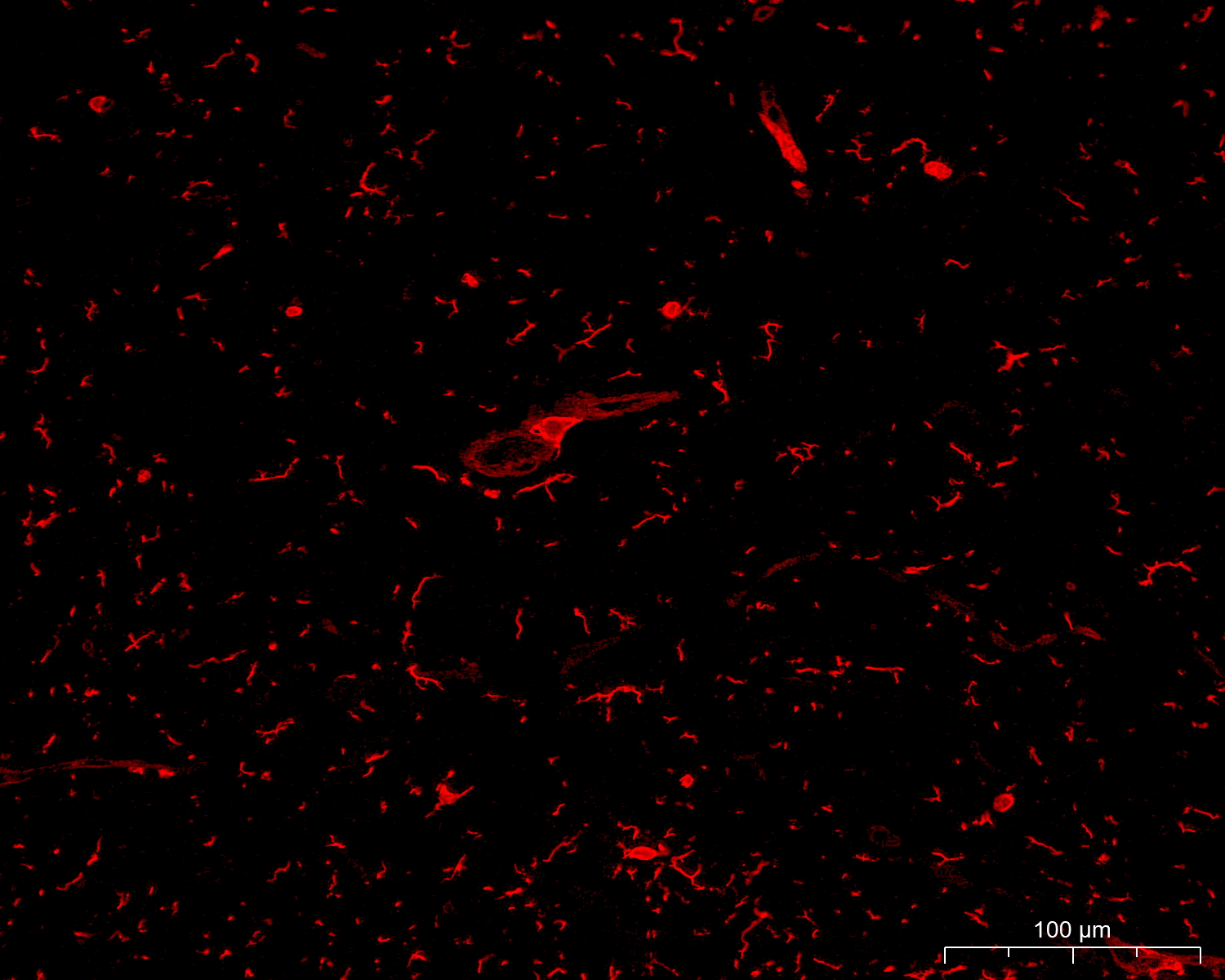

Supplement: Supplementary file 12 [file Data_Sheet_12.ZIP › SN/R/1.jpg]

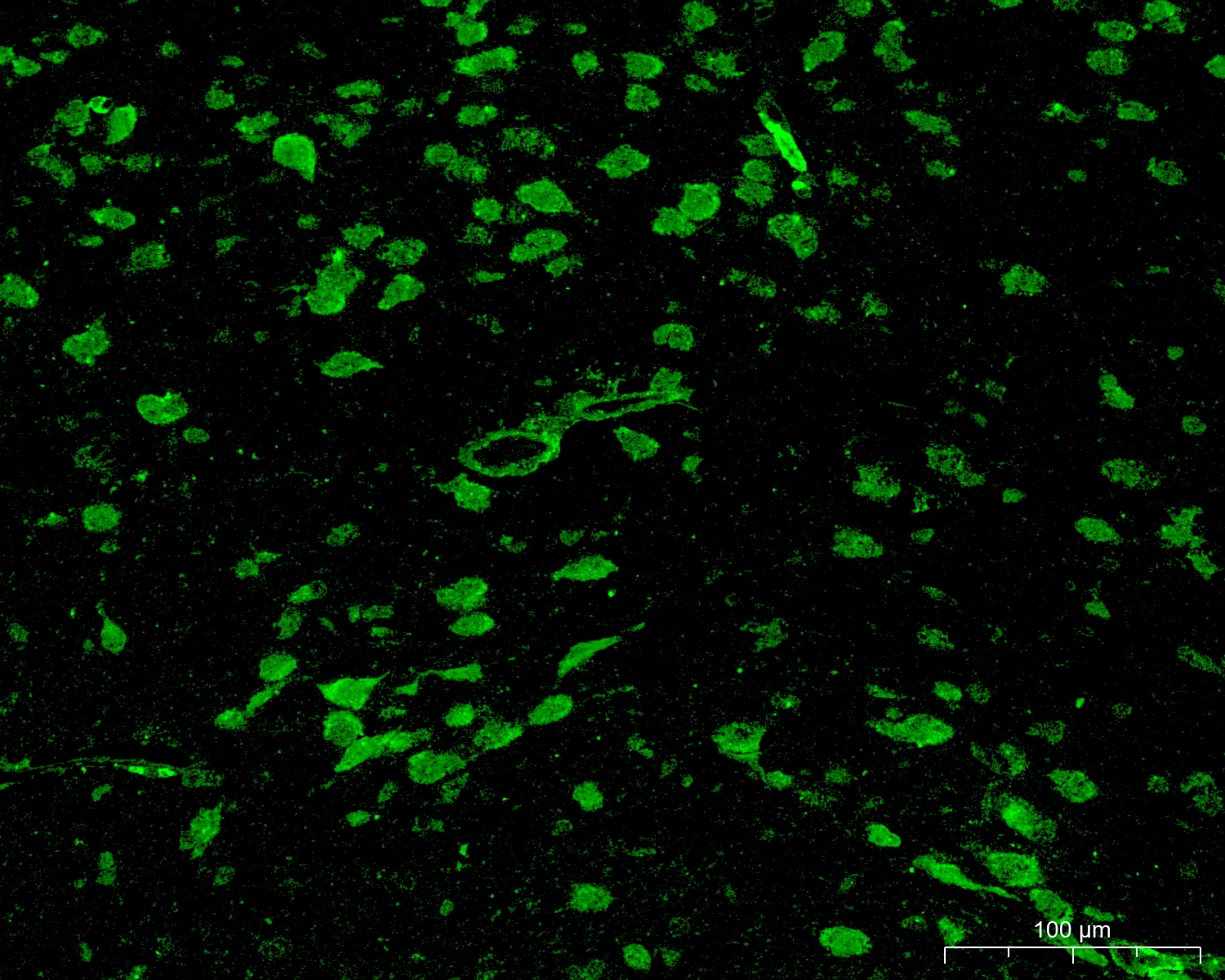

Supplement: Supplementary file 12 [file Data_Sheet_12.ZIP › SN/R/2.jpg]

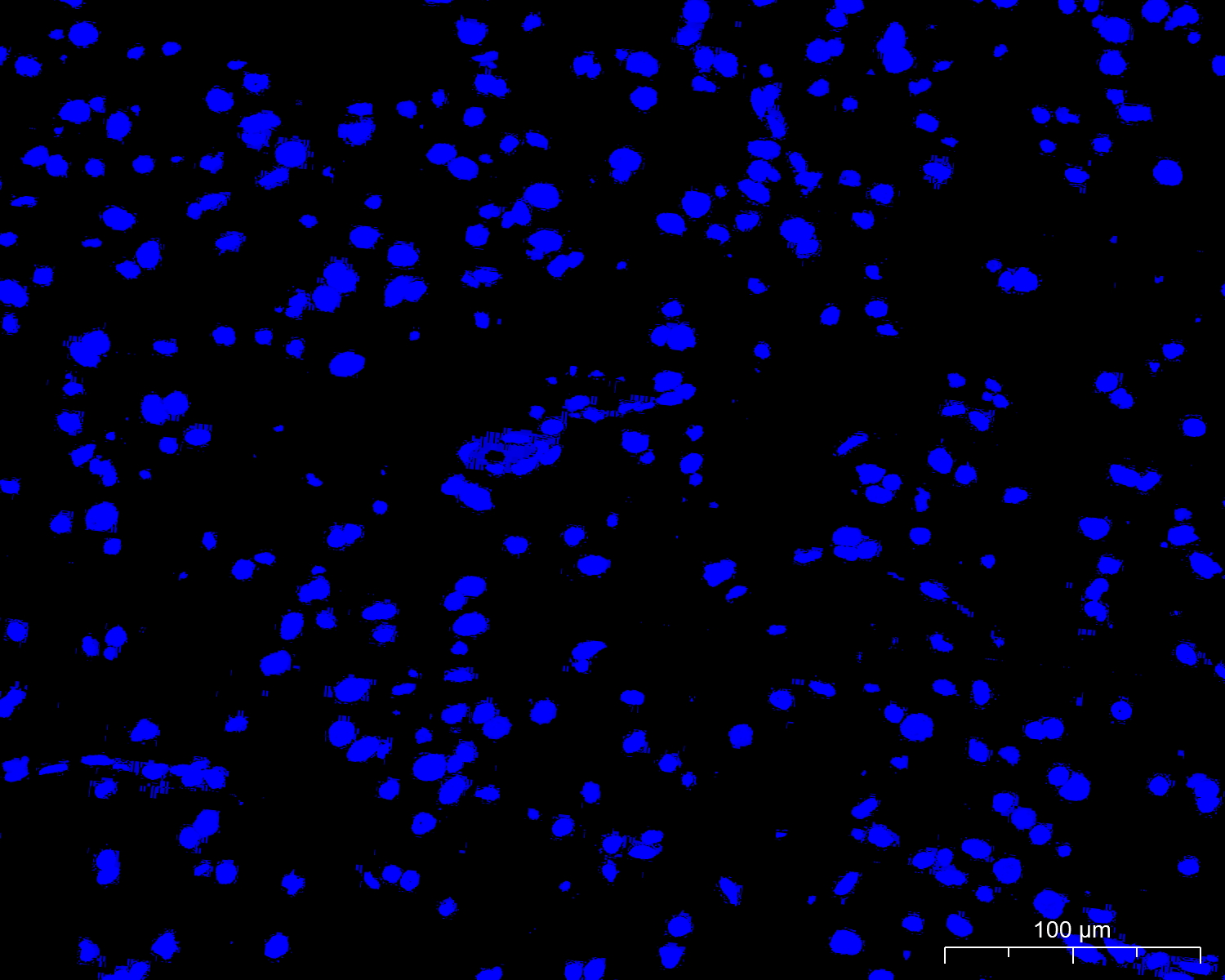

Supplement: Supplementary file 12 [file Data_Sheet_12.ZIP › SN/R/3.jpg]

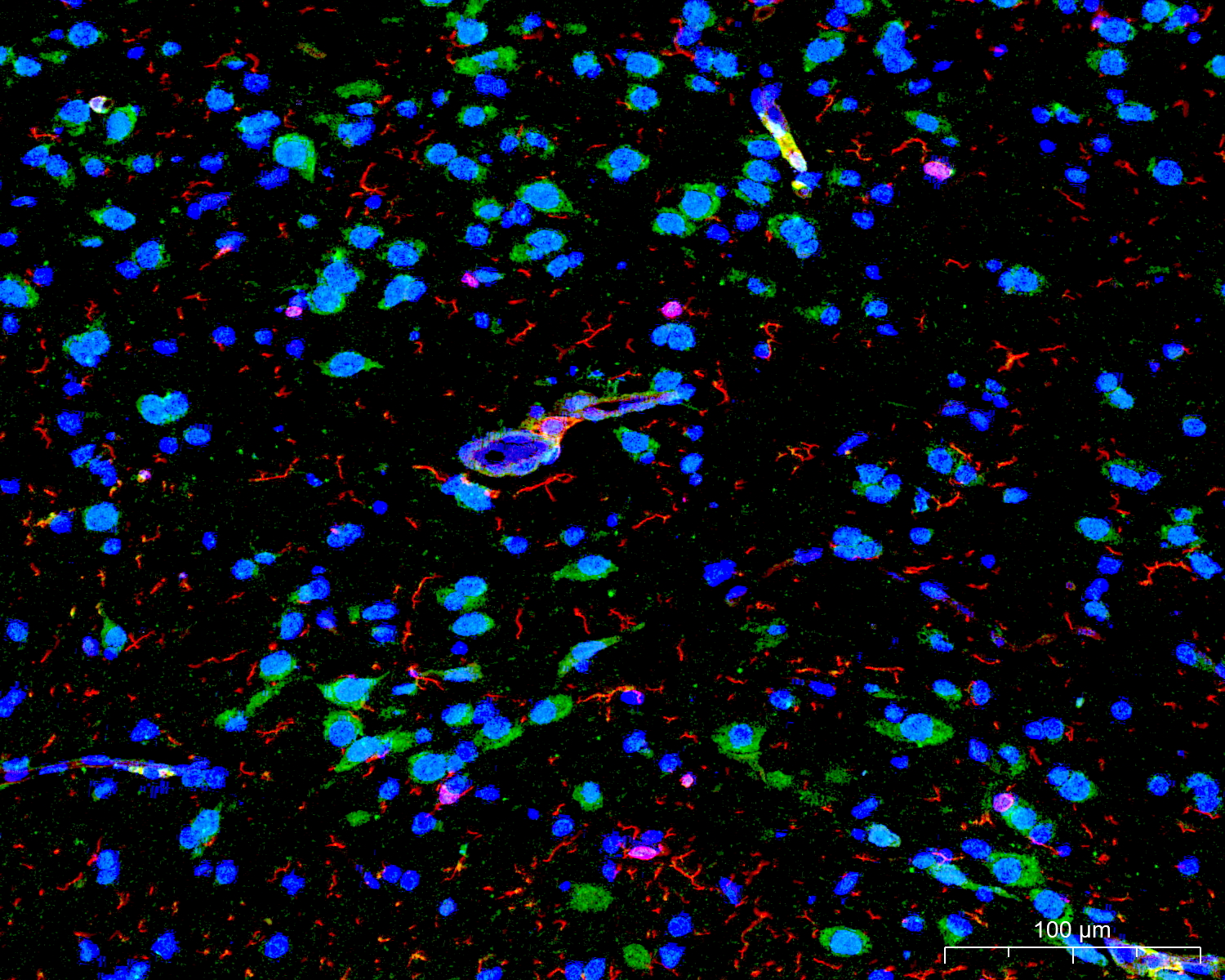

Supplement: Supplementary file 12 [file Data_Sheet_12.ZIP › SN/R/4.jpg]

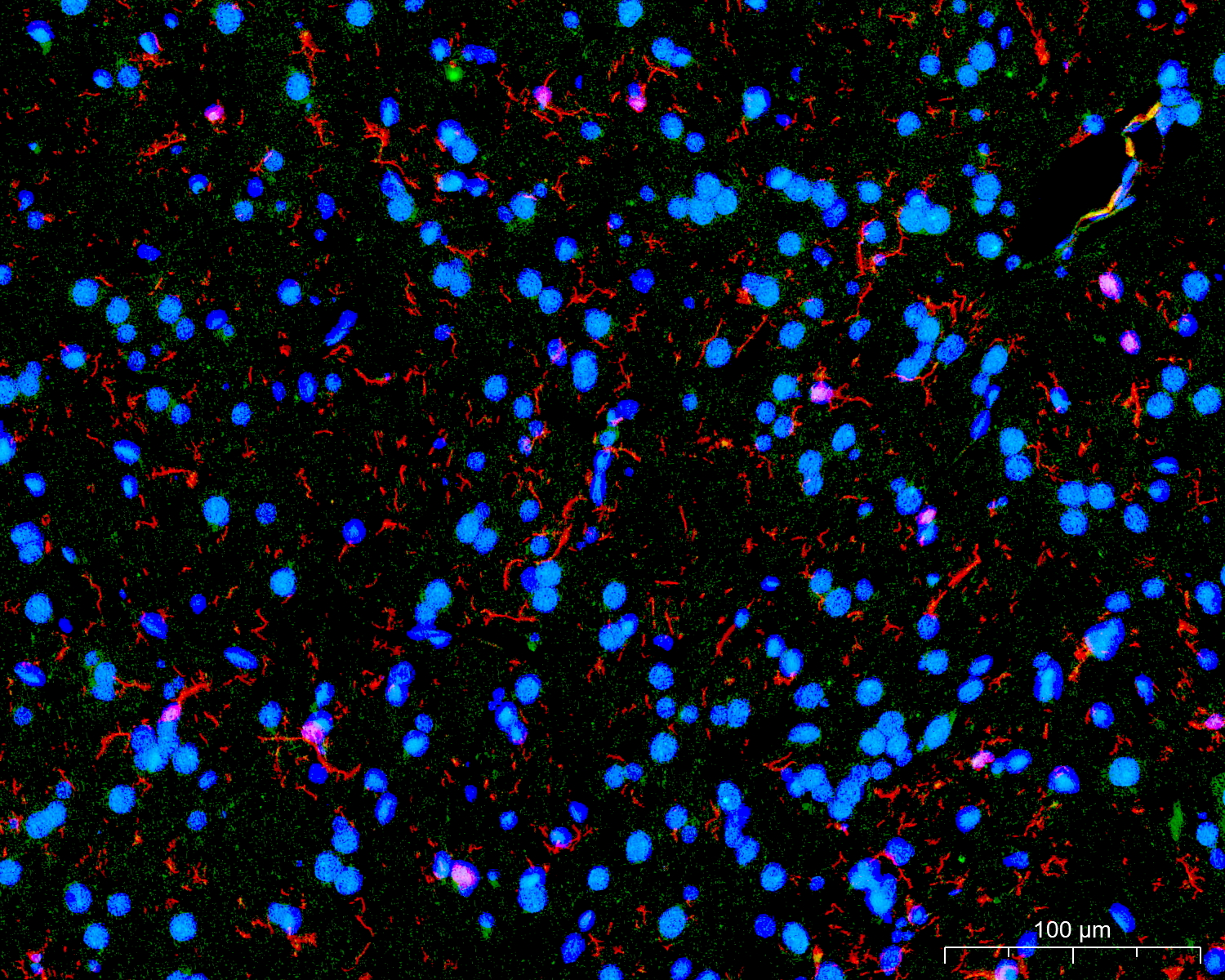

Supplement: Supplementary file 13 [file Data_Sheet_13.ZIP › ST/C/1.jpg]

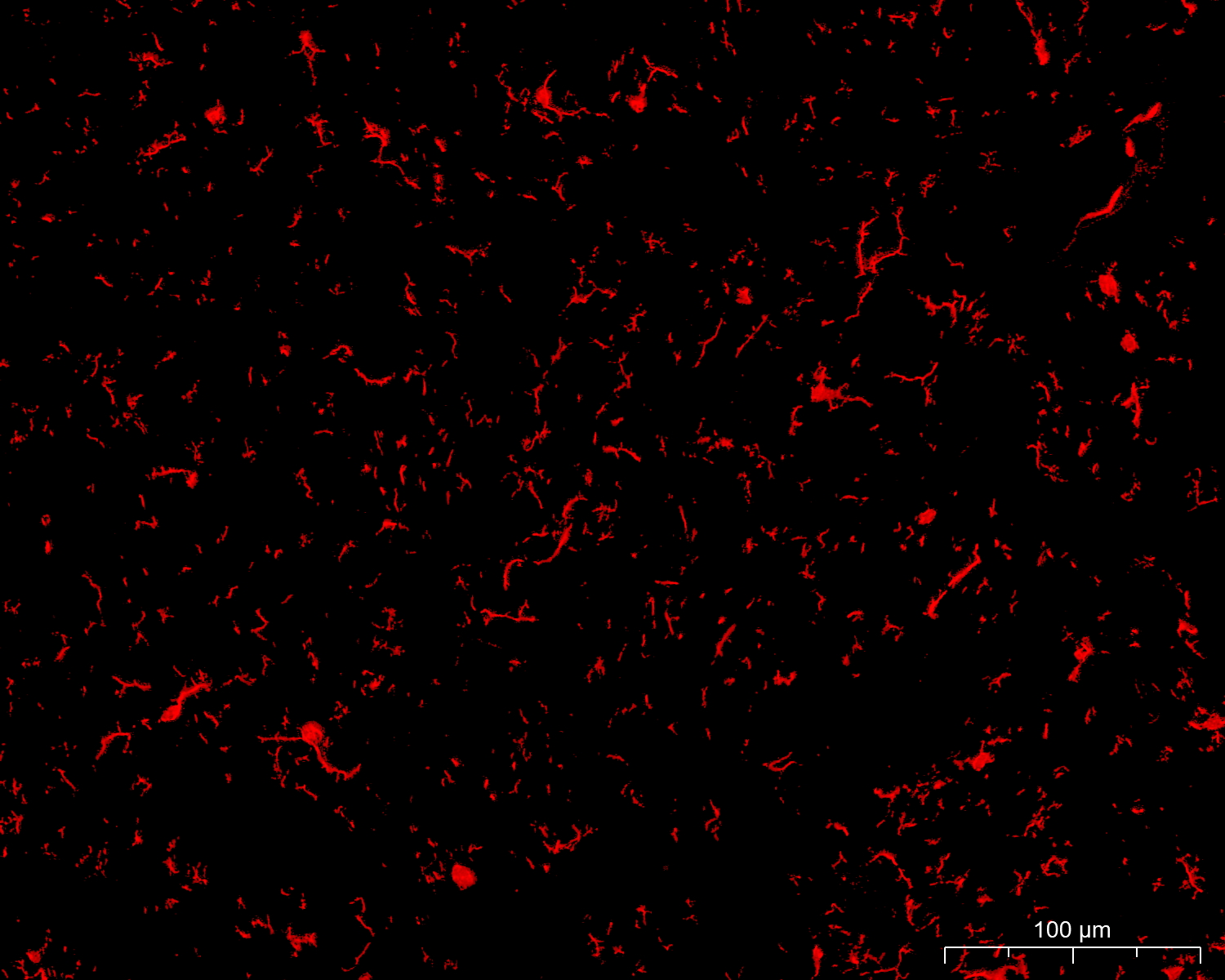

Supplement: Supplementary file 13 [file Data_Sheet_13.ZIP › ST/C/2.jpg]

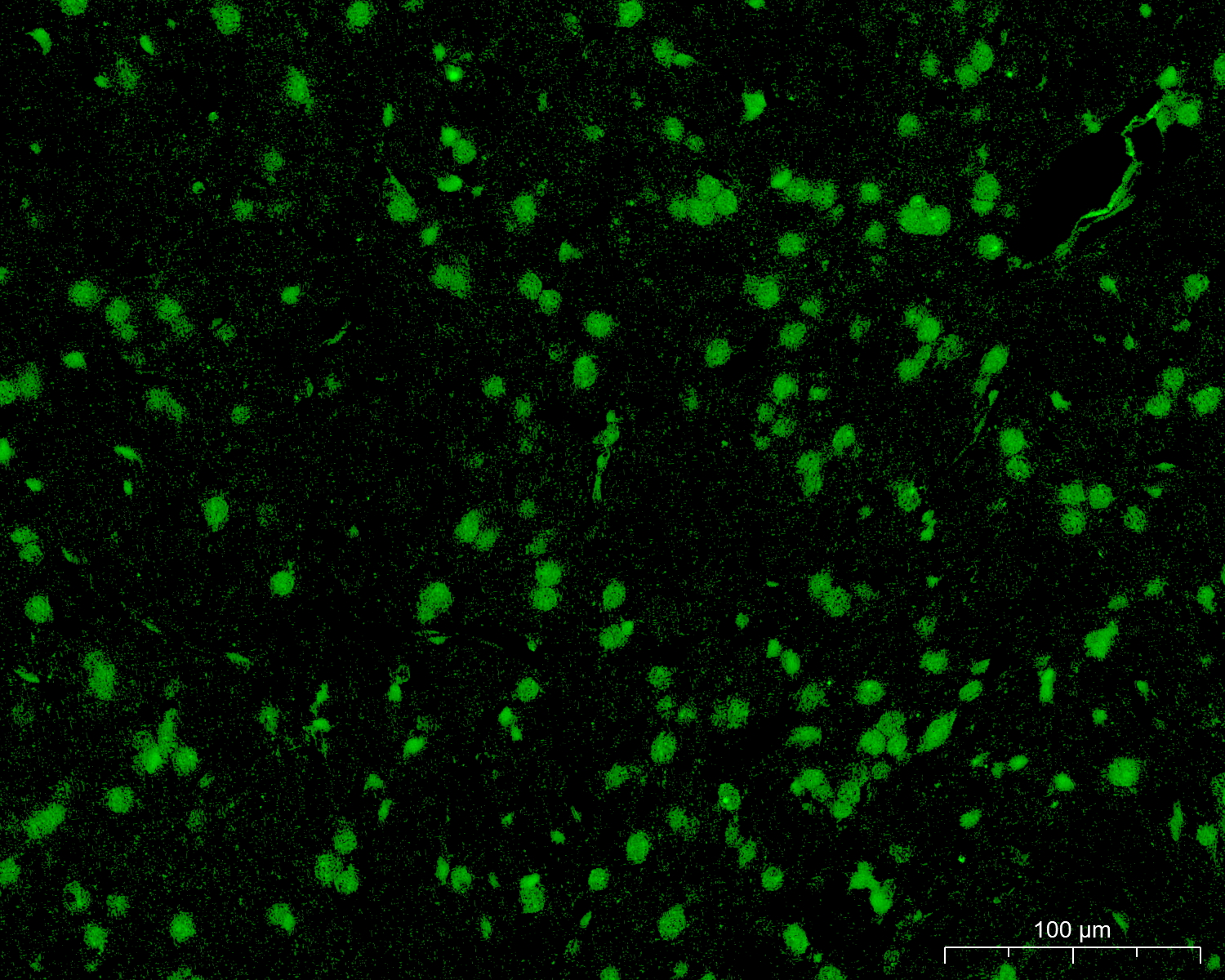

Supplement: Supplementary file 13 [file Data_Sheet_13.ZIP › ST/C/3.jpg]

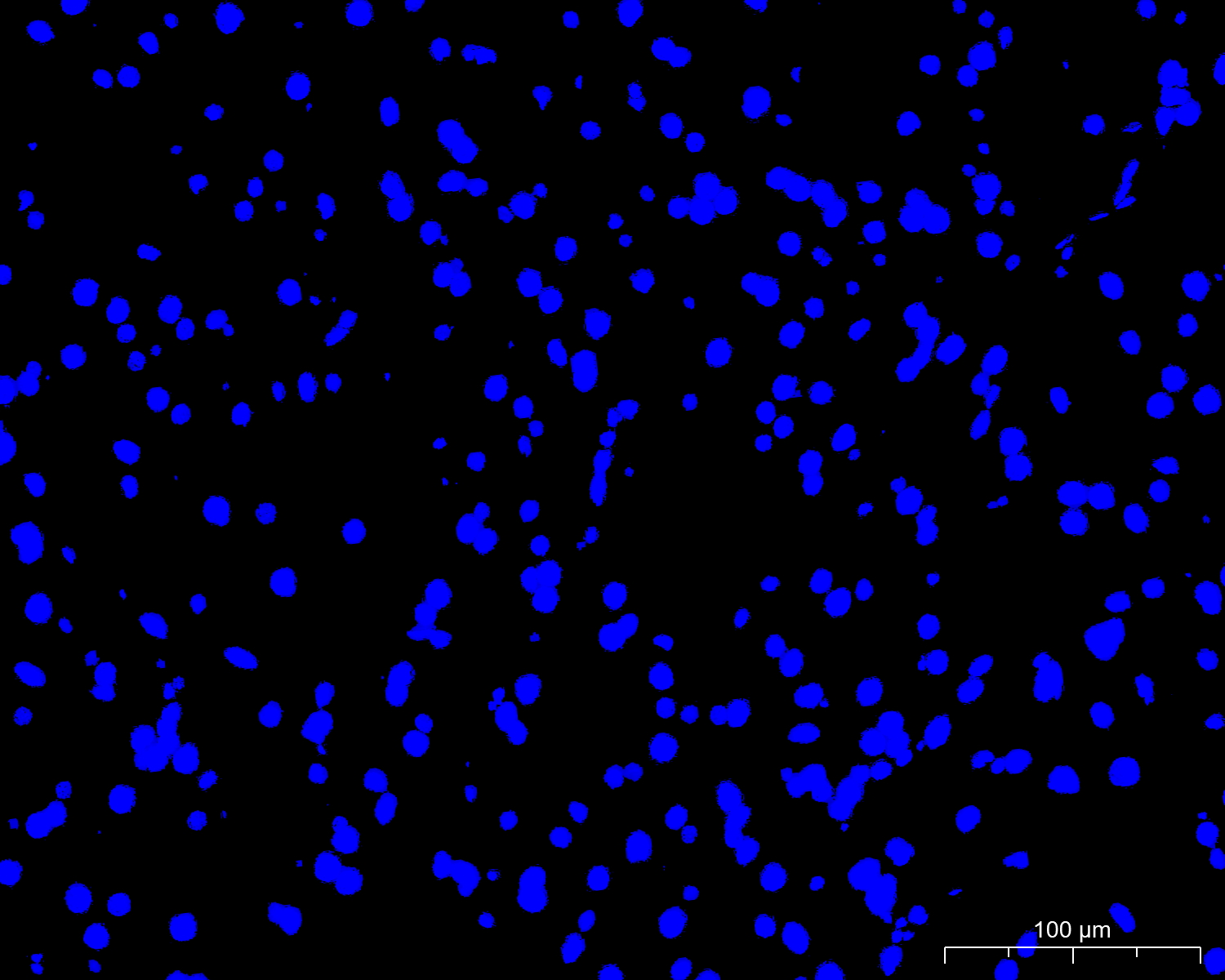

Supplement: Supplementary file 13 [file Data_Sheet_13.ZIP › ST/C/4.jpg]

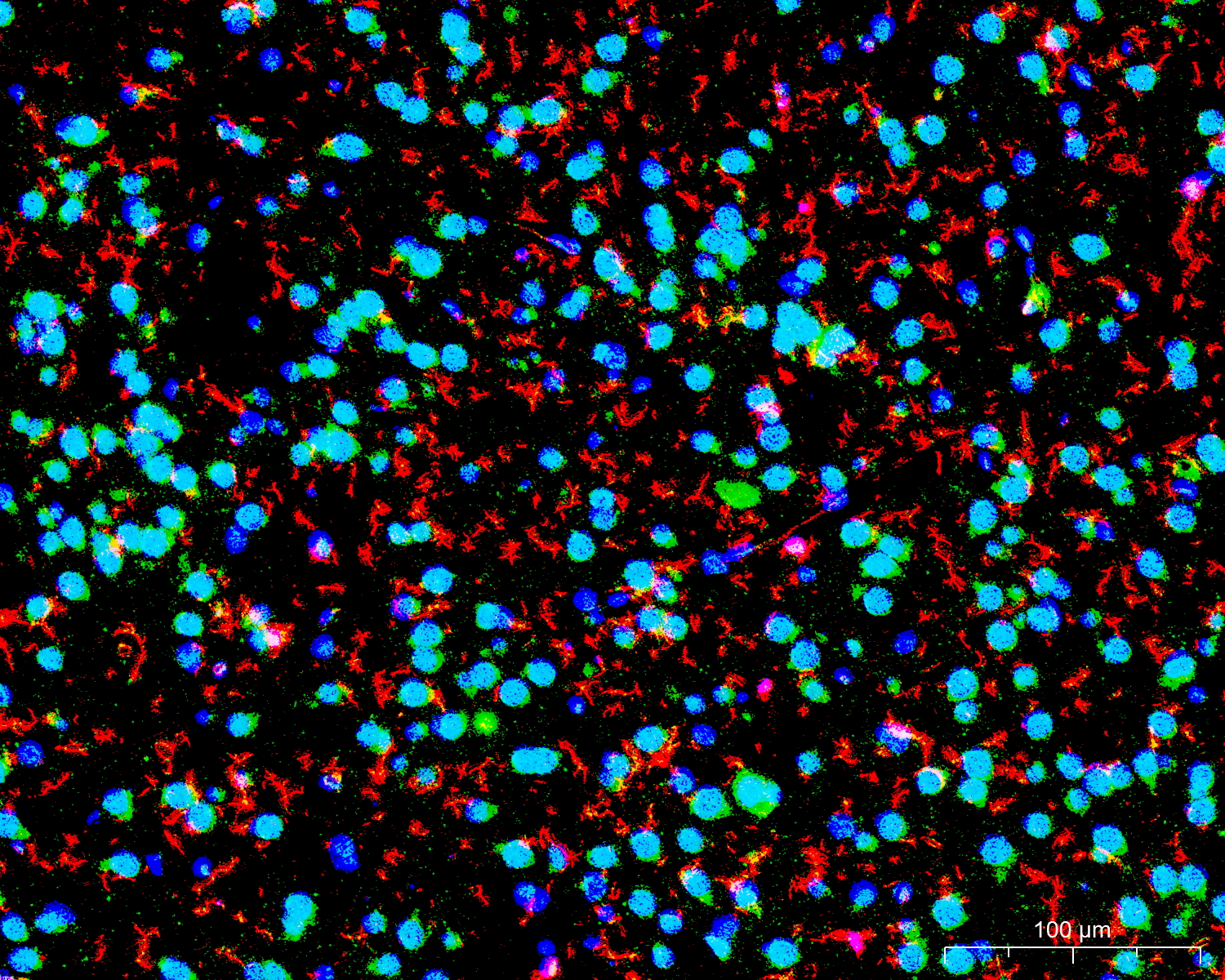

Supplement: Supplementary file 13 [file Data_Sheet_13.ZIP › ST/M/1.jpg]

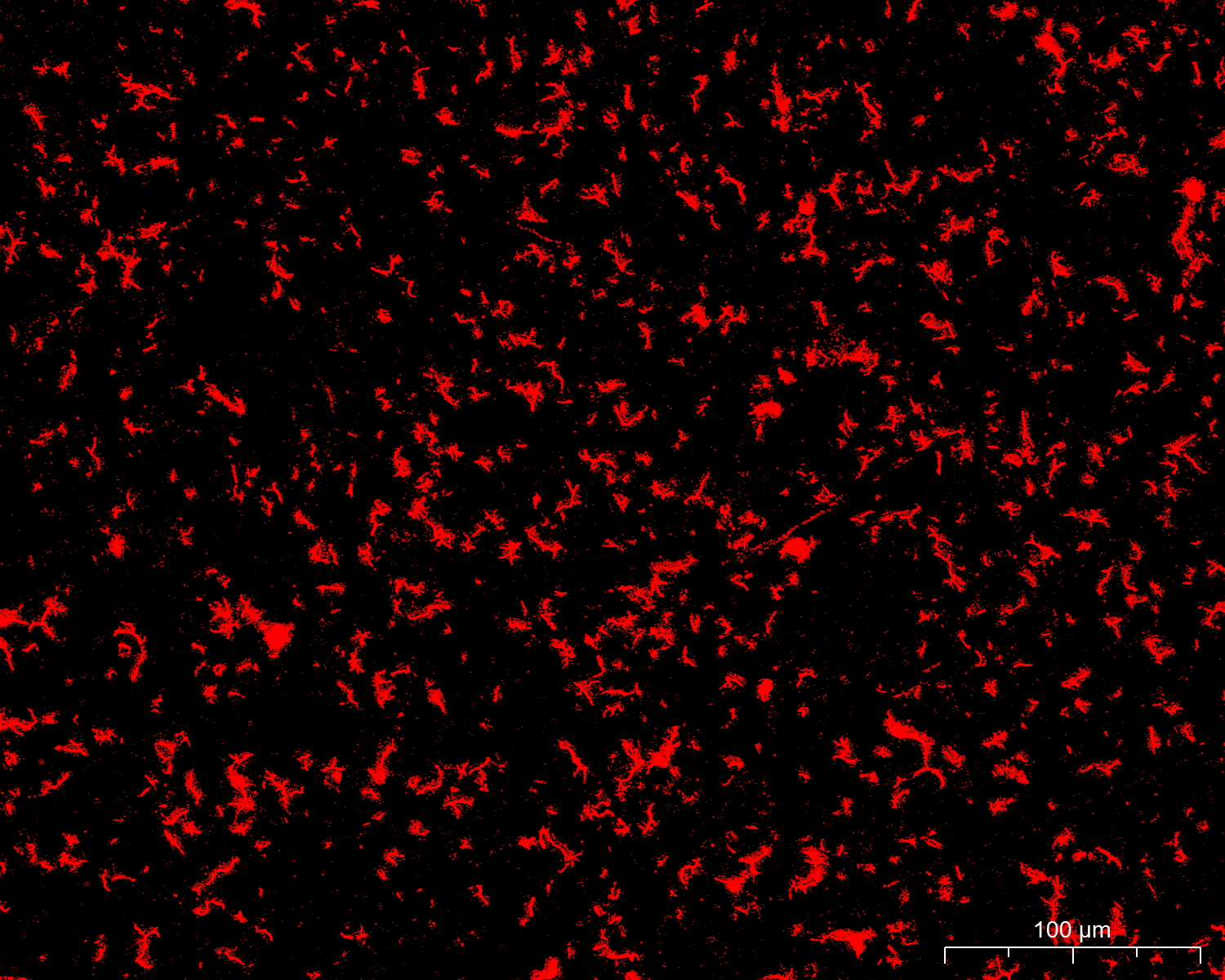

Supplement: Supplementary file 13 [file Data_Sheet_13.ZIP › ST/M/2.jpg]

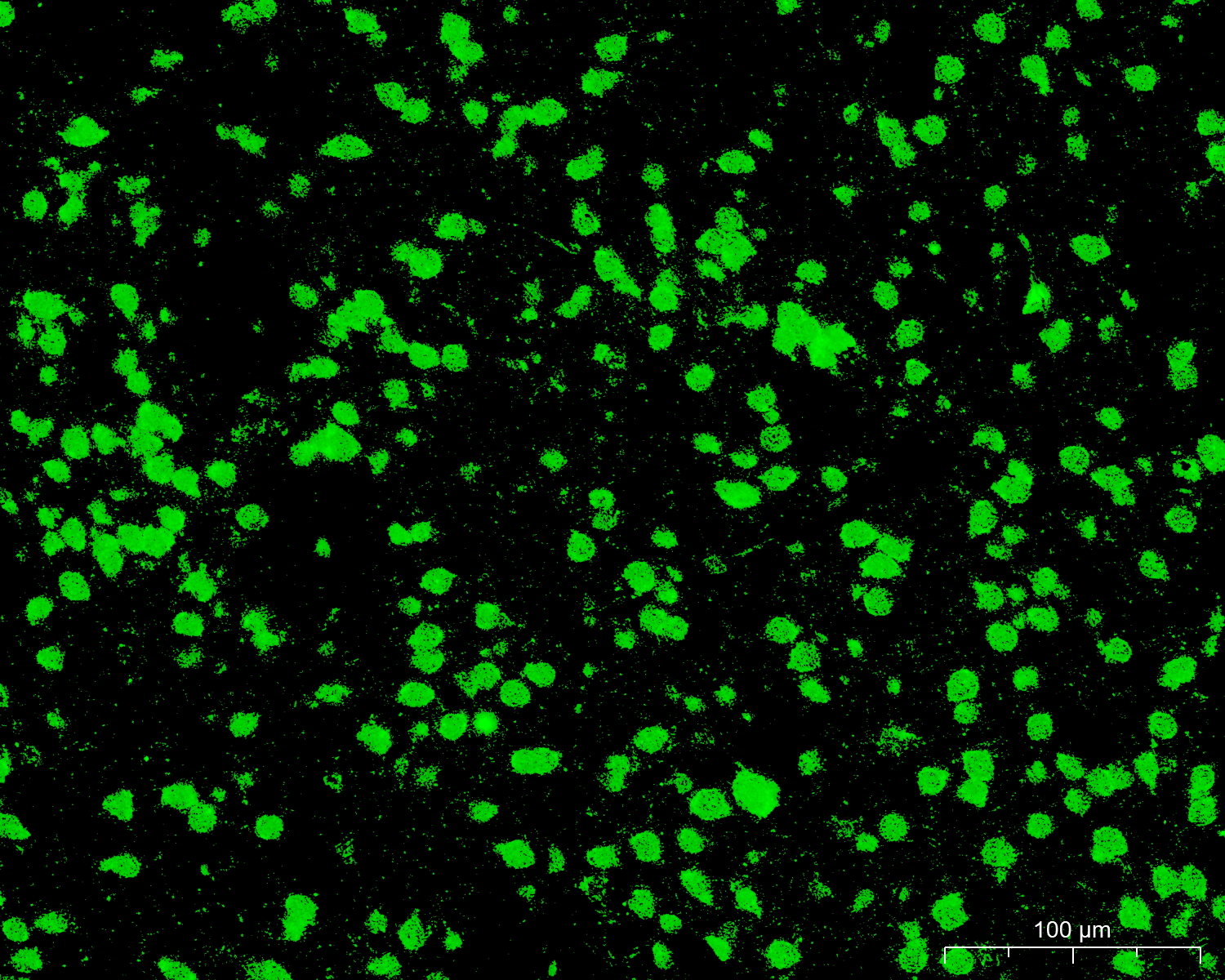

Supplement: Supplementary file 13 [file Data_Sheet_13.ZIP › ST/M/3.jpg]

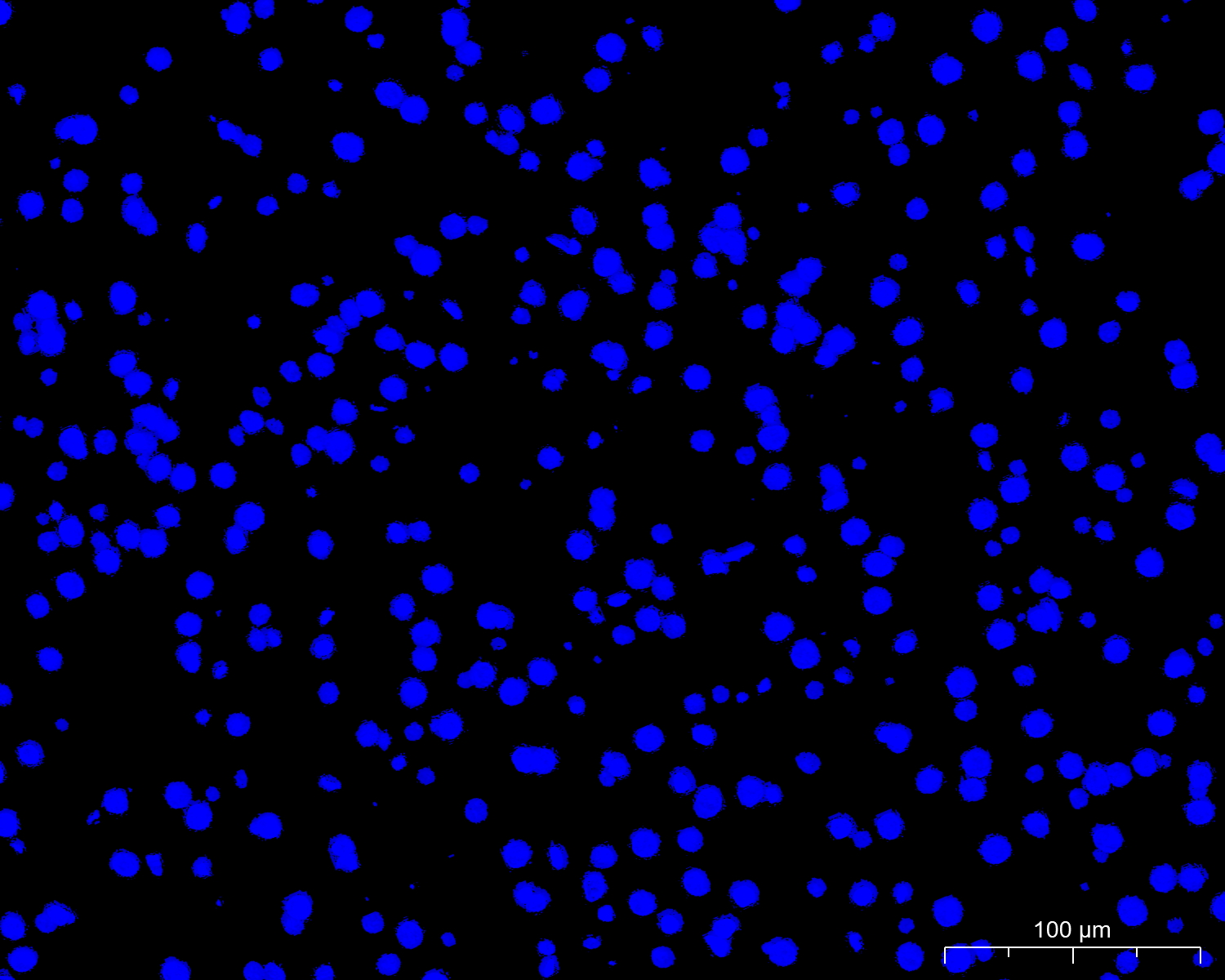

Supplement: Supplementary file 13 [file Data_Sheet_13.ZIP › ST/M/4.jpg]

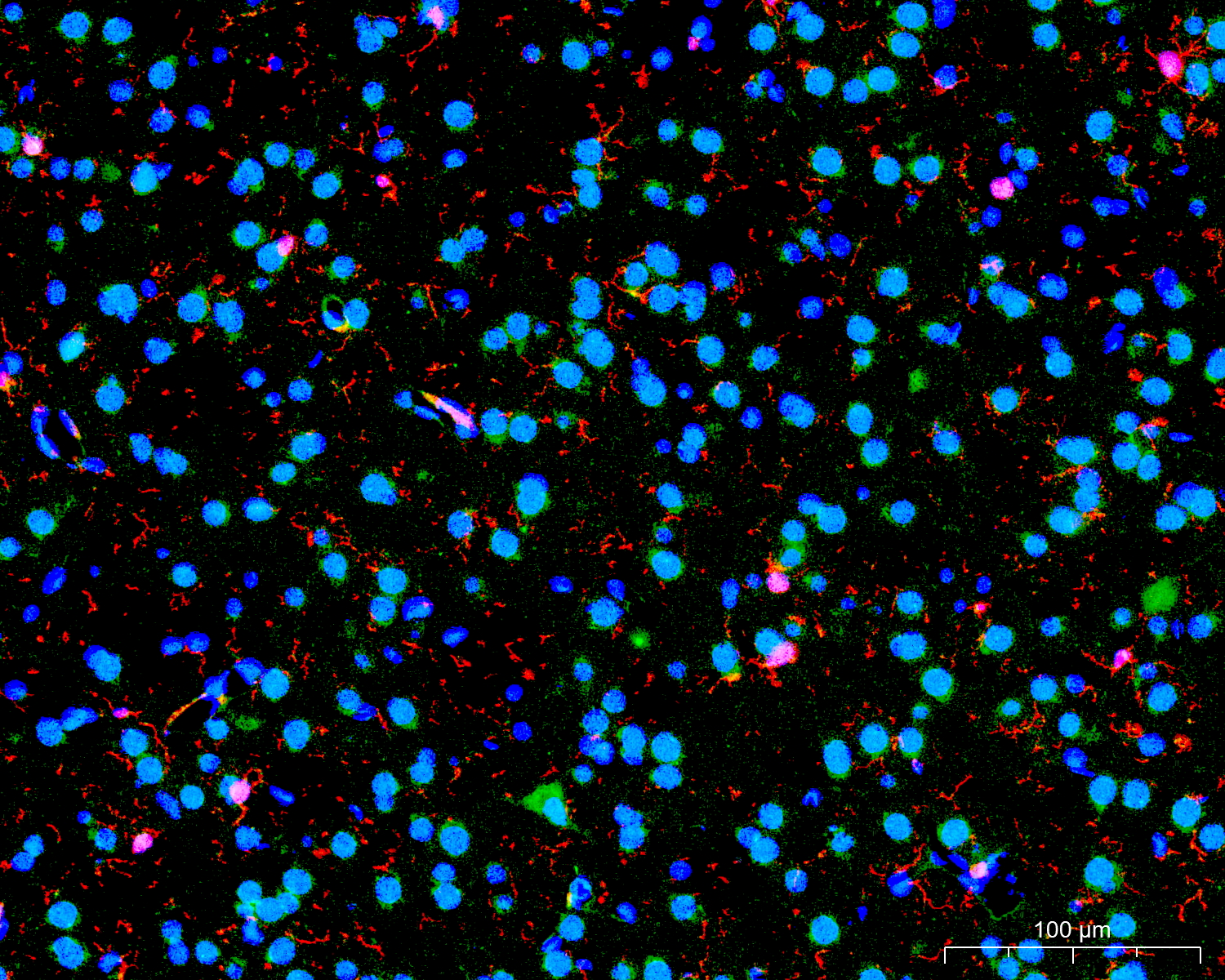

Supplement: Supplementary file 13 [file Data_Sheet_13.ZIP › ST/MR/1.jpg]

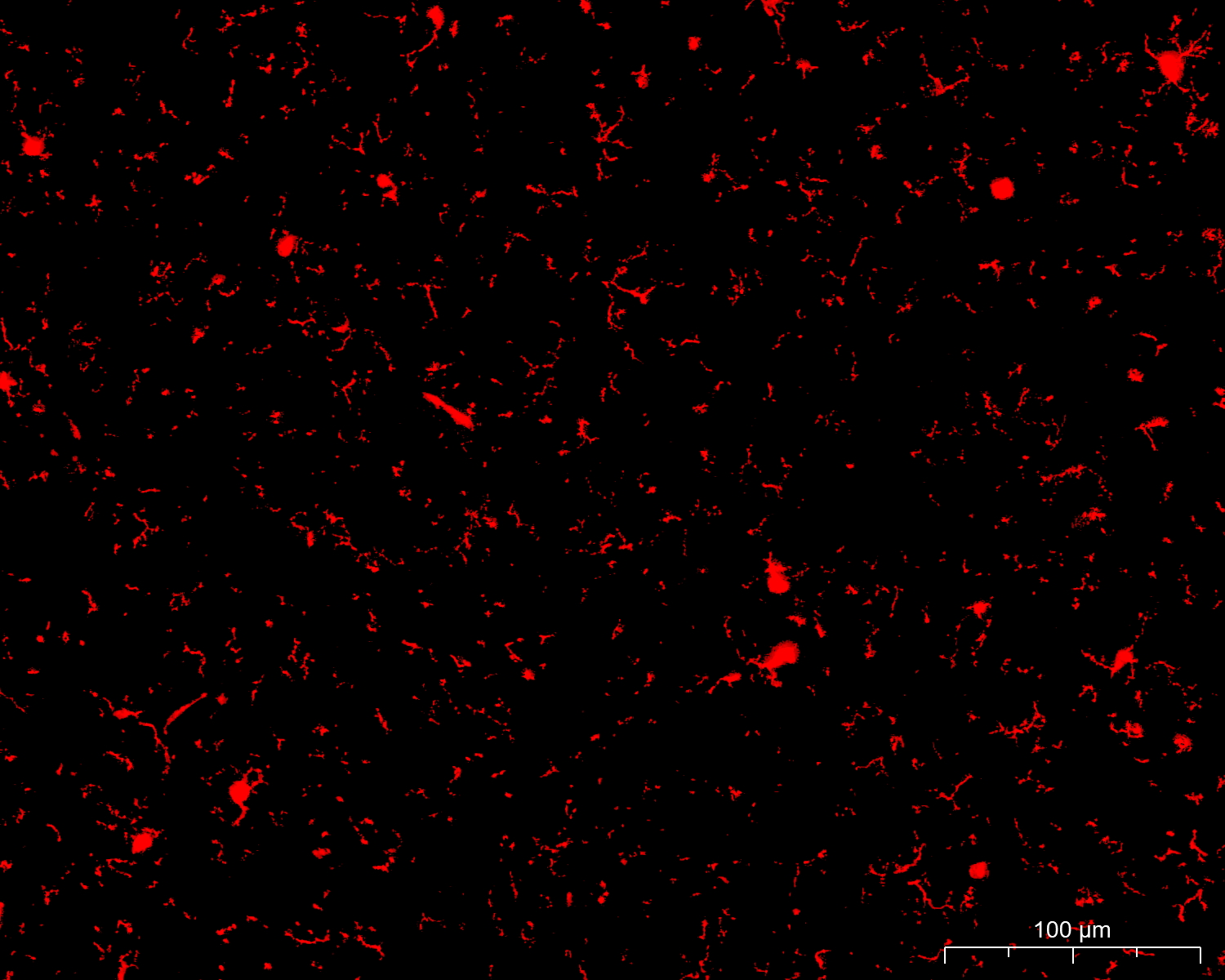

Supplement: Supplementary file 13 [file Data_Sheet_13.ZIP › ST/MR/2.jpg]

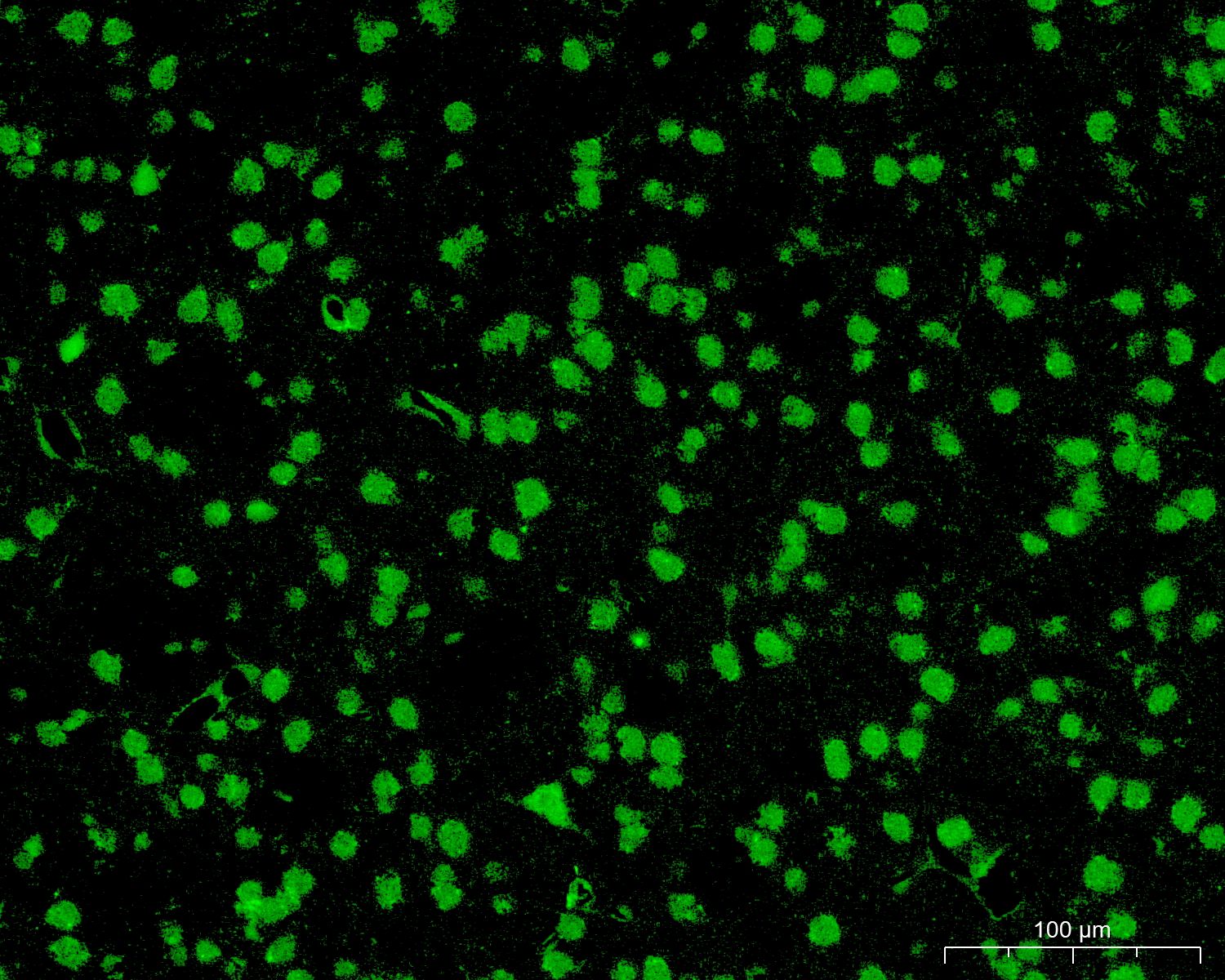

Supplement: Supplementary file 13 [file Data_Sheet_13.ZIP › ST/MR/3.jpg]

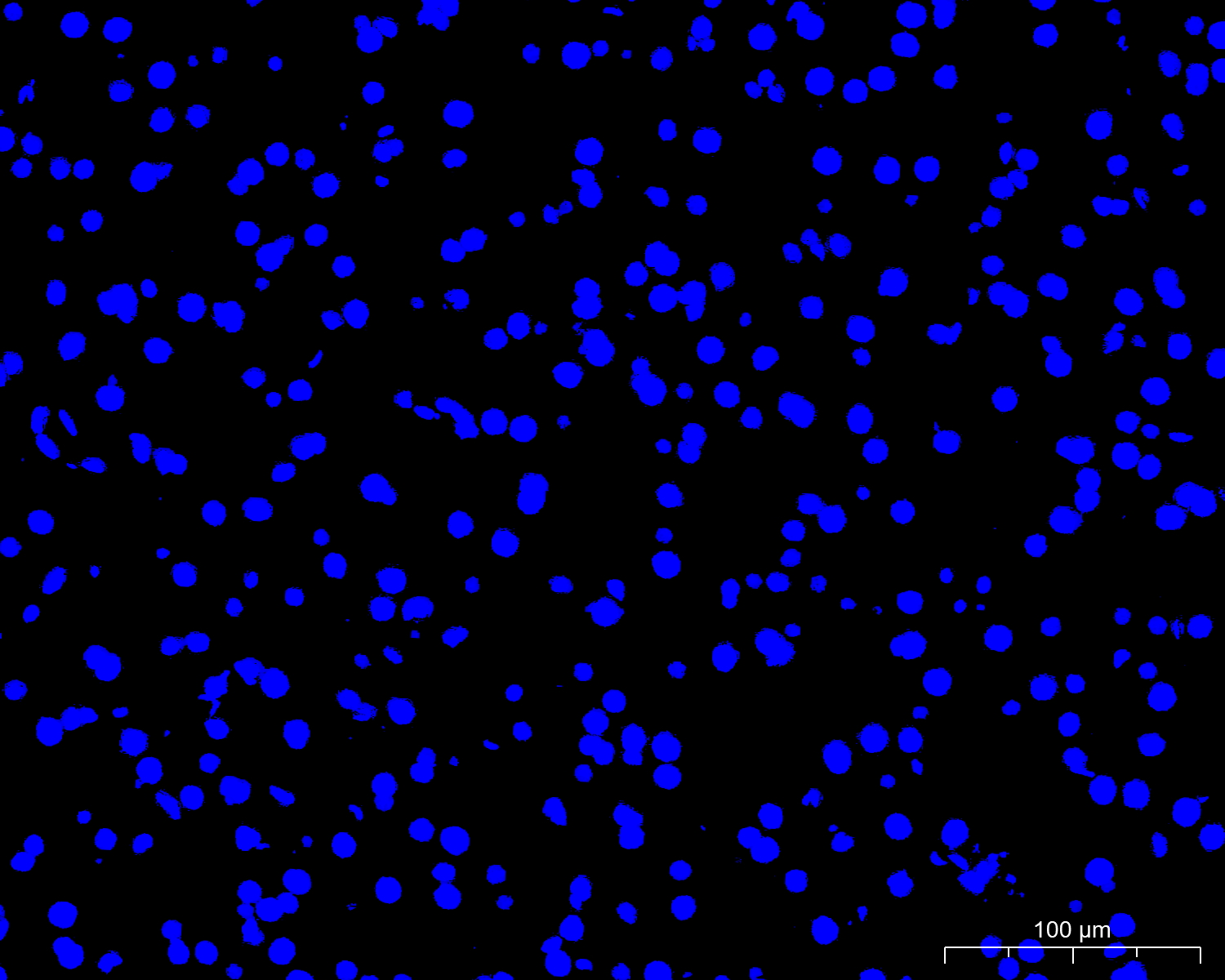

Supplement: Supplementary file 13 [file Data_Sheet_13.ZIP › ST/MR/4.jpg]

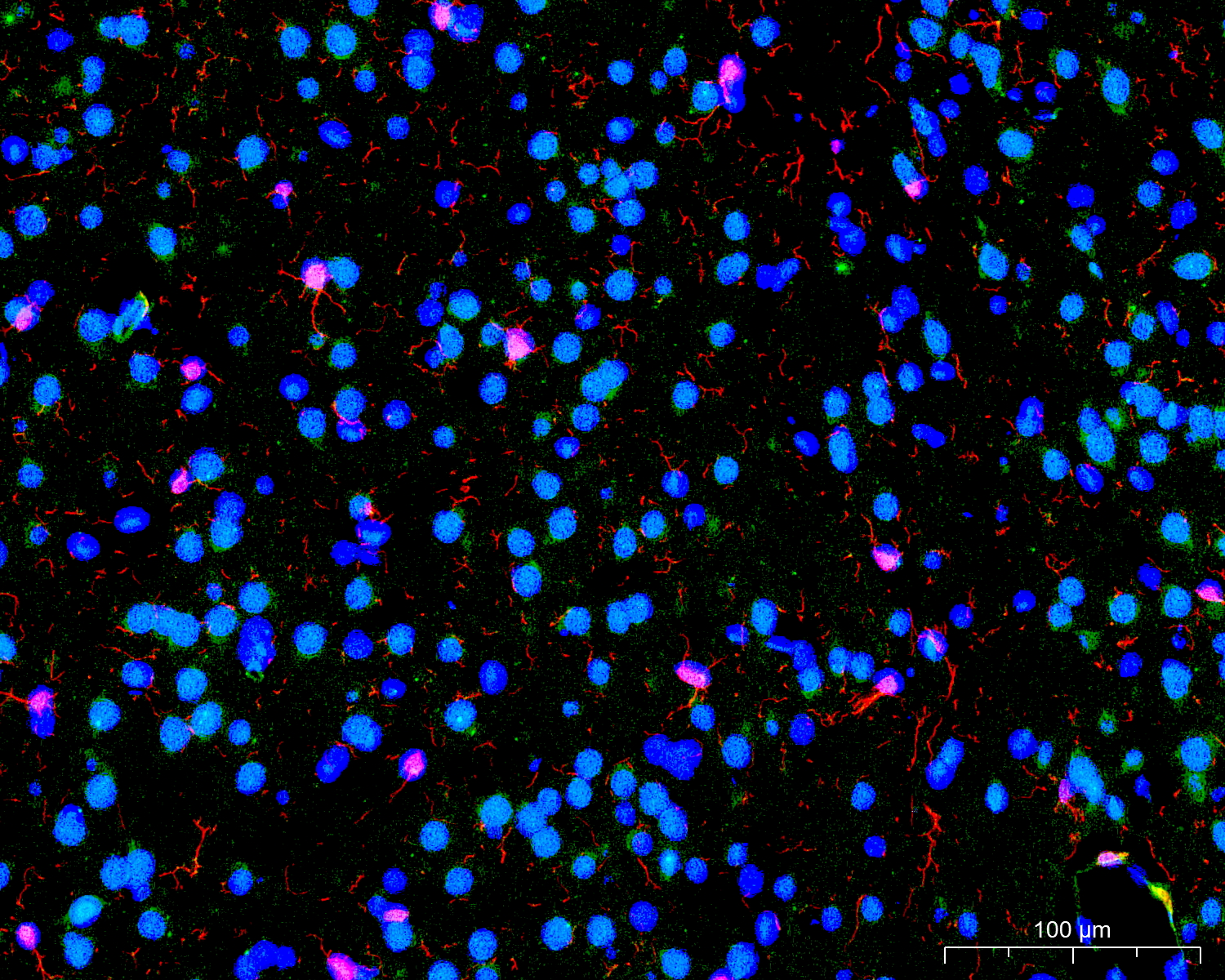

Supplement: Supplementary file 13 [file Data_Sheet_13.ZIP › ST/R/1.jpg]

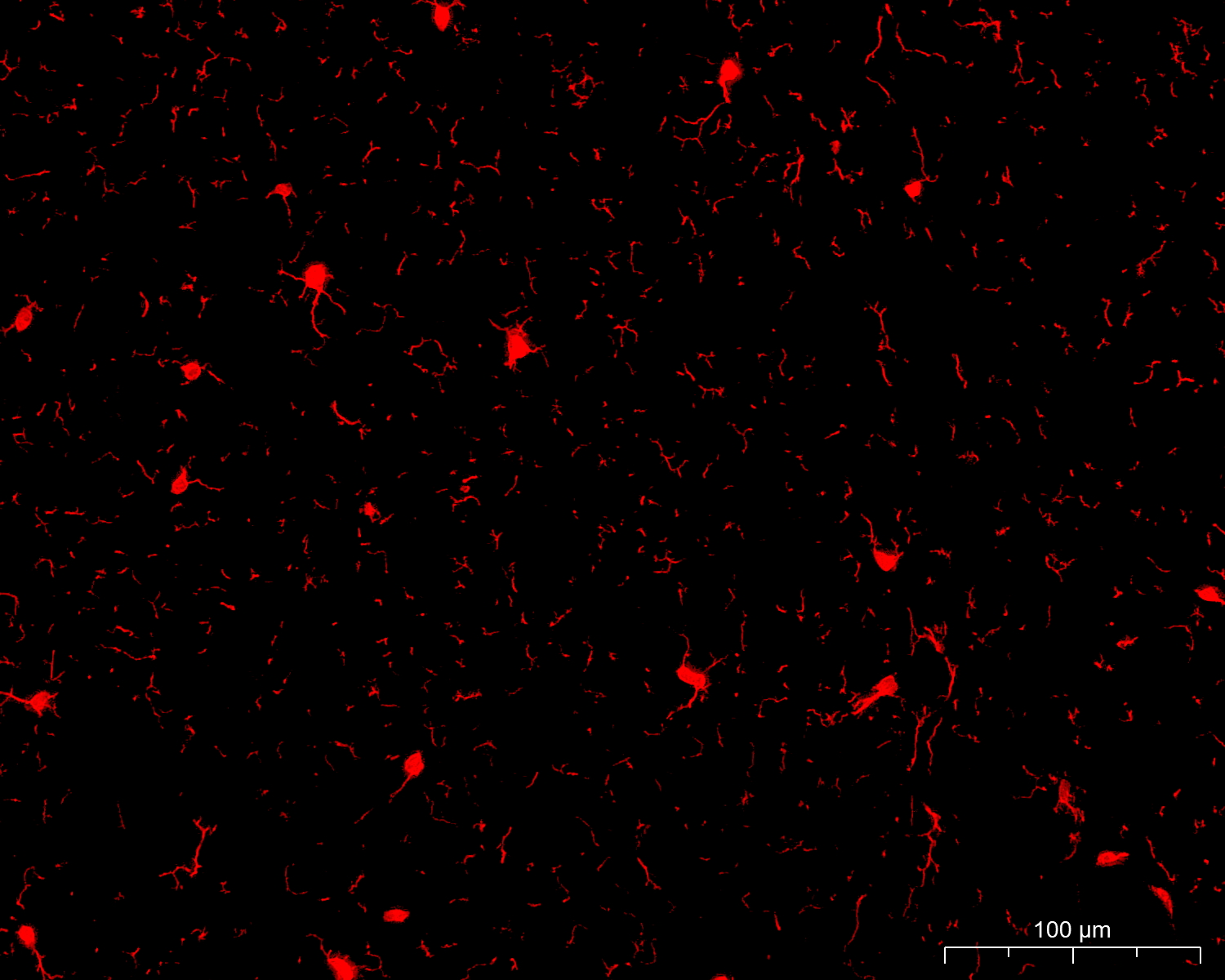

Supplement: Supplementary file 13 [file Data_Sheet_13.ZIP › ST/R/2.jpg]

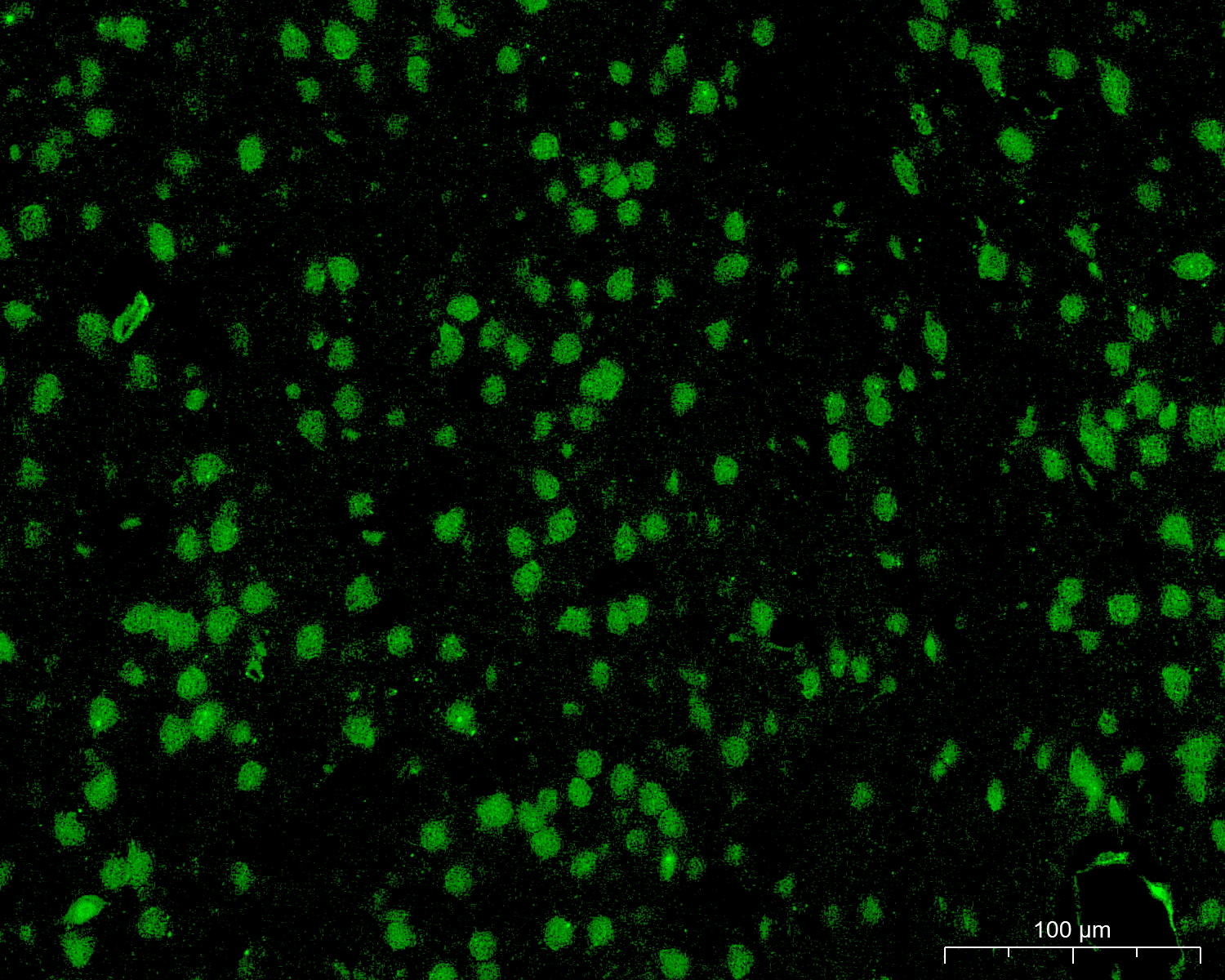

Supplement: Supplementary file 13 [file Data_Sheet_13.ZIP › ST/R/3.jpg]

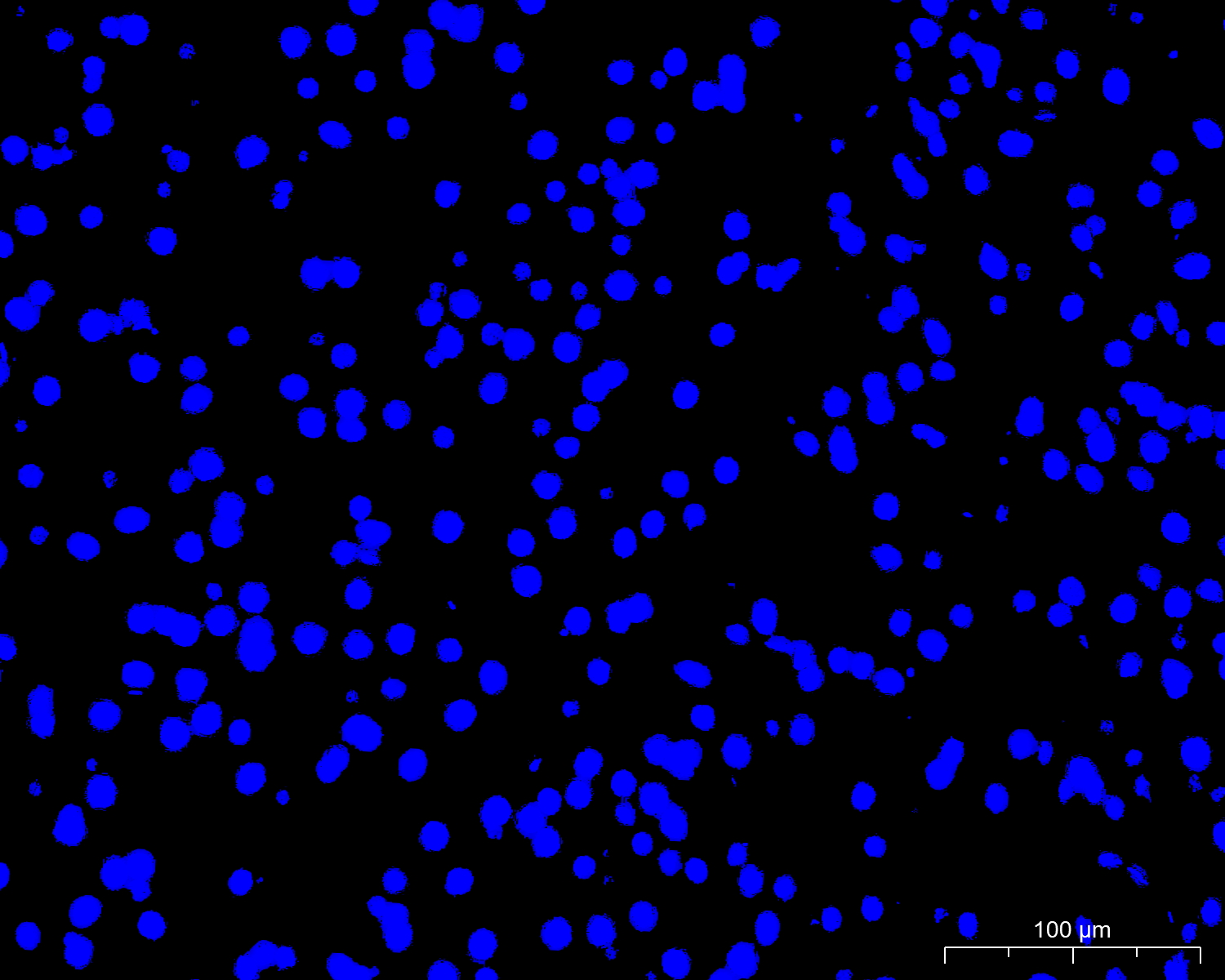

Supplement: Supplementary file 13 [file Data_Sheet_13.ZIP › ST/R/4.jpg]
